# TGF-β Neutralization Enhances AngII-Induced Aortic Rupture and Aneurysm in Both Thoracic and Abdominal Regions

**DOI:** 10.1371/journal.pone.0153811

**Published:** 2016-04-22

**Authors:** Xiaofeng Chen, Debra L. Rateri, Deborah A. Howatt, Anju Balakrishnan, Jessica J. Moorleghen, Lisa A. Cassis, Alan Daugherty

**Affiliations:** 1 Laboratory of Cardiovascular Disease, Department of Cardiology, Taizhou Hospital, Wenzhou Medical University, Zhejiang, China; 2 Saha Cardiovascular Research Center, University of Kentucky, Lexington, Kentucky, United States of America; 3 Department of Pharmacology and Nutritional Sciences, University of Kentucky, Lexington, Kentucky, United States of America; 4 Department of Physiology, University of Kentucky, Lexington, Kentucky, United States of America; Brigham and Women's Hospital, Harvard Medical School, UNITED STATES

## Abstract

AngII and TGF-β interact in development of thoracic and abdominal aortic diseases, although there are many facets of this interaction that have not been clearly defined. The aim of the present study was to determine the effects of TGF-β neutralization on AngII induced-aortic pathologies. Male C57BL/6J mice were administered with either a rabbit or mouse TGF-β neutralizing antibody and then infused with AngII. The rabbit TGF-β antibody modestly reduced serum TGF-β concentrations, with no significant enhancements to AngII-induced aneurysm or rupture. Administration of this rabbit TGF-β antibody in mice led to high serum titers against rabbit IgG that may have attenuated the neutralization. In contrast, a mouse TGF-β antibody (1D11) significantly increased rupture in both the ascending and suprarenal aortic regions, but only at doses that markedly decreased serum TGF-β concentrations. High doses of 1D11 antibody significantly increased AngII-induced ascending and suprarenal aortic dilatation. To determine whether TGF-β neutralization had effects in mice previously infused with AngII, the 1D11 antibody was injected into mice that had been infused with AngII for 28 days and were observed during continued infusion for a further 28 days. Despite near ablations of serum TGF-β concentrations, the mouse TGF-β antibody had no effect on aortic rupture or dimensions in either ascending or suprarenal region. These data provide further evidence that AngII-induced aortic rupture is enhanced greatly by TGF-β neutralization when initiated before pathogenesis.

## Introduction

Aortic aneurysm is defined as a permanent dilation of the lumen and represents a potentially fatal condition due to its high risk for rupture [1]. Aortic aneurysms are commonly located at specific regions within the thoracic and abdominal aorta. Although thoracic and abdominal aortic aneurysms have distinct clinical presentations and pathological features, an aberrant renin-angiotensin system (RAS) has been invoked in the pathogenesis of both regions [2]. This includes many studies demonstrating that AngII infusion promotes formation of aneurysm and rupture in both ascending and suprarenal aortic regions in mice [3–9]. Conversely, inhibition of AngII stimulation through either pharmacological antagonism of AT1 receptors or genetic disruption of AT1a receptors reduces both ascending and suprarenal aortic aneurysms in several animal models [8,10–12].

AngII-induced aortopathies have been associated with regulation of TGF-β signaling. TGF-β is a multifunctional cytokine that may mediate aortic diseases through complex pathways in both thoracic and abdominal regions [13,14]. Development of thoracic aortic aneurysm and rupture occurs in several mouse models of enhanced TGF-β pathway activity. These include mice with haploinsufficient [15] and hypomorphic [16] reductions in fibrillin1, expression of functionally deficient TGF-β1 and 2 receptors, [17] and inducible smooth muscle cell-specific deletions of TGF-β2 receptors [18,19].

Unlike antagonism of AngII, the effects of neutralizing TGF-β activity on aortopathies have been variable. In thoracic aortas, the effects of TGF-β neutralization have ranged from reducing aneurysms in fibrillin1 haploinsufficient mice [11] and AngII-infused CXCL10 deficient mice [15], while having no effect on aortic disease in mice expressing functionally deficient TGF-β receptors [20] to promoting ascending aortic rupture in fibrillin1 hypomorphic mice [12]. There is more limited data on the role of TGF-β in AAAs. TGF-β has been inferred to be detrimental based on increased expression of TGF-β1 and associated genes in abdominal aortic aneurysmal tissue of AngII-infused ApoE-/- mice [21]. However, local overexpression of TGF-β reduced AAAs in an explant model in rats [22]. In agreement with this protective effect of TGF-β, antibody-induced neutralization promoted abdominal aortic rupture in AngII-infused normocholesterolemic C57BL/6 mice [23]. Overall, the effects of TGF-β on aortopathies have been more variable than AngII.

AngII and TGF-β interact in the development of aortopathies, although there are many uncertainties regarding their mechanisms. These uncertainties include the variable effects on: 1. aortopathies in the thoracic versus abdominal area; 2. aortic dilatation versus rupture; 3. effects on aortic disease when neutralization was introduced after prolonged AngII infusion. Wang *et al*. [23] administered TGF-β neutralizing antibodies into AngII infused mice, providing a model to study these uncertainties. Therefore, we initially reproduced and then refined the approach used by Wang *et al*. [23]. Using this approach, we observed profound effects of TGF-β neutralization on significantly enhanced aortic rupture and dilatation in both thoracic and abdominal regions. Also, no effects of TGF-β neutralization were observed when instigated in mice previously infused with AngII.

## Materials and Methods

### Mice and Diets

Eight week old, male C57BL/6J mice were purchased from The Jackson Laboratory (Stock# 000664, Bar Harbor, ME, USA). Mice were group housed in individually vented cages with negative air exhaust pressure on a light:dark cycle of 14:10 hours. Rodent bedding consisted of Sani-Chip (Cat# 7090A, Harlan Teklad). Mice were fed a normal laboratory rodent diet (# 2918, Harlan Teklad) and provided with drinking water from a reverse osmosis system.

The recommendations in the Guide for the Care and Use of Laboratory Animals of the National Institutes of Health were followed. All procedures were approved by the Institutional Animal Care and Use Committee of the University of Kentucky (Approval # 2006–0009). Study mice were observed daily for normal behavior and weighed weekly throughout experimental duration. Humane endpoints included deteriorating body condition score, weight loss of >15%, inability to rise or ambulate, dyspnea, and dehydration. None of the study mice exhibited any of the humane endpoint criteria. All surgeries were performed under isoflurane anesthesia (Isothesia, NDC 11695-0500-2, Butler Schein) and post-surgery care utilized local topical analgesia (LMX4, NDC 0496-0882-15, Ferndale Laboratories) for minimizing stress and pain. Aortic rupture may occur in the AngII-infusion aneurysm mouse model (S1 Fig). In this study, none of the mice exhibited any hind leg bilateral paralysis prior to rupture. Mice were found dead and necropsied to determine site of rupture. At the end of study, mice were terminated with an overdose of anesthesia [ketamine (90 mg/kg, Ketathesia, Henry Schein, NDC 11695-0701-1) and xylazine [AnaSed, Lloyd Laboratories, NADA 139–236, 10 mg/kg).

### Pump Implantation and Antibody Injections

AngII (1,000 ng/kg/min; Cat# H-1706, Bachem) was infused via mini-osmotic pumps (Alzet Model #2004; Durect Corp) subcutaneously implanted on the flank of mice [24].

For the first study, mice were injected intraperitoneally at a dose of 10 mg/kg twice a week with either control rabbit nonimmune IgG antibody (Cat# AB-105-C, R&D Systems), or polyclonal rabbit TGF-β IgG antibody (Cat# AB-100-NA, R&D Systems). One week of IgG injections were given prior to the initiation of AngII infusion and the twice weekly injections were continued throughout the 28 day infusion of AngII (S2 Fig).

In a monoclonal TGF-β antibody dosing study, mice were injected intraperitoneally with mouse TGF-β IgG_1_ antibody or mouse IgG_1_ isotype control (Cat# MAB1835 and Cat# 11711, respectively, R&D Systems) at a range of doses (0, 0.03, 0.1, 0.3, 1, 5 mg/kg, 3 times per week) for 1 week. The dose of monoclonal TGF-β IgG_1_ antibody and mouse IgG_1_ isotype control in the subsequent study was selected based on the outcome of the dosage experiment (0.3 or 5 mg/kg, 3 times per week). One week of IgG injections were given prior to AngII infusion, and injections were continued throughout the 28 day infusion of AngII (S3 Fig).

To determine effects of TGF-β neutralization on aortas previously infused with AngII, either the monoclonal TGF-β monoclonal antibody or IgG_1_ control (5 mg/kg, 3 times per week) were injected into mice after 28 days of AngII infusion. During the period of antibody injections, mice were infused continuously with a new pump filled with AngII for an additional 28 days (S4 Fig).

### Analytical Assays

Serum TGF-β1 concentrations were measured using a sandwich enzyme immunoassay technique with an ELISA kit according to the manufacturer’s instructions (Cat# MB100B; R&D Systems). Serum samples were acidified with HCl (1 M) for 10 minutes to activate latent TGF-β1, and then neutralized with NaOH (0.5 M)/HEPES (1.2 N).

To examine whether rabbit IgG antibodies promoted an immune response to stimulate production of mouse anti-rabbit antibodies, serum concentrations of mouse anti-rabbit IgG titers were measured by ELISA. Sera from mice without antibody injection were used as negative controls. Plates were coated with normal rabbit IgG diluted in PBS and incubated overnight in a humidifying chamber. Serial dilutions of sera from study mice diluted in BSA-PBS (1% wt/vol) were added to the wells and incubated for 2 hours. Goat horseradish peroxidase (HRP) conjugated antimouse Ab (Cat# A2554, Sigma-Aldrich) was added and incubated for 30 minutes. Chromogen ABTS (2,2'-azino-di-(3-ethyl-benzthiazoline-6-sulphonic acid, Cat# A-9941, Sigma-Aldrich) was added to each well and read at 450 nm using a microplate reader.

Cell numbers in peripheral blood were counted using a Hemavet 950 (Drew Scientific Inc).

### Ultrasonic imaging

Lumenal diameters of suprarenal and ascending aortas were acquired using a Vevo 2100 with a MicroScan^TM^ transducer (MS400; VisualSonics). The resolution of this 18–38 MHz transducer is approximately 50 μm. Mice were anesthetized using Isoflurane^TM^ (Isothesia, Butler Animal Health Supply). Chest and abdominal hair was removed using clippers and depilatory cream (Nair; Church and Dwight Co). Ultrasonic gel (Medline; Mundelein) was placed on the area to be scanned. Two-dimensional images (B mode) of short-axis scans were acquired to measure the maximal diameter of suprarenal aortas and long-axis scans for maximal diameter of ascending aortas at selected intervals (S5 Fig).

### Ex Vivo Measurements of Aortas

At termination, mouse aortas were removed and fixed in 10% neutral-buffered formalin overnight at room temperature. Aortas were transferred to saline, adventitia removed, and then they were cut crosswise at the diaphragm region. After securing abdominal aortas with insect pins onto dental wax, the maximal ex vivo diameterswere measured (S6 Fig)[25]. For measurements of ascending dilation, thoracic aortas were cut open longitudinally in the inner curvature. Then the outer curvature from the proximal aorta to the subclavian arterial branch was cut. Aortas were secured with insect pins onto dental wax, and photographed. Intimal areas of ascending aortas were measured using an en face method with ImagePro software (S7 Fig; MediaCybernetics; Bethesda, MD, USA). [26,9].

### Statistics

Statistical analyses were performed using SigmaPlot Version 12 (SPSS Inc.). For two groups, comparisons were performed using an unpaired two-tailed Student’s t test for normally distributed continuous variables and a Mann-Whitney U test for non-normally distributed variables. One-way ANOVA with Student-Newman-Keuls was used to test a variable of more than 2 groups. A Fisher’s exact test was used to compare the incidence of aortic rupture. Two way ANOVA with Holm-Sidak post hoc analysis was performed for multiple-group and multiple-manipulation analyses. Kaplan-Meier survival curves were constructed and analyzed using log-rank (Mantel-Cox) test. P<0.05 was considered statistically significant.

## Results and Discussion

### Effects of a Rabbit Polyclonal anti-TGF-β Neutralizing Antibody on AngII-induced Aortopathies

**Fig 1:**
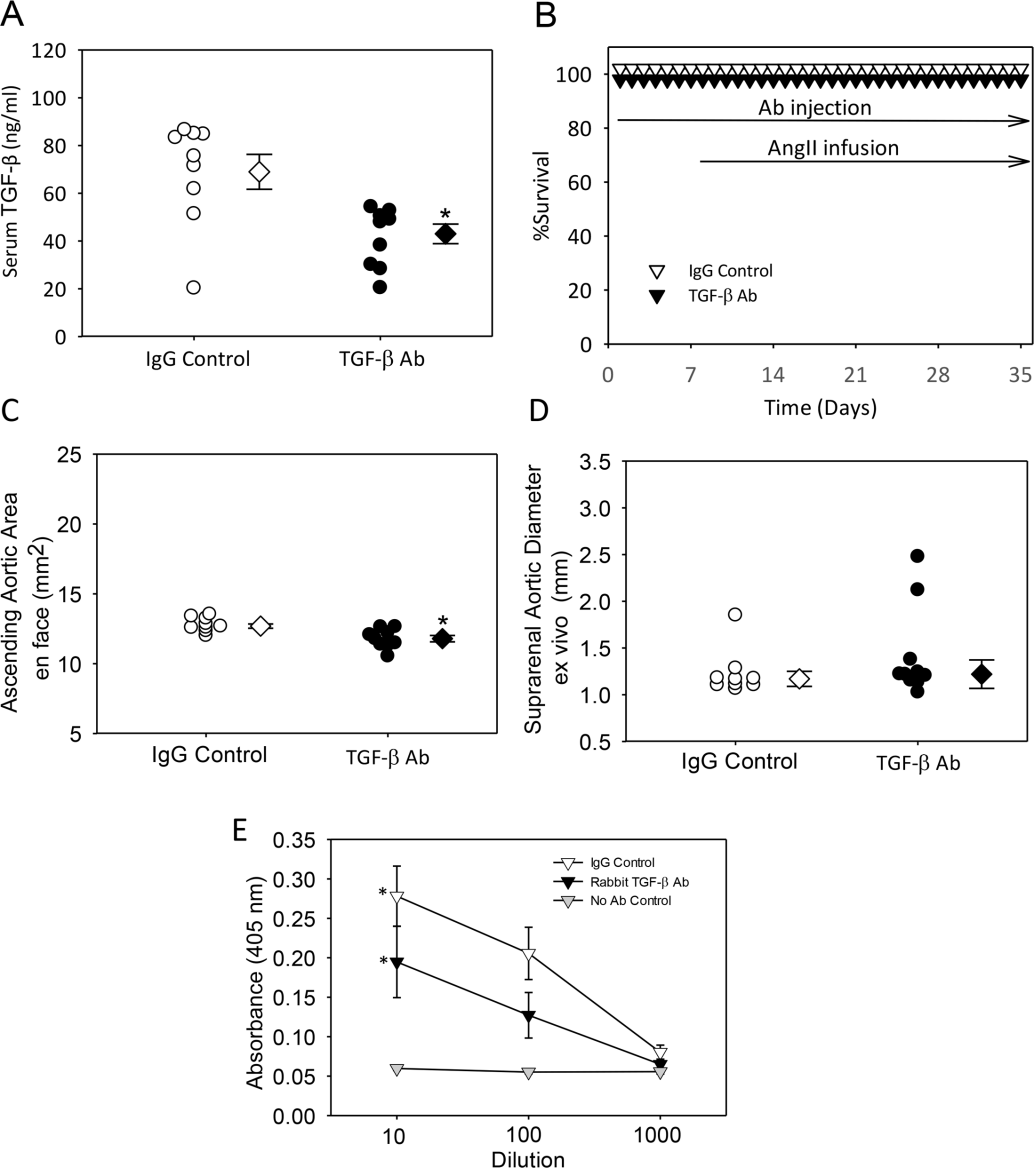
Minimal effects of rabbit TGF-β neutralizing antibody on aortic aneurysm. (A) Serum TGF-β concentration was measured at termination; N = 9 in control group and n = 9–10 in TGF- β group; *denotes P<0.05 by Mann-Whitney Rank Sum Test. (B) Kaplan-Meier curves of survival. (C) Ascending aortic area and (D) suprarenal aortic diameter were measured at termination. * denotes P< 0.05 by Student’s t-test. For (A), (C) and (D) circles are individual measurements, diamonds are means, and error bars are SEM,. (E) Serum mouse anti-rabbit titers in plasma were measured at termination in mice injected with Rabbit IgG control, TGF-β antibody or vehicle (No Ab Control). Triangles are means and error bars are SEM. * denotes P<0.05, compared to no antibody control by one way ANOVA on Ranks using Student-Newman-Keuls Method.

To determine the effects of TGF-β neutralization on ascending and suprarenal aortic aneurysm formation, we injected either IgG control or polyclonal rabbit TGF-β antibodies, at a dose of 10 mg/kg twice per week, into C57BL/6J mice infused with AngII. Injections were started 1 week prior to infusion of AngII. Injection of the rabbit TGF-β antibody had no overt detrimental effects on mouse health and body weight. Plasma renin was measured as an index of AngII potency, and as expected, was decreased significantly during AngII infusion (Table 1, Study 1). Since platelets are a major source of TGF-β, they were measured in whole blood to determine whether the neutralizing antibody would have an effect on their population. As predicted, platelet counts were not different between the groups (Table 1, Study 1). To determine the extent of neutralization achieved by injection of a TGF-β antibody, serum TGF-β was measured. Injections of the polyclonal rabbit TGF-β antibody modestly decreased serum TGF-β concentrations (P<0.05; Fig 1). This dosing regimen did not promote aortic rupture (Fig 1). Dilation of the ascending aorta decreased modestly, but significantly, (P<0.05) in mice injected with the polyclonal TGF-β antibody (Fig 1 and S8 Fig); while having no effects on AngII-induced abdominal aortic dilation (Fig 1 and S9 Fig).

**Table 1:** Characteristics of the mice.

	Study 1		Study 2				Study 3			
Ab Species	Rabbit		Mouse				Mouse			
Dose	10 mg/kg, 2/week		5 mg/kg, 3/week				0.3 mg/kg, 3/week		5 mg/kg, 3/week	
Infusion	AngII		Saline		AngII		AngII			
Antibody	IgG Control	TGF-β	IgG Control	TGF-β	IgG Control	TGF-β	IgG Control	TGF-β	IgG Control	TGF-β
N	9	10	10	10	20	20	10	10	10	10
Basal BW (g)	24.4±0.5	24.7±0.5	26.6±0.6	26.3±0.3	25.8±0.3	26.0±0.4	23.7±0.3	23.6±0.6	25.0±0.5	24.4±0.5
End point BW (g)	28.2±0.6	27.9±0.4	31.1±0.5	31.0±0.6	27.6±0.4*	27.6±0.6*	26.8±0.3	27.1±0.4	27.8±0.7	27.2±0.6
Renin (ng/ml)	1.2±0.1	1.1±0.0	2.3±0.3	2.1±0.3	1.3±0.03*	1.2±0.03*	ND	ND	ND	ND
Platelet (10^3^/μl)	650±81	693±143	838±45	830±35	796±37	905±65	723±86	725±28	757±47	711±57

Study #1: TGF-β inhibition on AngII-infused aneurysms using a rabbit polyclonal TGF-β antibody.

Study #2: TGF-β inhibition on AngII-infused aneurysms using a mouse monoclonal TGF-β antibody

Study #3: TGF-β inhibition on AngII-infused aneurysms using a low or high dose of a mouse monoclonal TGF-β antibody

AngII = Angiotensin II; BW = body weight

*P<0.05, compared to saline groups by two-way ANOVA test. ND, not determined.

A possible basis for the minimal effects of this antibody was attributable to the injection of a rabbit protein stimulating production of mouse anti-rabbit antibodies, thereby decreasing biological efficacy of this rabbit polyclonal TGF-β antibody. Therefore, we measured mouse antirabbit IgG antibodies using an ELISA method. Mice injected with the rabbit polyclonal TGF-β antibody had high serum titers of anti-rabbit antibodies. This immune response may contribute to the minor effects observed in the rabbit TGF-β antibody injected group (Fig 1).

### Effects of a Mouse TGF-β Monoclonal Neutralizing Antibody on AngII-induced Aortopathies

**Fig 2:**
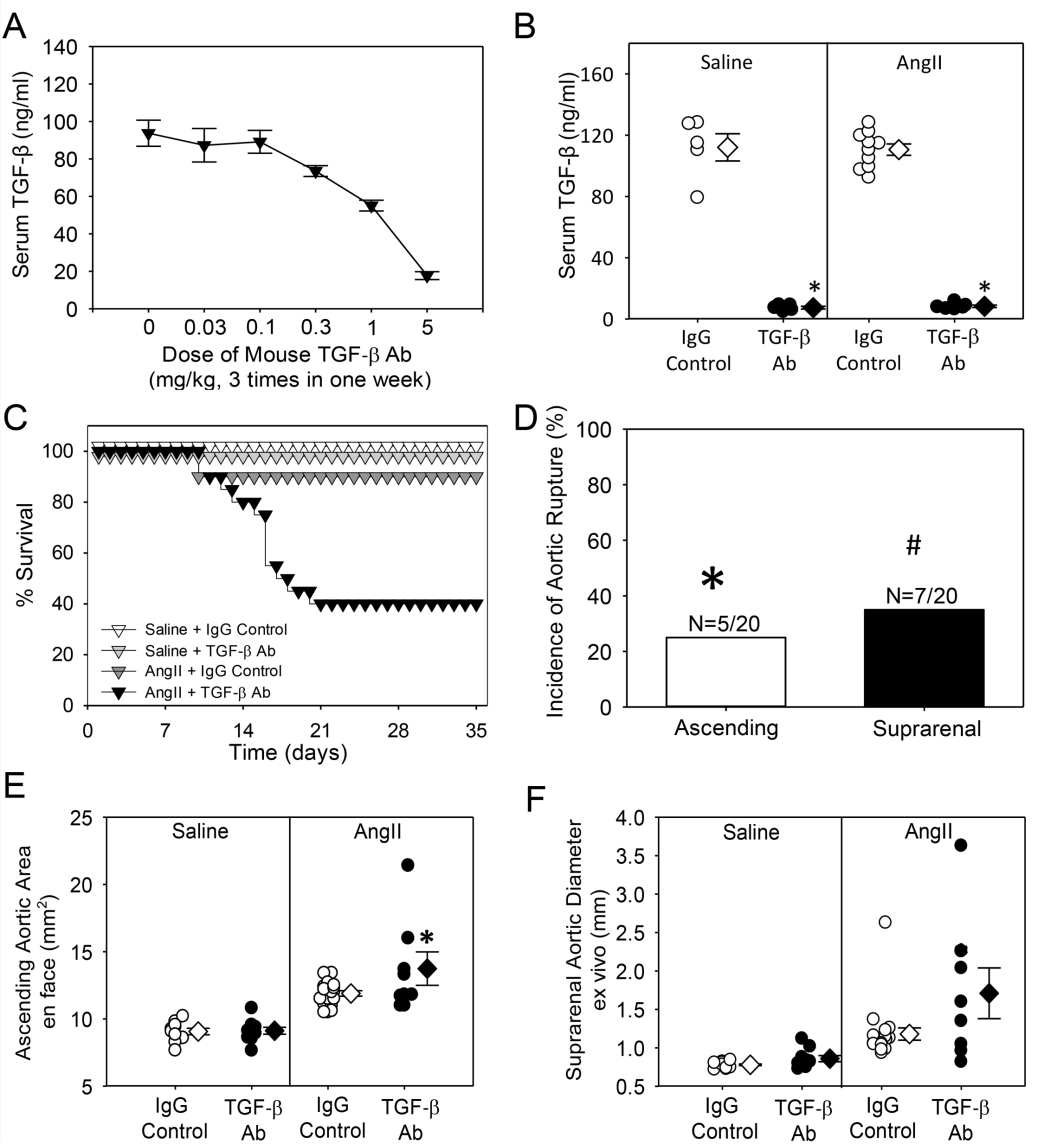
Mouse TGF-β neutralizing antibody promotes aortic rupture and aneurysm. (A) Serum TGF-β concentration was measured at termination in mice (N = 5/group) injected with different concentrations of TGF-β antibody. Triangles are means of the groups, and error bars are SEM. (B) Serum TGF-β concentration was measured at termination of study using 5 mg/kg, 3 times/week dose of antibody (Saline + IgG control, n = 5; Saline + TGF-β Ab, n = 5; AngII + IgG control, n = 10; AngII + TGF-β Ab, n = 5). * denotes P<0.05 by two way ANOVA. (C) Kaplan-Meier curves of survival. (D) Incidence of aortic rupture in the ascending and suprarenal aortas of AngII-infused mice injected with TGF-β antibody (n = 20 per group; * = P<0.05; # P<0.01). (E) Ascending aortic area and (F) suprarenal aortic diameter measured at experimental termination. * in denotes P = 0.017 in (E) and P = 0.002 in (F) when comparing TGF- β IgG versus control IgG groups within AngII by two way ANOVA. For (C), (E) and (F): Saline + IgG control, n = 10; Saline + TGF-β Ab, n = 10; AngII + IgG control, n = 19; AngII + TGF-β Ab, n = 8). For (B), (E), (F), circles are individual measurements, diamonds are means, and error bars are SEM.

To overcome a potential confounding immune response of injecting a cross-species antibody, we performed further studies using a mouse TGF-β neutralizing antibody (1D11) to examine its effects on AngII-induced ascending and suprarenal aortic aneurysms in C57BL/6J mice. First, we determined a dose of 1D11 that produced sustained reductions in serum TGF-β concentrations. Injections of 1D11 at 5 mg/kg, 3 times a week, substantially reduced serum TGF-β concentrations (81% reduction, P<0.001) in 1 week (Fig 2). In subsequent experiments, mice were injected with either the mouse TGF-β antibody or isotype-matched IgG control, at 5 mg/kg, 3 times per week, starting 7 days before infusion. Mice were infused with either saline or AngII for 28 days. As with the polyclonal antibody, injection of 1D11 had no effect on body weight, plasma renin concentration, and platelet numbers in whole blood (Table 1, Study 2). Administration of 1D11 significantly decreased serum TGF-β concentrations (P<0.001) with no difference between AngII and saline groups (Fig 2). While neutralization of TGF-β had no effect on rupture in saline-infused mice, it promoted aortic rupture in AngII-infused mice (Fig 2). Necropsies were performed rapidly on all mice that died during AngII infusion to determine region of rupture. Aortic rupture occurred equivalently in ascending and suprarenal aortic regions (Fig 2). In addition to rupture, we also measured aortic dimensions of survivors. Administration of 1D11 significantly increased AngII-induced ascending and suprarenal aortic dilation (Fig 2 and S10 and S11 Figs).

**Fig 3:**
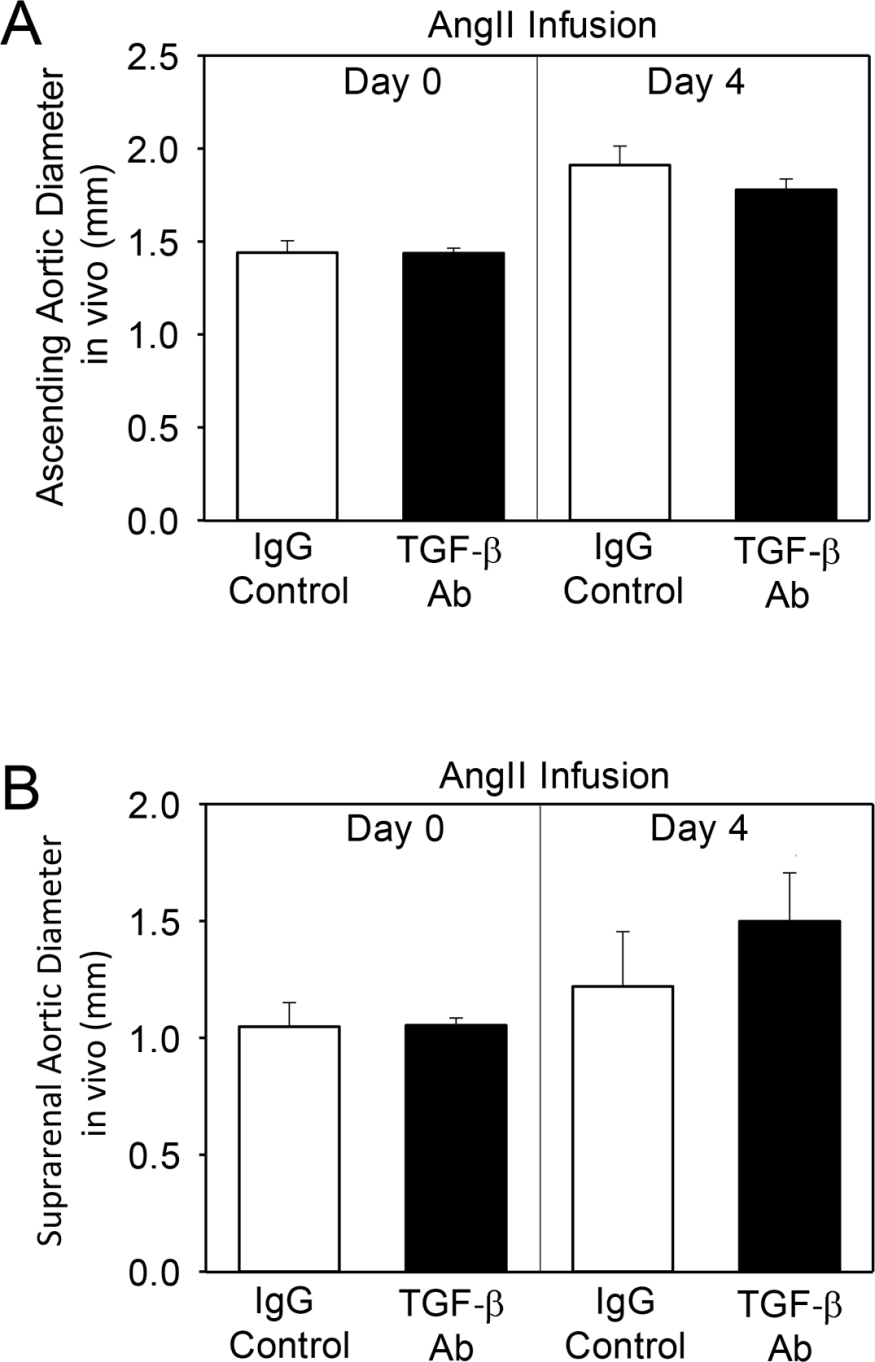
TGF-β neutralization (5 mg/kg, 3/week) had no effect on early ascending and suprarenal aortic dilation confirmed by *in vivo* ultrasonography. Aortas of infused mice were ultrasonically scanned at day 4 of infusion. (A) Aortas were imaged in the parasternal long axis view to obtain the measurements of ascending aortas. (B) Two-dimensional images (B mode) of short-axis scans were acquired to determine the maximal diameters of suprarenal aortas. Histobars represent mean ± SEM.

To examine whether there were any early changes in aortic dimensions during TGF-β neutralization, we used high-frequency ultrasound to measure diameters of ascending and suprarenal aortas prior to, and 4 days after AngII infusion in mice injected with either 1D11 or isotype-match IgG control. Although AngII promoted lumenal dilation in both aortic regions, 1D11 had no effects on early ascending (Fig 3 and S12 Fig) or suprarenal (Fig 3 and S13 Fig) aortic expansion.

**Fig 4:**
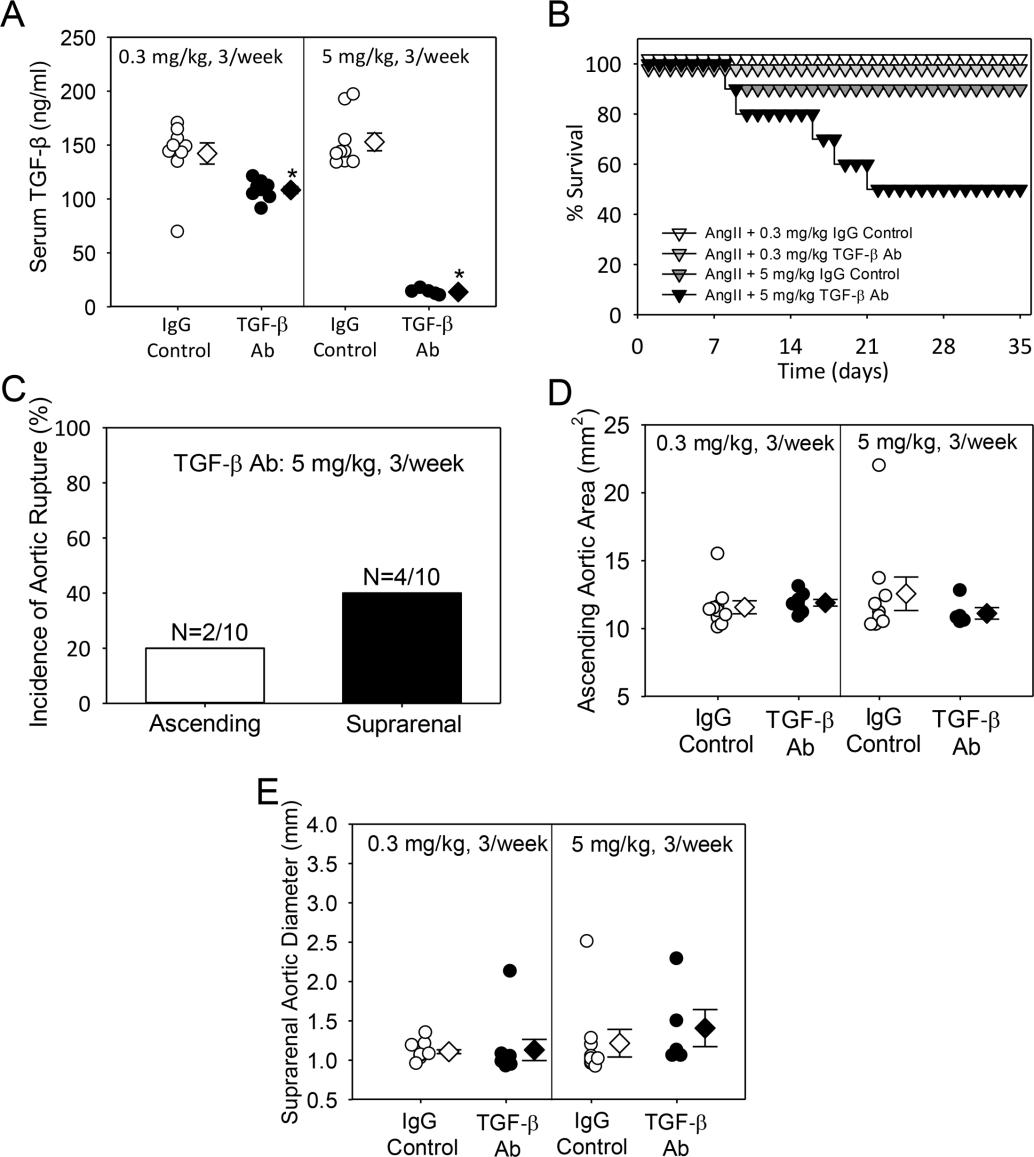
No divergent effect of low dose versus high dose of mouse TGF-β neutralizing antibody on aortic aneurysm and rupture. (A) Serum TGF-β concentration was measured at termination (N = 10 per group). * denotes P<0.05 by one way ANOVA. (B) Kaplan-Meier curves of survival. (C) Incidence of aortic rupture in the thoracic and abdominal aorta. (D) Ascending aortic area and (E) suprarenal aortic diameter was measured at termination. For (A), (D) and (E) circles are individual measurements, diamonds are means and error bars are SEM.

To determine whether TGF-β neutralization had dose-dependent divergent effects on aortopathies, we compared the effects of low (0.5 mg/kg, 3/week) and high (5 mg/kg, 3/week) doses of 1D11 on AngII-induced aortic aneurysms. Both low and high doses of 1D11 had no effect on body weight and platelet count (Table 1, Study 3). As expected, a low dose of 1D11 modestly decreased (40%) serum TGF-β concentration compared to the pronounced decrease (90%) of serum TGF-β concentration in the high dose group (Fig 4). While a low dose of 1D11 had no effect on aortic rupture, the high dose consistently promoted aortic rupture with incidence present in both ascending and suprarenal aortic regions (Fig 4). Both low and high doses of 1D11 had no effect on AngII-induced ascending area and suprarenal aortic expansion (Fig 4 and S14 and S15 Figs).

### TGF-β Neutralization Had no Effect on Aortic Aneurysm or Rupture in Mice Previously Infused with AngII

**Fig 5:**
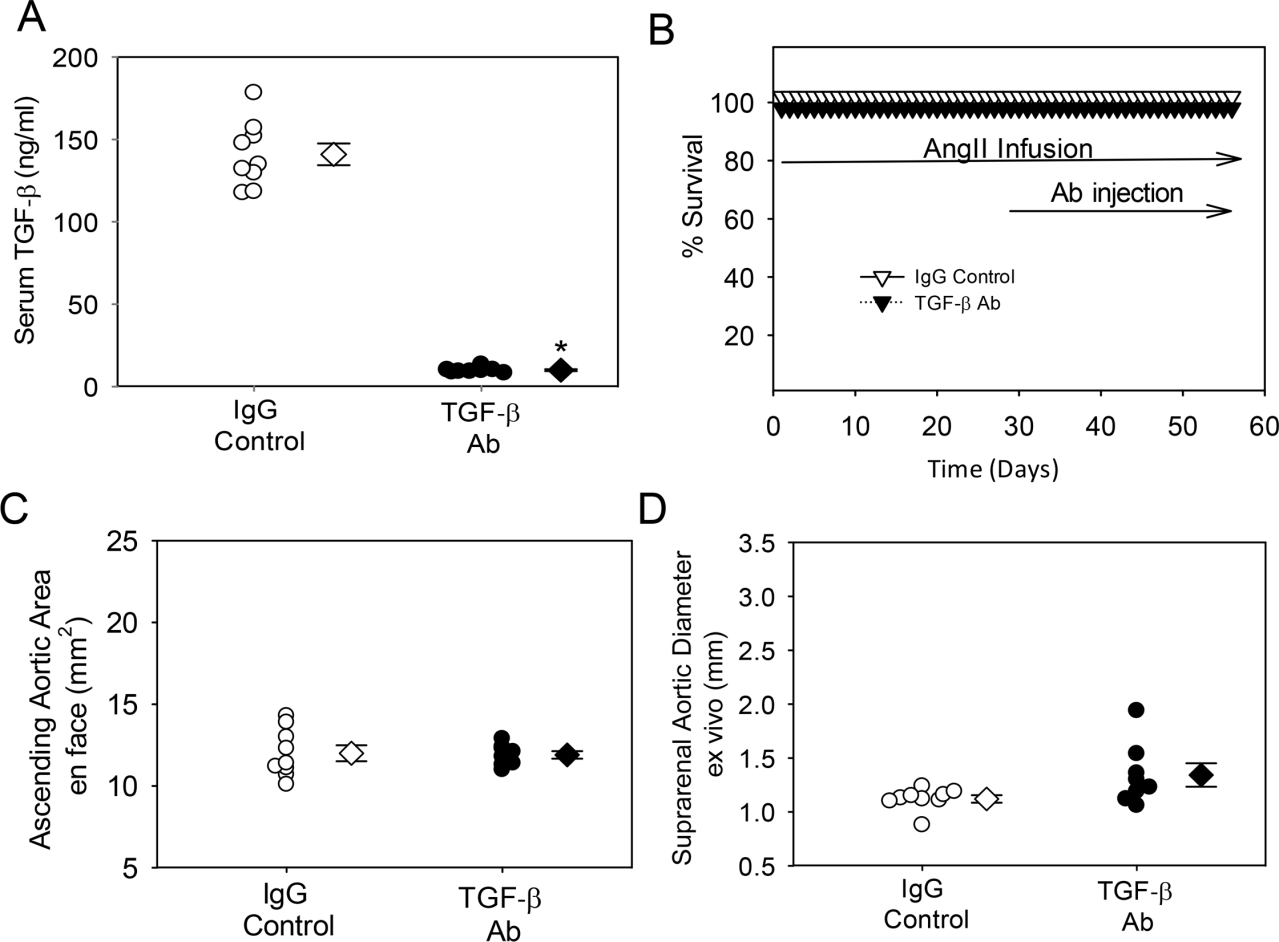
TGF-β neutralization had no effect on progression of aortic dilation and rupture. (A) Serum TGF-β concentration was measured at termination. * denotes P<0.05 by Student’s t-test. (B) Kaplan-Meier curves of survival. (C) Ascending aortic area and (D) suprarenal aortic diameter were measured at termination. For (A), (C) and (D) circles are individual measurements, diamonds are means, and error bars are SEM. (N = 9 for IgG control group and N = 8 for TGF-β Ab group).

In the above experiments, 1D11 was injected for 1 week before and during AngII infusion and therefore affected the initial stage of aortic rupture. Next, we examined whether TGF-β neutralization had effects in mice that were previously infused with AngII for 28 days, and then followed by administration of either 1D11 or IgG_1_ control into mice for a further 28 days. During the additional 28 day period of antibody injections, mice were continuously infused with AngII. As with previous studies, injection of 1D11 had no effect on body weight or platelet count (Table 2). Although the same high dose of 1D11 significantly decreased TGF-β1 serum concentrations to a similar degree in mice injected with the antibody before AngII infusion (Fig 5), it had no effect on the rate of aortic rupture (Fig 5). No difference in ascending or suprarenal aortic expansion between groups was observed (Fig 5 and S16 and S17 Figs).

**Table 2:** Characteristics of mice after 28 days of AngII infusion.

Group	Mouse IgG Control	Mouse TGF-β Ab
n	10	10
Dose of Ab	5 mg/kg, 3/week	5 mg/kg, 3/week
Basal BW (g)	25.4 ± 0.5	25.4 ± 0.4
End point BW (g)	28.0 ± 0.4	29.0 ± 0.4
Platelet (10^3^/μl)	796 ± 36	905 ± 65

AngII and TGF-β have complex interactions during development of aortic aneurysms. AngII inhibition by losartan in several mouse models of enhanced TGF-β activity completely suppressed ascending aorta aneurysm and rupture [11,12,17,18]. The converse of increasing AngII stimulation while reducing TGF-β activity has been demonstrated in only one study [23]. This study demonstrated that AngII infusion into mice, combined with TGF-β neutralization, had 90% incidence of aneurysm and rupture in normocholesterolemic C57BL/6 mice [23]. The present study reproduced the effects of TGF-β neutralization on enhancing AngII-induced aortic rupture, although only with a species-matched antibody. The present study extends the findings of Wang *et al*. [23] by demonstrating the need for a sufficiently high dose of antibody to produce major reductions in serum TGF-β concentrations to cause aortic pathology. Also, we demonstrate a significant increase in aortic rupture in both the ascending and suprarenal aortic regions, but only when TGF-β neutralization was initiated before AngII infusion.

Injection of antibodies has been a common approach to inhibit TGF-β activity. Previous studies demonstrated that injection of a rabbit TGF-β neutralizing antibody reduced diameter expansion of the ascending aorta of fibrillin1 haploinsufficient mice [11] and enhanced AngII-induced suprarenal aortic ruptures [23]. Using a rabbit TGF-β antibody provided by the same supplier, we were only able to achieve a minor reduction in serum TGF-β concentrations, with no profound change on aortic pathology in either region. Injection of the cross-species protein generated antibodies as demonstrated by high serum titers of anti-rabbit IgG. In the present study, the rabbit antibody was injected twice prior to start of AngII infusion. Therefore, it is likely that antirabbit IgG antibodies were present at the time of initiating AngII infusion and could have reduced the effectiveness of neutralization. This minimal effect of the rabbit TGF-β antibody was in stark contrast to the effects of a mouse monoclonal antibody and provides a cautionary note in using the former reagent. The effects of TGF-β neutralization using the 1D11 antibody to enhance ascending aortic rupture are not restricted to models of AngII infusion, since this antibody also increased ascending aortic rupture in young fibrillin1 hypomorphic mice [17]. Hence, while many facets of aortic rupture are unknown, there appears to be a common mechanism in these two mouse models.

While AngII infusion generates aneurysms in two distinct regions of the aorta, there are distinct pathologies between those present in the ascending versus the suprarenal region. AngII infusion leads to ascending aortic pathology with medial thickening, pronounced eccentric elastin fragmentation, and a paucity of infiltrating leukocytes [9]. In contrast, the pathology in the suprarenal aorta is characterized by focal medial rupture, adventitial thrombus, and accumulation of many types of leukocytes [4]. There are also region-specific functional differences. For example, TGF-β promotes smooth muscle cell growth in cells derived from thoracic origin, while inhibiting growth in cells derived from abdominal aorta [27]. Also, AngII exerts different contractile and medial thickening responses through the aorta [28–30]. Despite these regional differences between the ascending and suprarenal aorta, neutralization of TGF-β during AngII infusion led to similar increases in rupture and aneurysms in both regions.

Concomitant infusion of AngII with injection of a mouse TGF-β neutralizing antibody leads to significant increases in death due to aortic rupture in both ascending and suprarenal aortic regions. Aortic rupture usually occurs within days of initiating AngII infusion. The presence of rupture is presumed to be due to rapid destruction of the two major extracellular matrix proteins that maintain aortic integrity; collagen and elastin. The mouse TGF-β neutralizing antibody did not produce any aortic rupture when administration was initiated after 28 days of AngII infusion. Since AngII promotes profound medial and adventitial fibrosis [31], it is likely that the TGF-β mechanisms of rupture were inhibited by the enhanced fibrous nature of the aorta that occurs after protracted AngII infusion.

In summary, this study reproduced the major tenet of Wang *et al*. in demonstrating that TGF-β neutralization greatly accelerated aortic rupture in normocholesterolemic mice infused with AngII. This study provided refinement on optimizing antibodies and doses. In addition, the demonstration of uniformity in this effect is present in both regions prone to AngII-induced aortic pathology. The basis for TGF-β neutralization influencing aortic pathology during AngII infusion is unclear. However, it is likely that AngII promotes local secretion of TGF-β within the aortic wall. For example, AngII stimulates secretion of TGF-β from smooth muscle cells and fibroblasts which are major cell types in the aortic media and adventitia, respectively [32–34]. This action would be expected to promote fibrosis. To gain further mechanistic insight, future studies need to determine the TGF-β isoform involved in aortic rupture and delete it in a cell-specific manner in mice.

## Conclusions

In conclusion, TGF-β inhibition during AngII infusion augments expansion and rupture in both the thoracic and abdominal regions of the aorta.

## Supporting Information

S1 Fig **Representative images of (A) ascending aortic and (B) abdominal aortic rupture in mice infused with AngII.** Arrows point to thrombi which are dark red in color.
(PDF)

S2 Fig Experimental design for Study #1: Inhibition of TGF-β using a rabbit polyclonal IgG in male normolipidemic AngII-infused mice. Arrows denote single time points. Red box denotes continuous infusion in vivo. AngII = Angiotensin II (1,000 mg/kg/min). Ctrl = Isotype-matched control IgG.
(PDF)

S3 Fig Experimental design for Study #2, 3: Inhibition of TGF-β using a mouse monoclonal IgG (1D11) in male normolipidemic AngII-infused mice. Arrows denote single time points. Red box denotes continuous infusion in vivo. AngII = Angiotensin II (1,000 mg/kg/min). Ctrl = Isotype-matched control IgG.
(PDF)

S4 Fig Experimental design for Study #4: Inhibition of TGF-β using a mouse monoclonal IgG (1D11) in male normolipidemic mice previously infused with AngII. Arrows denote single time points. Red box denotes continuous infusion in vivo. AngII = Angiotensin II (1,000 mg/kg/min). Ctrl = Isotype-matched control IgG.
(PDF)

S5 Fig Diameter measurements of aortic regions in vivo imaged by ultrasound. A. The ascending area of the ascending aorta is indicated by tracing in teal. Diameters of proximal ascending aortas were measured (yellow line). Yellow asterisks indicate the innominate, common carotid and subclavian arterial branches of the aorta. B. The suprarenal abdominal aorta is indicated by tracing in teal. Diameters of suprarenal abdominal aortas were measured (yellow line).
(PDF)

S6 Fig Measurement of the abdominal aorta diameter. Representative photographs of saline and AngII-infused abdominal aortas. Aortas are harvested from mice, fixed overnight in formalin, cleaned of surrounding tissues, and cut in half at the diaphragm. The abdominal portions are pinned and photographed. Black lines depict maximal diameter measurements of suprarenal aortas. Numbers in white box are actual measurement in mm.
(PDF)

S7 Fig Measurement of the ascending aorta intimal area. Aortas were harvested from mice, fixed overnight in formalin and cleaned of surrounding tissue. Thoracic sections were cut open longitudinally along the inner curvature. Outer curvatures were subsequently cut longitudinally from the proximal ascending to the subclavian arterial branch (A). Aortas were pinned onto a wax dish and intimal area of the ascending aortas was measured between the yellow lines (B).
(PDF)

S8 Fig Images of ascending aortas ex vivo from AngII-infused mice in Study #1: Rabbit TGF-β neutralizing IgG experiment. Numbers below images are ascending aortic area measurements.
(PDF)

S9 Fig Images of abdominal aortas ex vivo from AngII-infused mice in Study #1: Rabbit TGF-β neutralizing IgG experiment. Numbers below images are suprarenal aortic diameter measurements.
(PDF)

S10 Fig Images of ascending aortas ex vivo from saline or AngII-infused mice in Study #2: Mouse TGF-β neutralizing IgG experiment. Numbers below images are ascending aortic area measurements.
(PDF)

S11 Fig Images of abdominal aortas ex vivo from saline or AngII-infused mice in Study #2: Mouse TGF-β neutralizing IgG experiment. Numbers below images are suprarenal aortic diameter measurements.
(PDF)

S12 Fig Baseline and Day 4 images of ascending aortas in vivo from mice infused with AngII and injected with mouse TGF-β neutralizing or control IgG. Teal lines indicate the outer curvature and diameter measurements of ascending aortas.
(PDF)

S13 Fig Baseline and Day 4 images of suprarenal aortas in vivo from mice infused with AngII for 4 days and injected with mouse TGF-β neutralizing or control IgG. Teal lines indicate the outer curvature and diameter measurements of ascending aortas.
(PDF)

S14 Fig Images of ascending aortas ex vivo from mice in Study #3: Injection of a low or high dose of mouse TGF-β neutralizing IgG. Numbers below images are ascending aortic area measurements.
(PDF)

S15 Fig Images of abdominal aortas ex vivo from mice in Study #3: Injection of a low or high dose of mouse TGF-β neutralizing IgG. Numbers below images are suprarenal aortic diameter measurements.
(PDF)

S16 Fig Images of ascending aortas ex vivo from mice in Study #4. Mice were infused with AngII for 28 days then injected with mouse TGF-β neutralizing IgG and infused with AngII for an additional 28 days. Numbers below images are ascending aortic area measurements.
(PDF)

S17 Fig Images of abdominal aortas ex vivo from mice in Study #4. Mice were infused with AngII for 28 days then injected with mouse TGF-β neutralizing IgG and infused with AngII for an additional 28 days. Numbers below images are suprarenal aortic diameter measurements.
(PDF)
