# Supplementary figures and images for: TGF-β Neutralization Enhances AngII-Induced Aortic Rupture and Aneurysm in Both Thoracic and Abdominal Regions
Source: PLoS One. 2016 Apr 22;11(4):e0153811. doi: 10.1371/journal.pone.0153811 (PMC4841552; doi:10.1371/journal.pone.0153811)

A

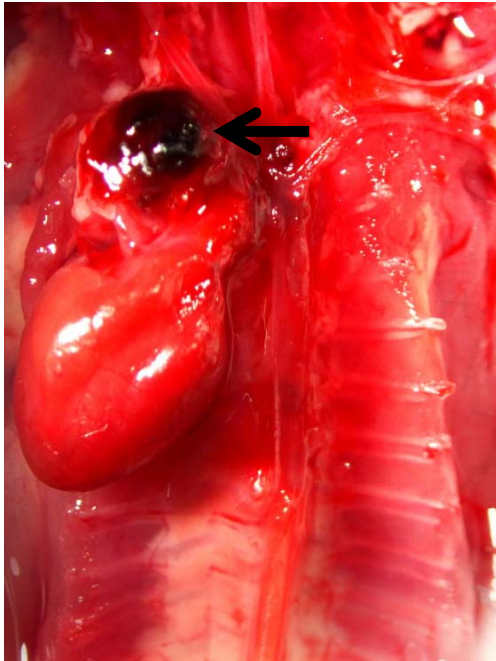

B

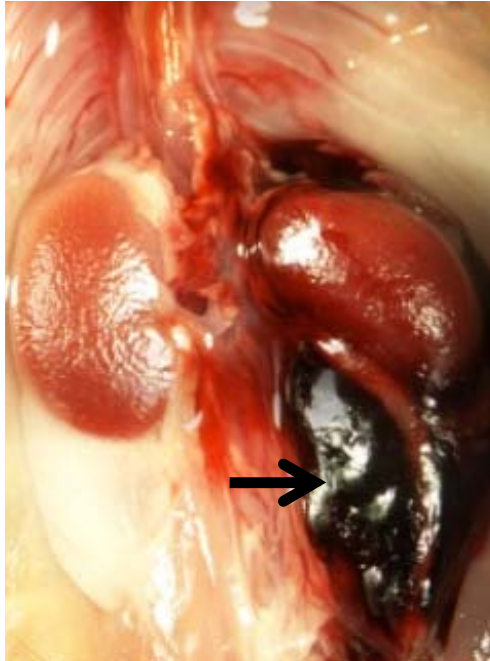

Supplement: S1 Fig — Representative images of (A) ascending aortic and (B) abdominal aortic rupture in mice infused with AngII. Arrows point to thrombi which are dark red in color. (PDF) [file pone.0153811.s001.pdf]

S5 Fig

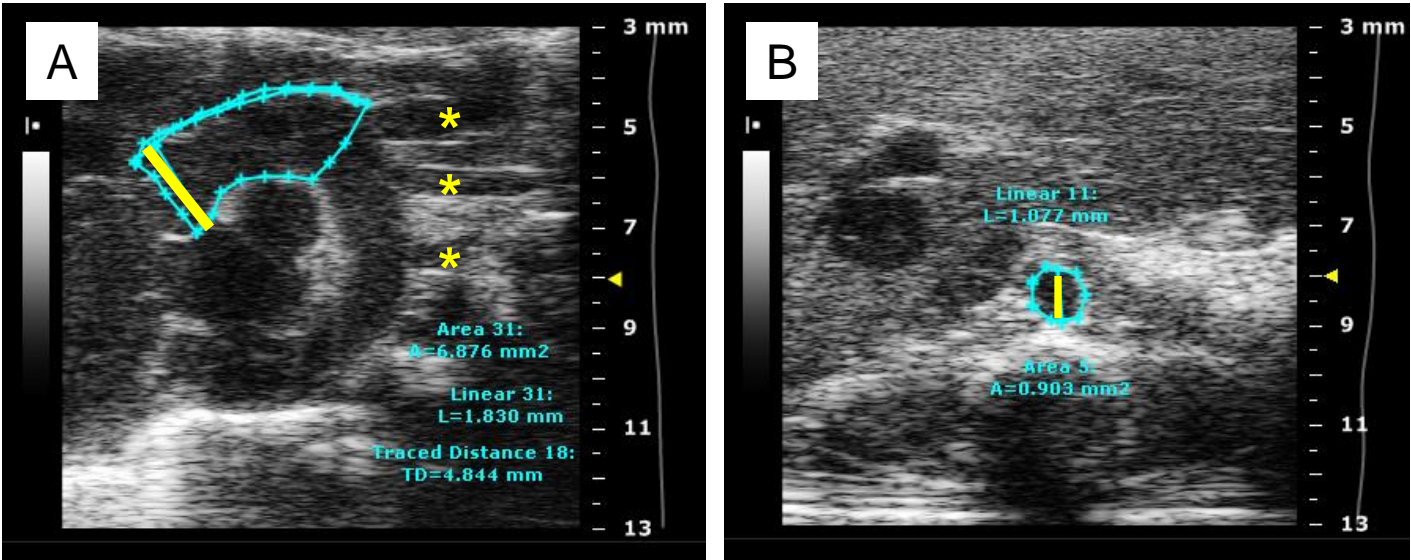

Supplement: S5 Fig — A. The ascending area of the ascending aorta is indicated by tracing in teal. Diameters of proximal ascending aortas were measured (yellow line). Yellow asterisks indicate the innominate, common carotid and subclavian arterial branches of the aorta. B. The suprarenal abdominal aorta is indicated by tracing in teal. Diameters of suprarenal abdominal aortas were measured (yellow line). (PDF) [file pone.0153811.s005.pdf]

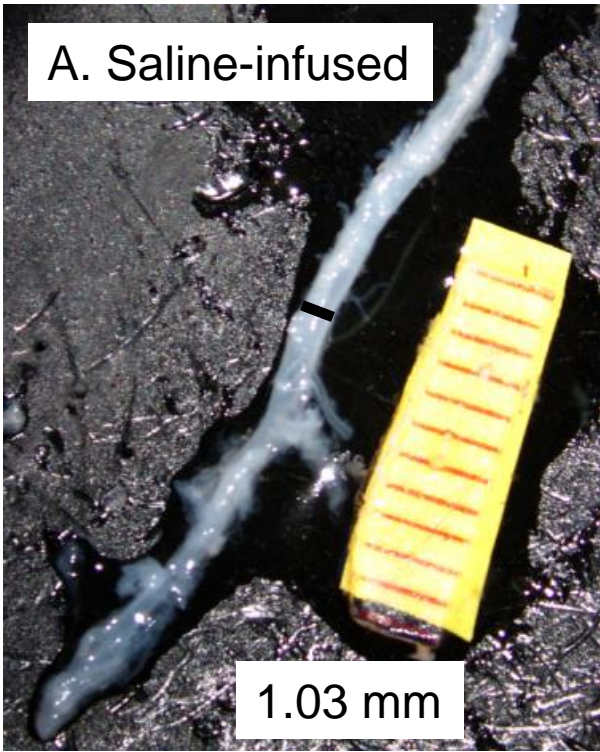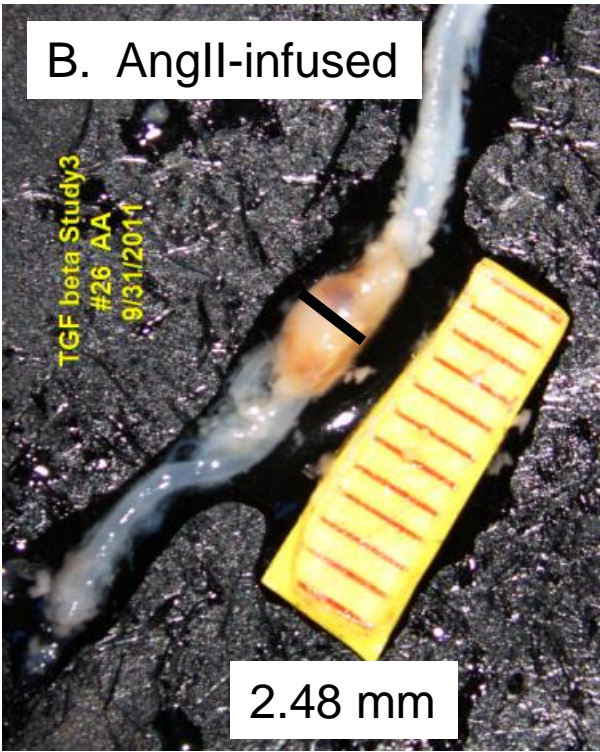

Supplement: S6 Fig — Representative photographs of saline and AngII-infused abdominal aortas. Aortas are harvested from mice, fixed overnight in formalin, cleaned of surrounding tissues, and cut in half at the diaphragm. The abdominal portions are pinned and photographed. Black lines depict maximal diameter measurements of suprarenal aortas. Numbers in white box are actual measurement in mm. (PDF) [file pone.0153811.s006.pdf]

S7 Fig

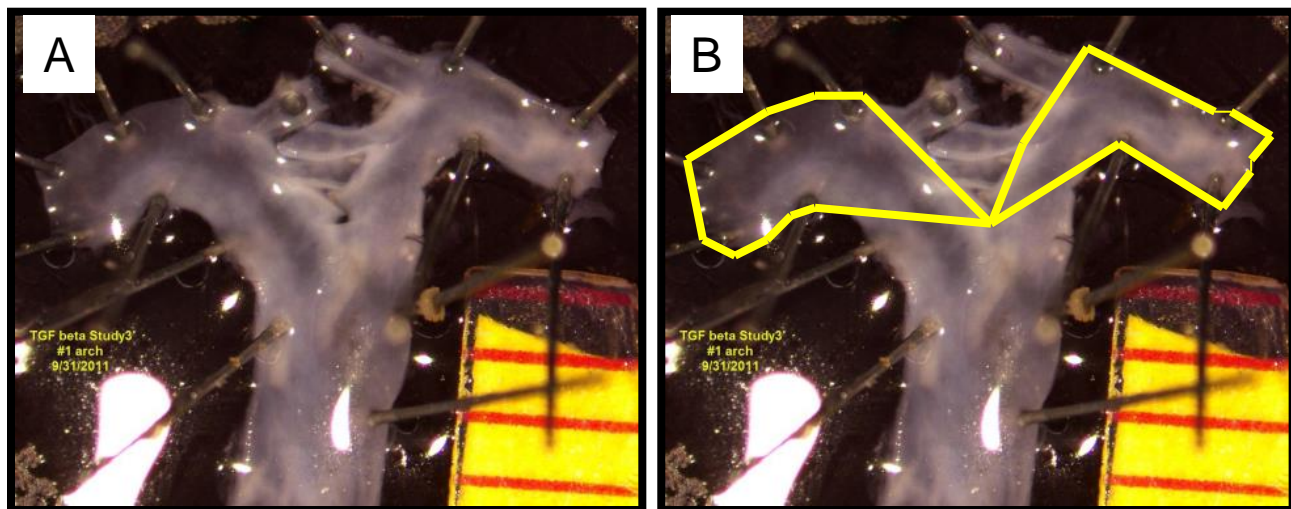

Supplement: S7 Fig — Aortas were harvested from mice, fixed overnight in formalin and cleaned of surrounding tissue. Thoracic sections were cut open longitudinally along the inner curvature. Outer curvatures were subsequently cut longitudinally from the proximal ascending to the subclavian arterial branch (A). Aortas were pinned onto a wax dish and intimal area of the ascending aortas was measured between the yellow lines (B). (PDF) [file pone.0153811.s007.pdf]
