# Supplementary material for: TGF-β Neutralization Enhances AngII-Induced Aortic Rupture and Aneurysm in Both Thoracic and Abdominal Regions
Source: PLoS One. 2016 Apr 22;11(4):e0153811. doi: 10.1371/journal.pone.0153811 (PMC4841552; doi:10.1371/journal.pone.0153811)
Supplement: S2 Fig — Arrows denote single time points. Red box denotes continuous infusion in vivo. AngII = Angiotensin II (1,000 mg/kg/min). Ctrl = Isotype-matched control IgG. (PDF) [file pone.0153811.s002.pdf]

S2 Fig

Study #1: Rabbit IgG

Group	Infusion	Injection (i.p.)		N
		IgG	Dose	
1	AngII	Control	10 mg/kg, 2/week	10
2	AngII	TGF-β Ab		10

Procedure	Time (Weeks)				
	0	1	2	3	4
Injection – IgG	↑↑	↑↑	↑↑	↑↑	↑↑
Infusion – AngII					
Serum TGF-β					↑
Aortic pathologies					↑
