# Supplementary material for: TGF-β Neutralization Enhances AngII-Induced Aortic Rupture and Aneurysm in Both Thoracic and Abdominal Regions
Source: PLoS One. 2016 Apr 22;11(4):e0153811. doi: 10.1371/journal.pone.0153811 (PMC4841552; doi:10.1371/journal.pone.0153811)

S3 Fig

Study #2,3: Mouse IgG

Group	Infusion	Injection (i.p.)		N
		IgG	Dose	
1	Saline	Isotype Ctrl	0.3 or 5 mg/kg, 3/week	10
2	Saline	TGF-β Ab		10
3	AngII	Isotype Ctrl		20
4	AngII	TGF-β Ab		20

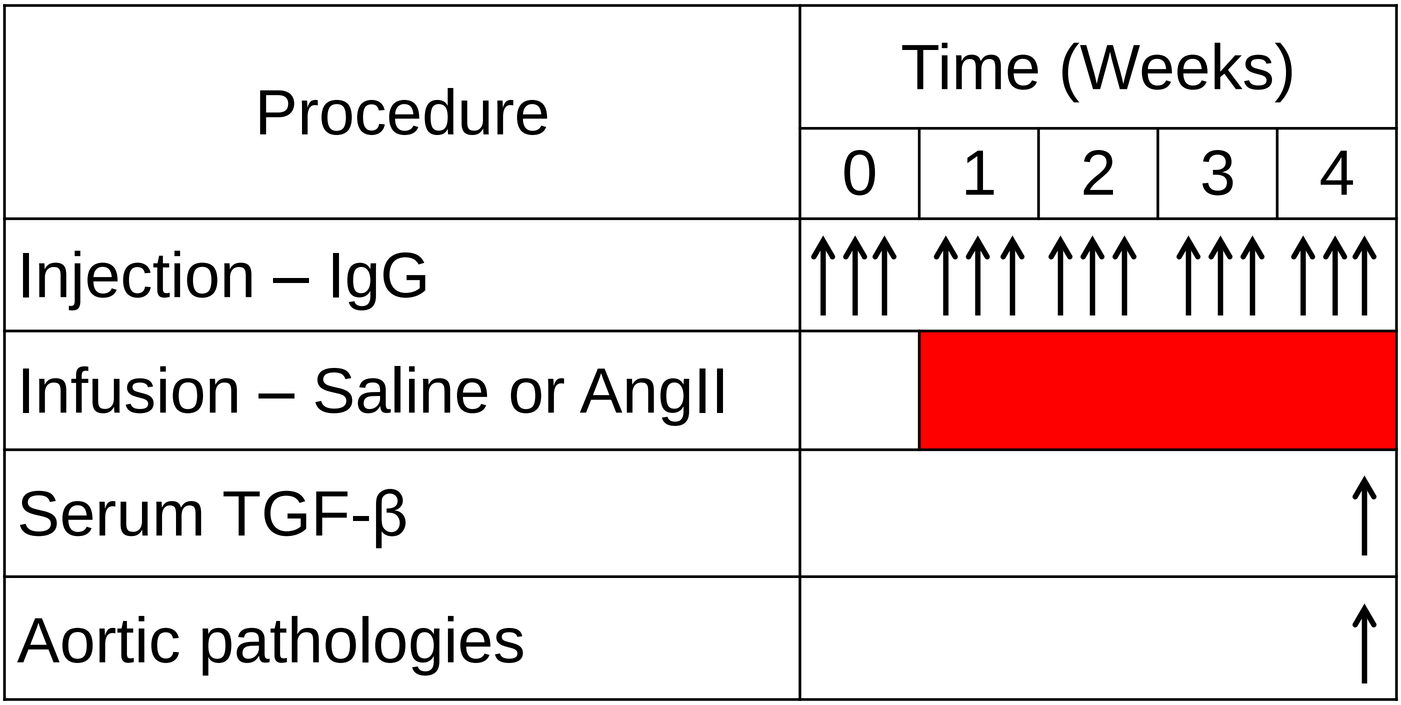

Supplement: S3 Fig — Arrows denote single time points. Red box denotes continuous infusion in vivo. AngII = Angiotensin II (1,000 mg/kg/min). Ctrl = Isotype-matched control IgG. (PDF) [file pone.0153811.s003.pdf]
