# Supplementary material for: TGF-β Neutralization Enhances AngII-Induced Aortic Rupture and Aneurysm in Both Thoracic and Abdominal Regions
Source: PLoS One. 2016 Apr 22;11(4):e0153811. doi: 10.1371/journal.pone.0153811 (PMC4841552; doi:10.1371/journal.pone.0153811)
Supplement: S4 Fig — Arrows denote single time points. Red box denotes continuous infusion in vivo. AngII = Angiotensin II (1,000 mg/kg/min). Ctrl = Isotype-matched control IgG. (PDF) [file pone.0153811.s004.pdf]

S4 Fig

Study #4: TGF-β inhibition in mice previously infused with AngII

Group	Infusion	Injection (i.p.)		N
		IgG	Dose	
1	AngII	Isotype Ctrl	5 mg/kg, 3/week	10
2	AngII	TGF-β Ab		10

	Time (weeks)							
Procedure	1	2	3	4	5	6	7	8
Infusion - AngII	↑				↑			
Ultrasonic scan				↑				
Injection - IgG					↑↑↑	↑↑↑	↑↑↑	↑↑↑
Serum TGF-β								↑
Aortic pathologies								↑
