# Supplementary material for: TGF-β Neutralization Enhances AngII-Induced Aortic Rupture and Aneurysm in Both Thoracic and Abdominal Regions
Source: PLoS One. 2016 Apr 22;11(4):e0153811. doi: 10.1371/journal.pone.0153811 (PMC4841552; doi:10.1371/journal.pone.0153811)
Supplement: S8 Fig — Numbers below images are ascending aortic area measurements. (PDF) [file pone.0153811.s008.pdf]

S8 Fig

Study #1: Control rabbit IgG (10 mg/kg, 2 times/week) AngII (1,000 ng/kg/min)

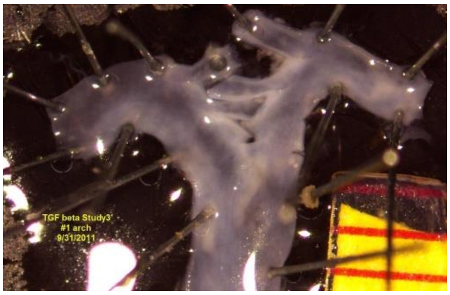

13.55 mm^2

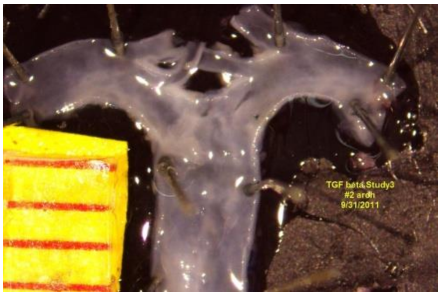

12.58 mm^2

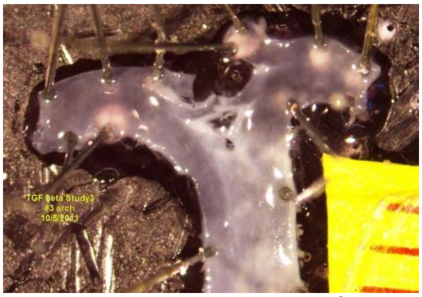

12.24 mm^2

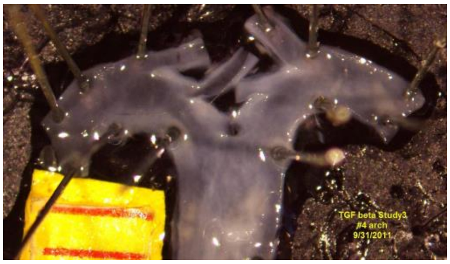

13.41 mm^2

5: Died

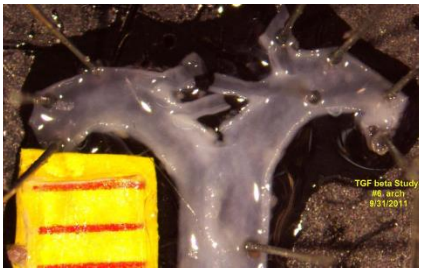

12.36mm^2

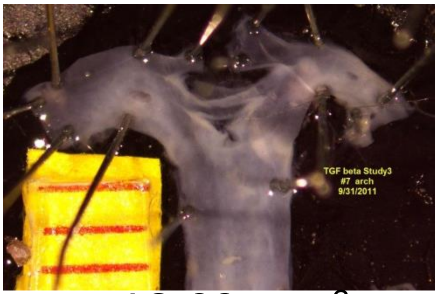

12.69 mm^2

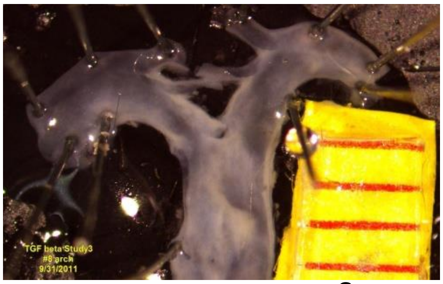

12.87 mm^2

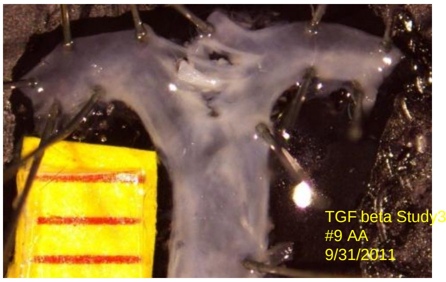

13.26 mm^2

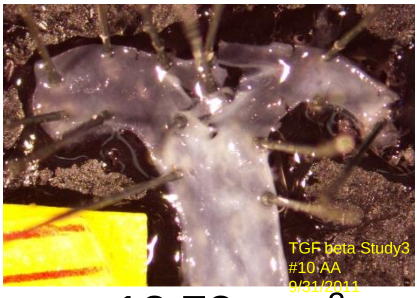

12.73 mm^2

Study #1: TGF-β rabbit IgG
(10 mg/kg, 2 times/week)
AngII (1,000 ng/kg/min)

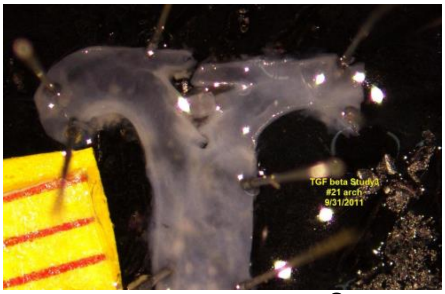

$10.54\ mm^2$

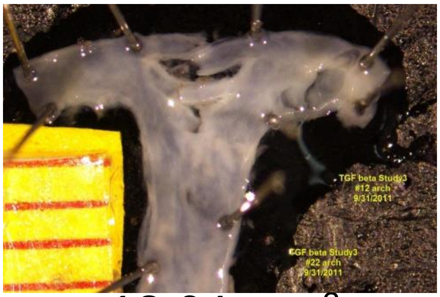

$12.64\ mm^2$

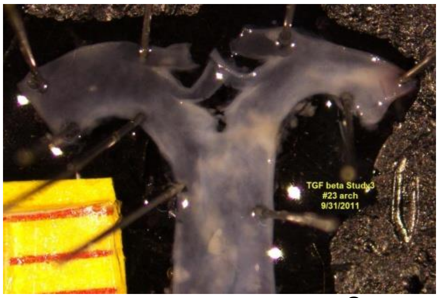

$11.49\ mm^2$

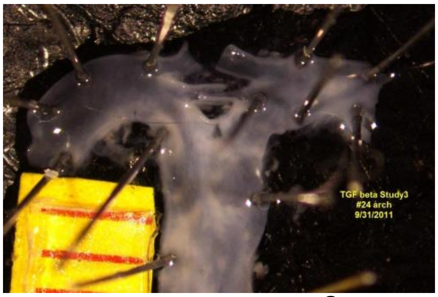

$12.21\ mm^2$

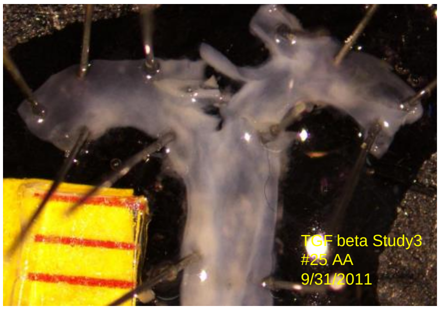

$12.65\ mm^2$

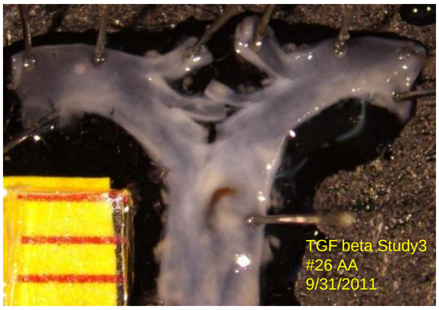

$11.3\ mm^2$

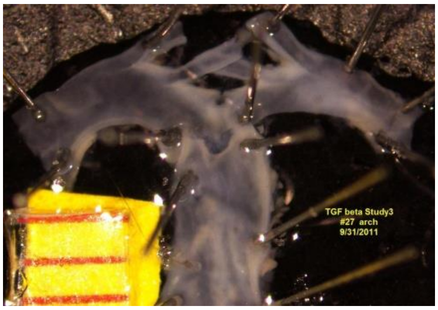

$11.38\ mm^2$

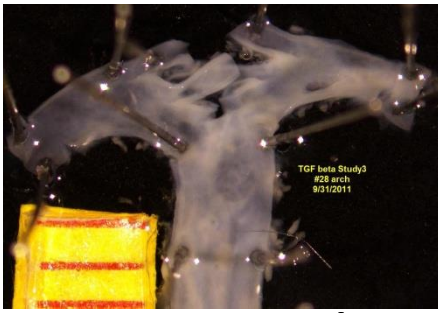

$12.08\ mm^2$

29: Died

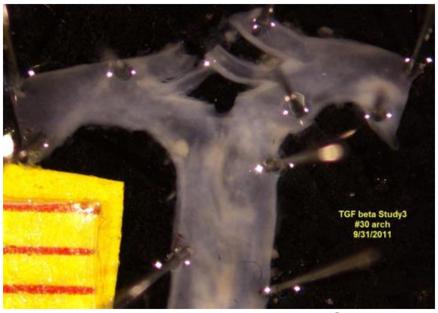

$11.67\ mm^2$
