# Supplementary material for: TGF-β Neutralization Enhances AngII-Induced Aortic Rupture and Aneurysm in Both Thoracic and Abdominal Regions
Source: PLoS One. 2016 Apr 22;11(4):e0153811. doi: 10.1371/journal.pone.0153811 (PMC4841552; doi:10.1371/journal.pone.0153811)
Supplement: S9 Fig — Numbers below images are suprarenal aortic diameter measurements. (PDF) [file pone.0153811.s009.pdf]

S9 Fig

Study #1: Control rabbit IgG
(10 mg/kg, 2 times/week)
AngII (1,000 ng/kg/min)

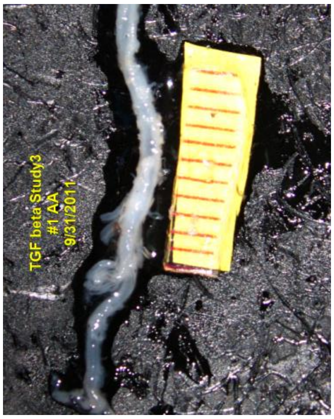

1.17 mm

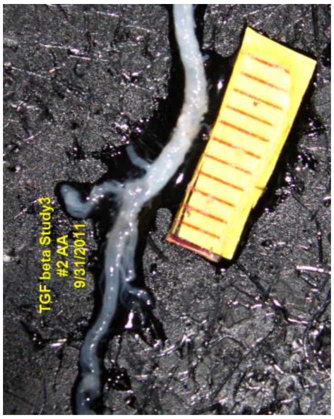

1.29 mm

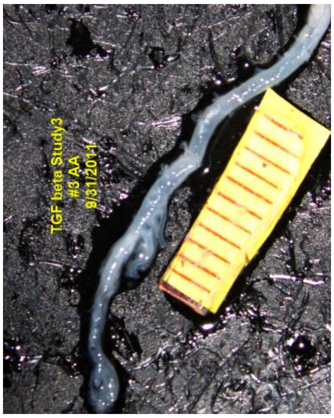

1.11 mm

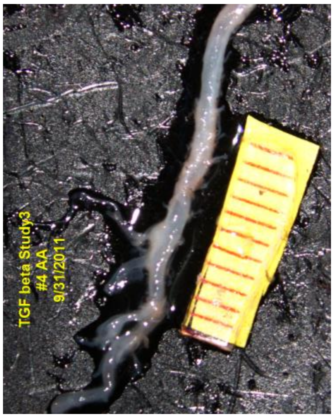

1.18 mm

5: Died

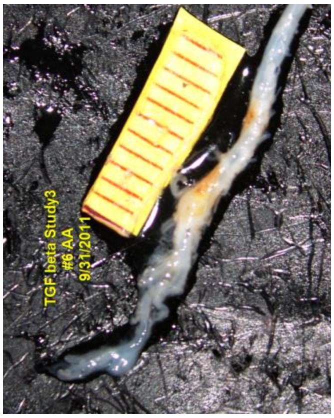

1.85 mm

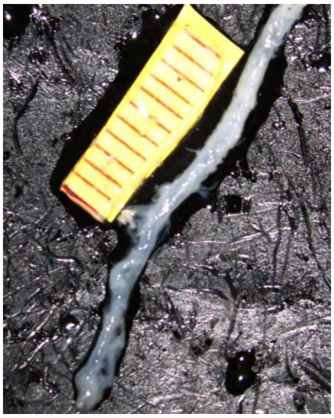

1.07 mm

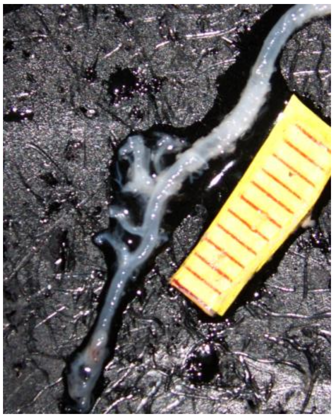

1.12 mm

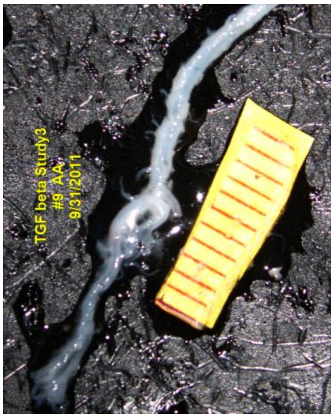

1.11 mm

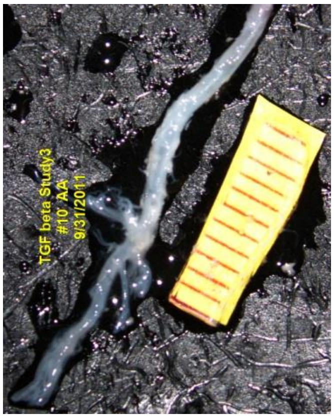

1.18 mm

Study #1: TGF-β rabbit IgG
(10 mg/kg, 2 times/week)
AngII (1,000 ng/kg/min)

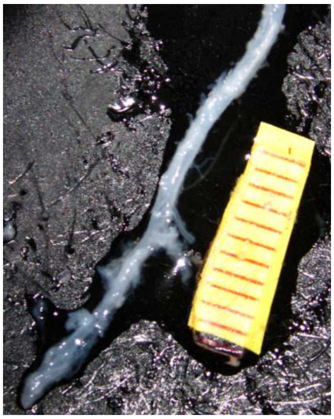

1.03 mm

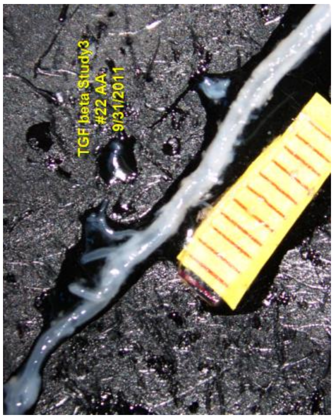

1.22 mm

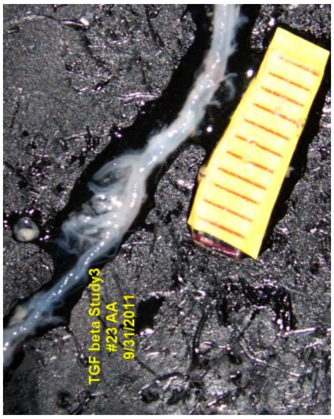

1.23 mm

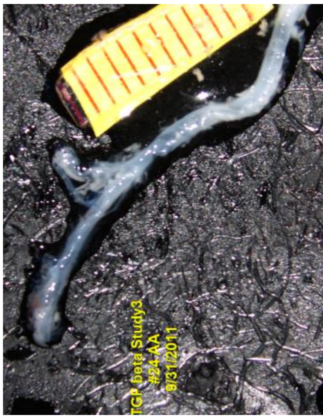

1.16 mm

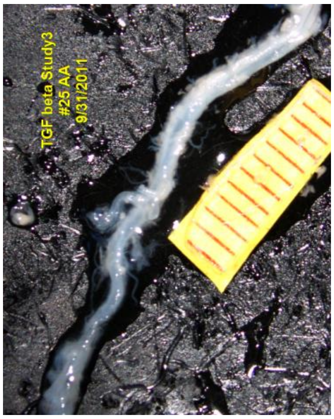

1.14 mm

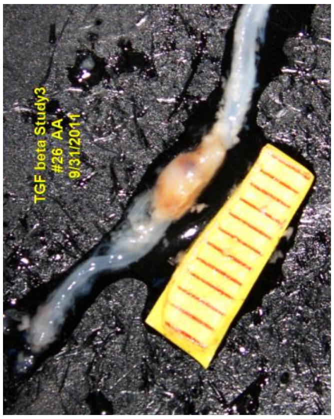

2.48 mm

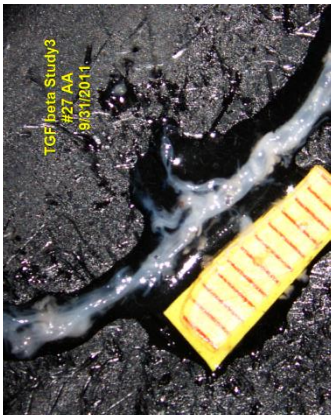

1.24 mm

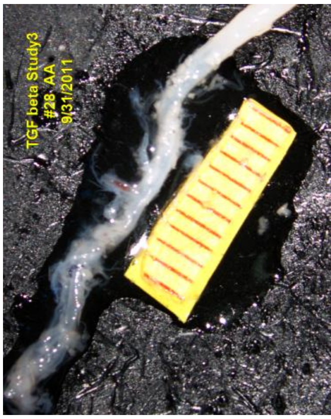

1.2 mm

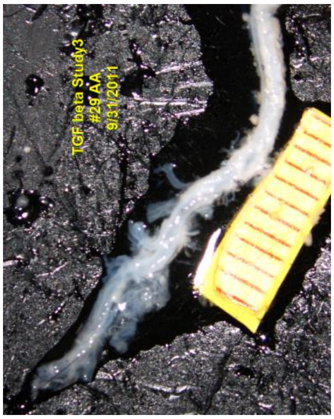

1.38 mm

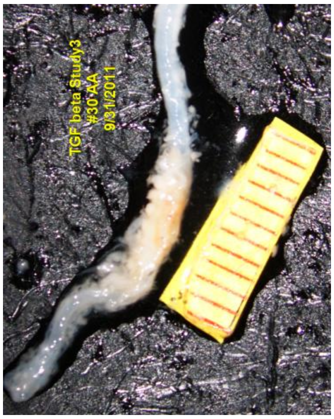

2.12 mm
