# Supplementary material for: TGF-β Neutralization Enhances AngII-Induced Aortic Rupture and Aneurysm in Both Thoracic and Abdominal Regions
Source: PLoS One. 2016 Apr 22;11(4):e0153811. doi: 10.1371/journal.pone.0153811 (PMC4841552; doi:10.1371/journal.pone.0153811)
Supplement: S10 Fig — Numbers below images are ascending aortic area measurements. (PDF) [file pone.0153811.s010.pdf]

S10 Fig

Study #2: Control, isotype-matched mouse IgG (5 mg/kg, 3 times/week) Saline-infused

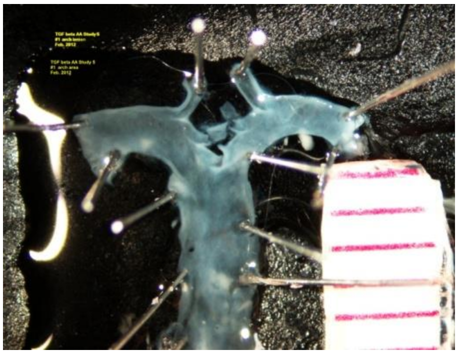

9.41 mm^2

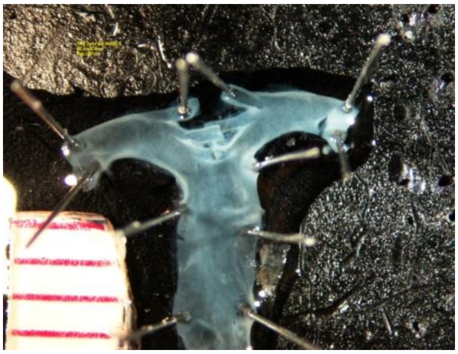

8.58 mm^2

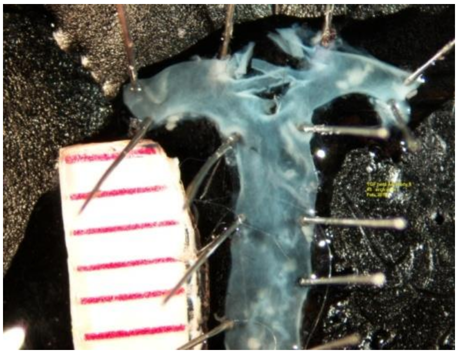

9.24 mm^2

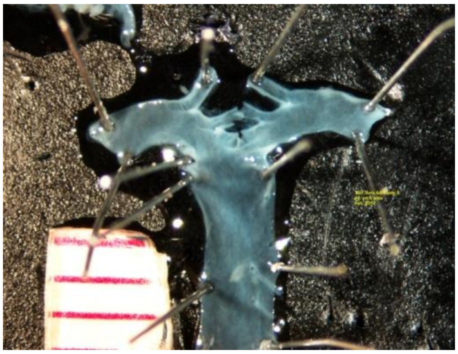

8.27 mm^2

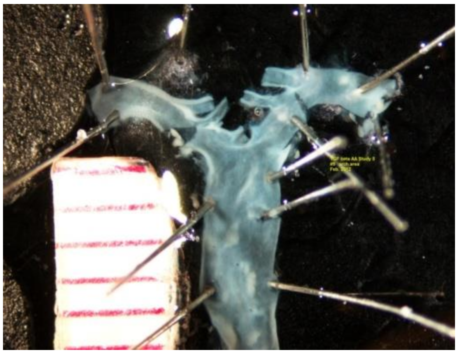

7.66 mm^2

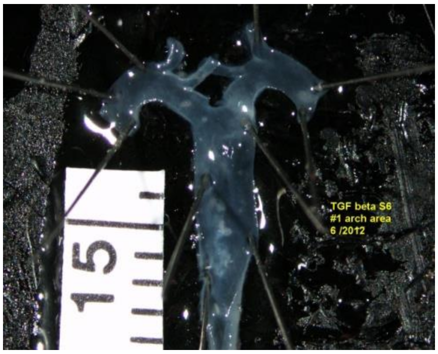

9.79 mm^2

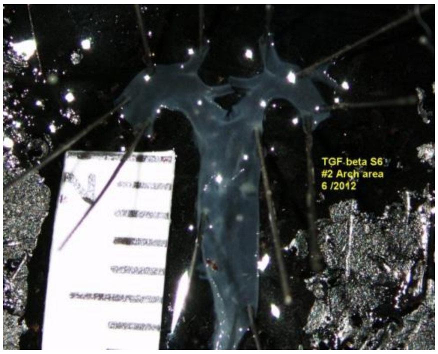

10.20 mm^2

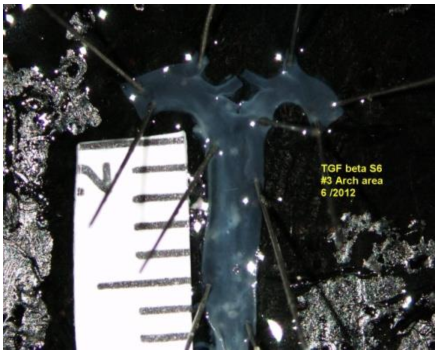

9.08 mm^2

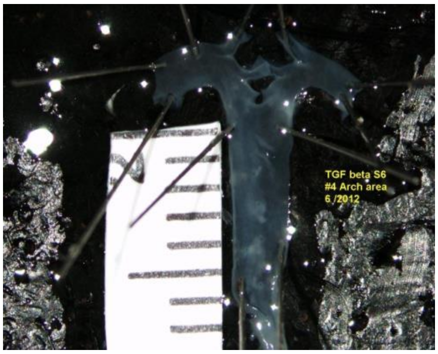

9.54 mm^2

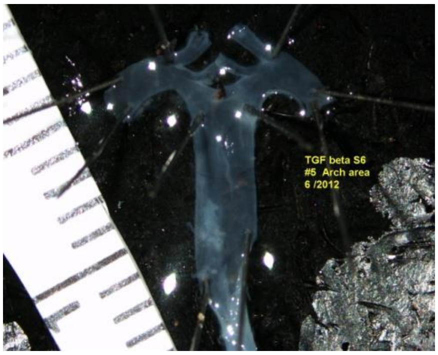

8.83 mm^2

Study #2: TGF-β mouse IgG
(5 mg/kg, 3 times/week)
Saline-infused

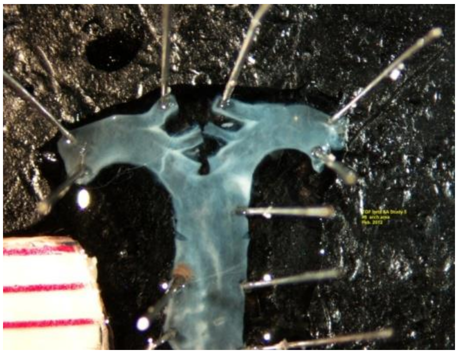

9.12 mm²

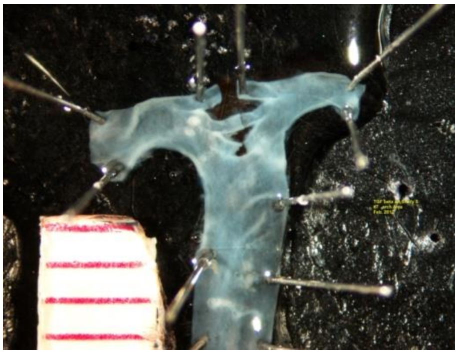

9.55 mm²

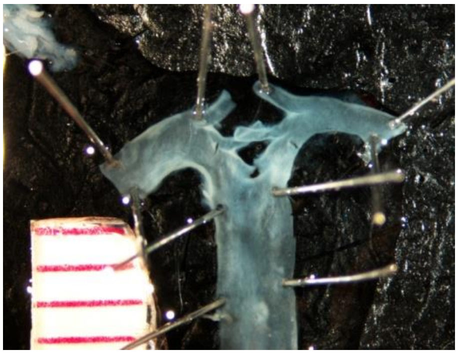

9.38 mm²

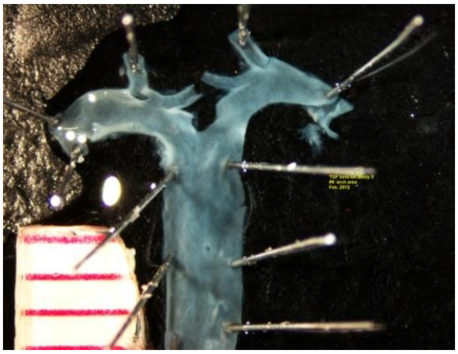

8.54 mm²

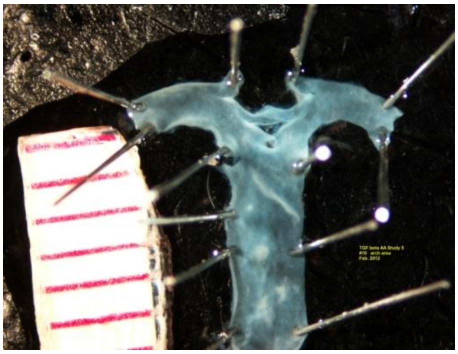

7.65 mm²

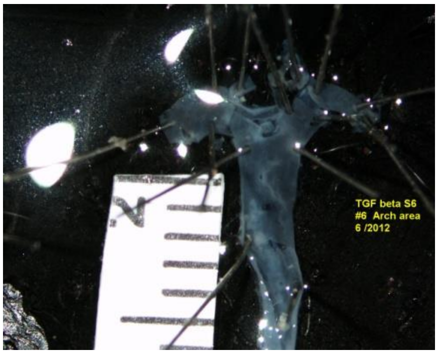

8.62 mm²

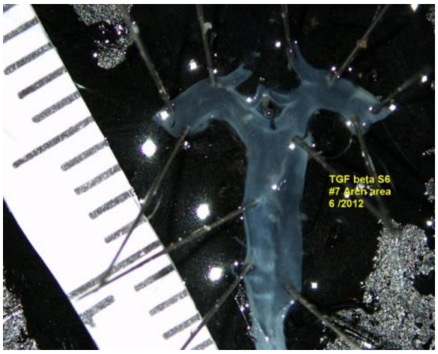

9.40 mm²

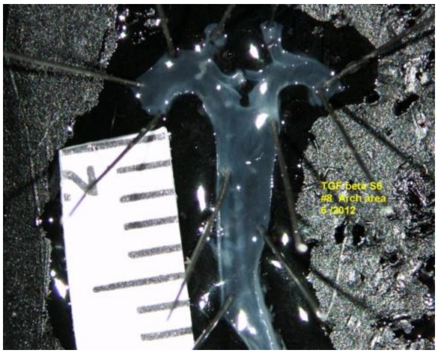

8.93 mm²

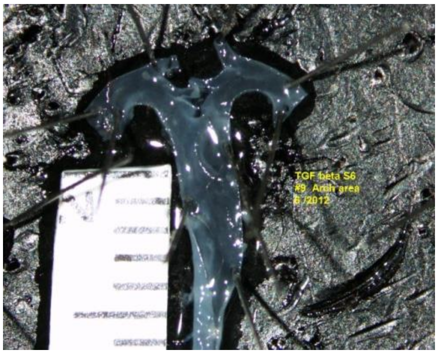

9.19 mm²

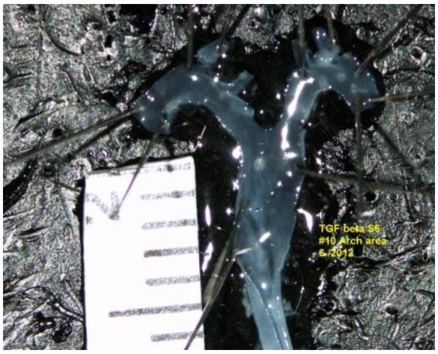

10.80 mm²

Study #2: Control, isotype-matched mouse IgG
(5 mg/kg, 3 times/week)
AngII-infused (1,000 ng/kg/min)

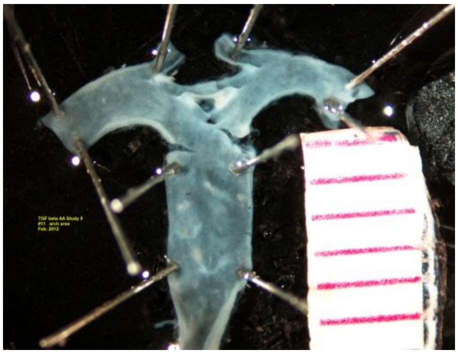
11.1 mm^2

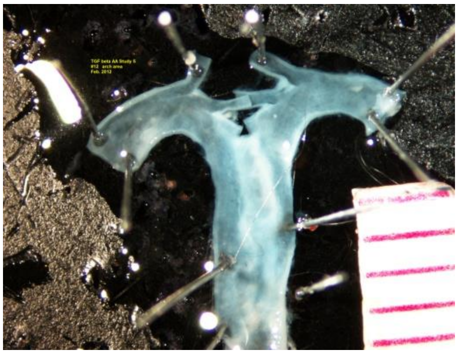
10.5 mm^2

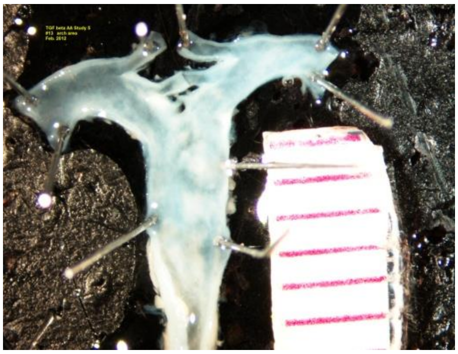
11.0 mm^2

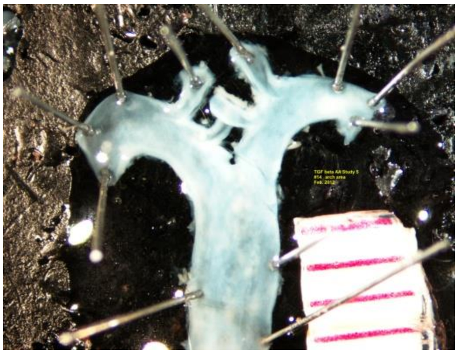
11.5 mm^2

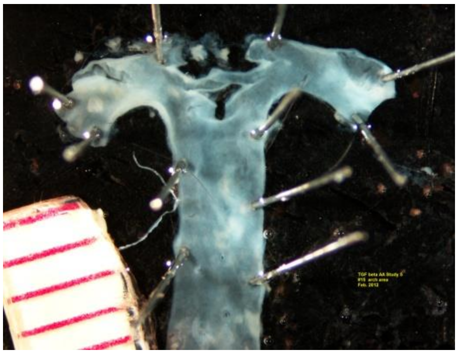
12.4 mm^2

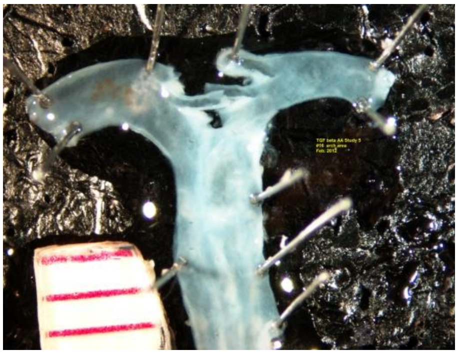
13.4 mm^2

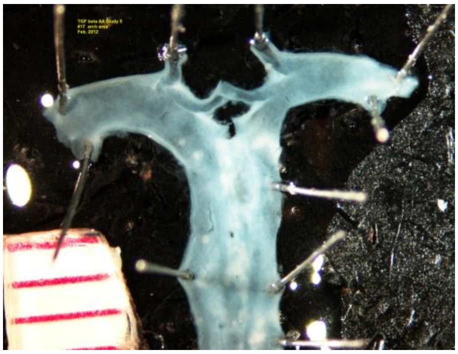
13.4 mm^2

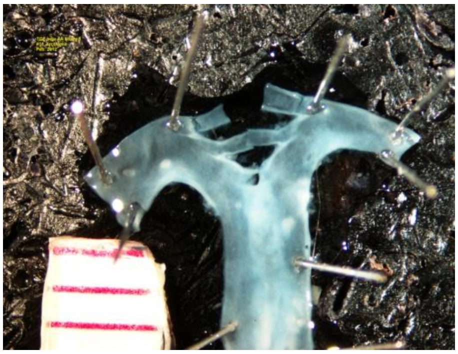
12.0 mm^2

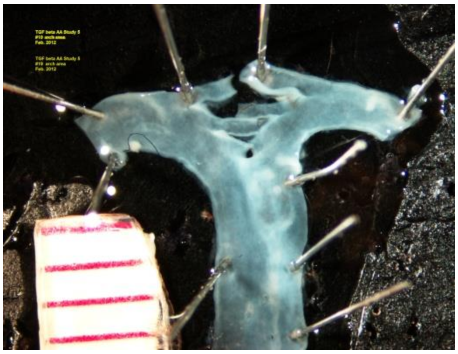
11.5 mm^2

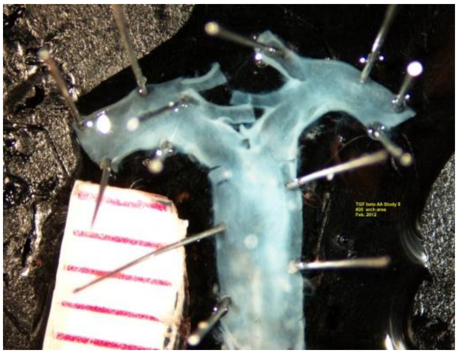
11.5 mm^2

Study #2: Control, isotype-matched IgG
(5 mg/kg, 3 times/week)
AngII-infused (1,000 ng/kg/min)

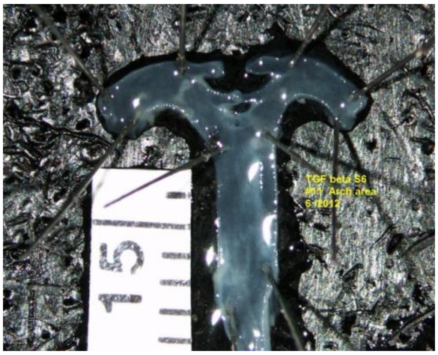

12.6 mm^2

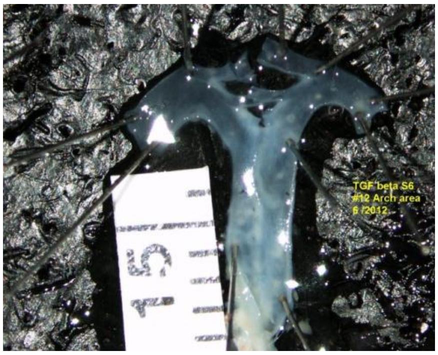

12.2 mm^2

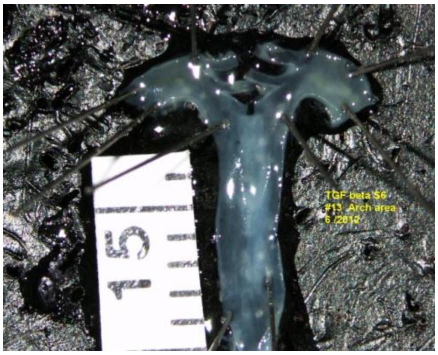

12.0 mm^2

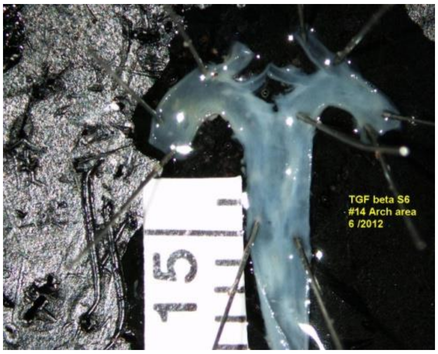

12.7 mm^2

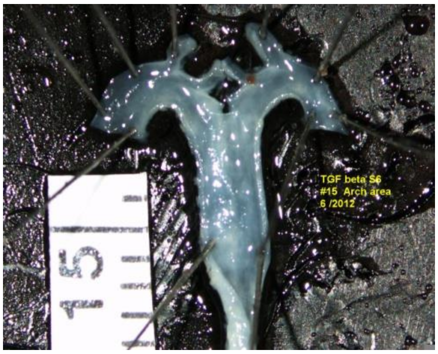

12.2 mm^2

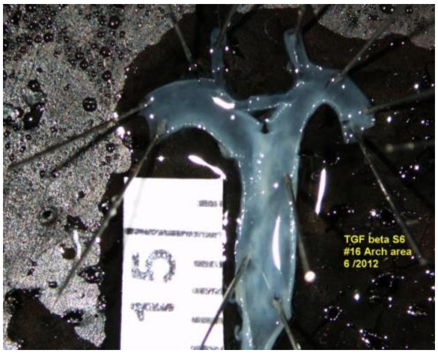

10.6 mm^2

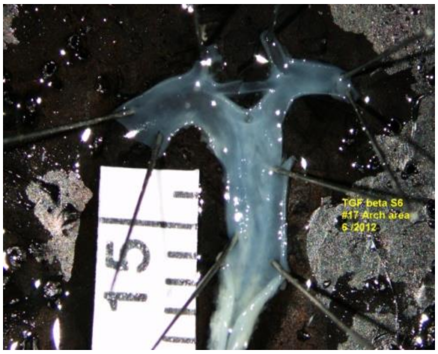

10.5 mm^2

#18: Died

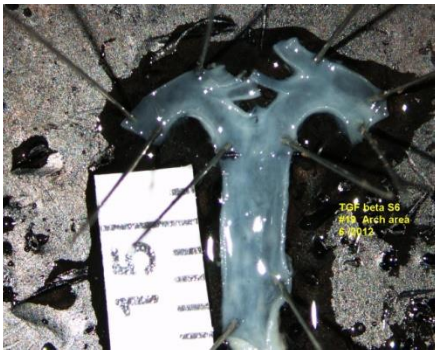

12.3 mm^2

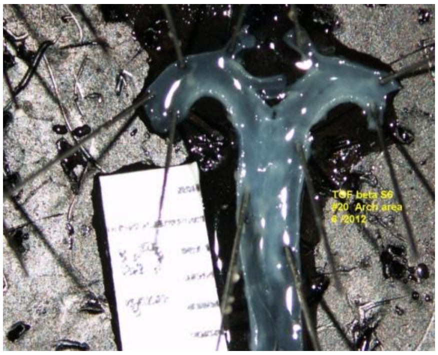

12.5 mm^2

Study #2: TGF-β mouse IgG
(5 mg/kg, 3 times/week)
AngII-infused (1,000 ng/kg/min)

#21a: Died

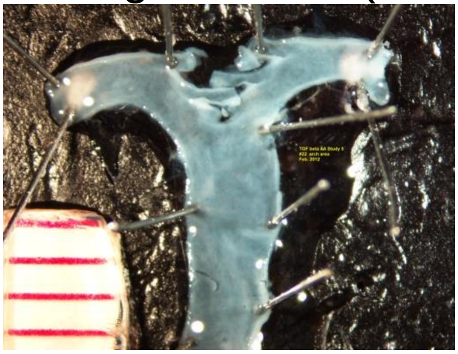

11.7 mm^2

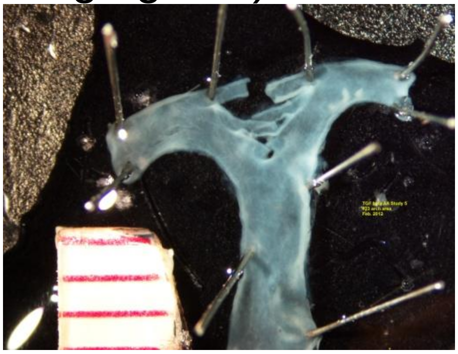

11.0 mm^2

#24a: Died

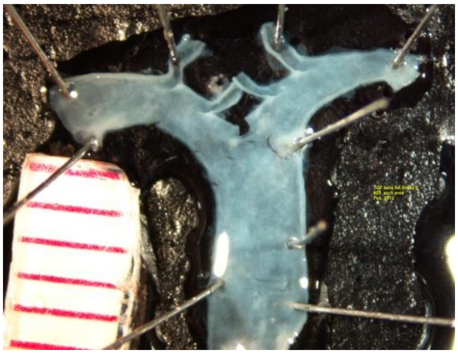

11.8 mm^2

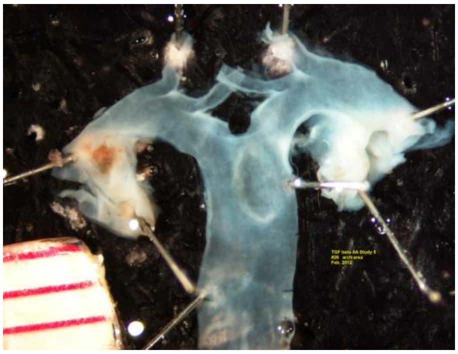

21.4 mm^2

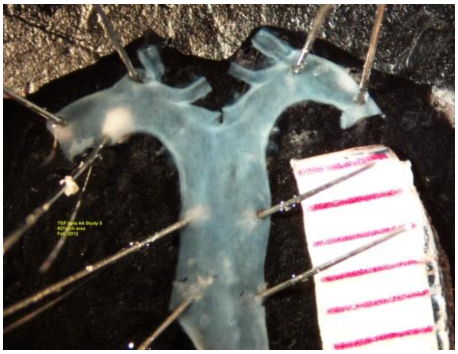

11.0 mm^2

#28a: Died

#29a: Died

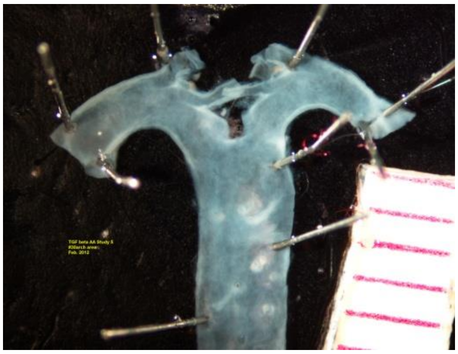

13.7 mm^2

Study #2: TGF-β mouse IgG
(5 mg/kg, 3 times/week)
AngII-infused (1,000 mg/kg/min)

#21b: Died

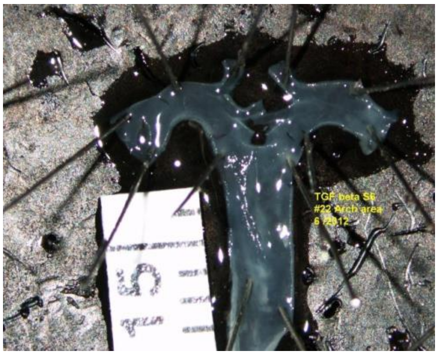

$16.0\ mm^2$

#23b: Died

#24b: Died

#25b: Died

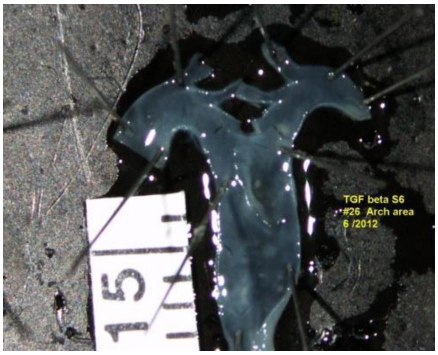

$13.3\ mm^2$

#27b: Died

#28b: Died

#29b: Died

#30b: Died
