# Supplementary material for: TGF-β Neutralization Enhances AngII-Induced Aortic Rupture and Aneurysm in Both Thoracic and Abdominal Regions
Source: PLoS One. 2016 Apr 22;11(4):e0153811. doi: 10.1371/journal.pone.0153811 (PMC4841552; doi:10.1371/journal.pone.0153811)
Supplement: S11 Fig — Numbers below images are suprarenal aortic diameter measurements. (PDF) [file pone.0153811.s011.pdf]

S11 Fig

# Study #2: Control, isotype-matched mouse IgG (5 mg/kg, 3 times/week) Saline-infused

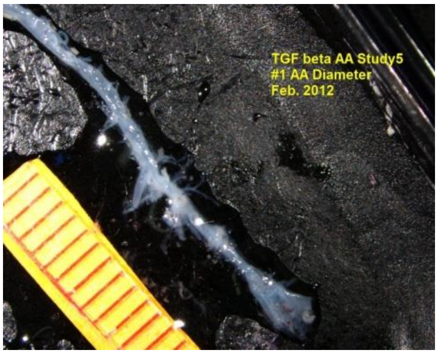


0.78 mm

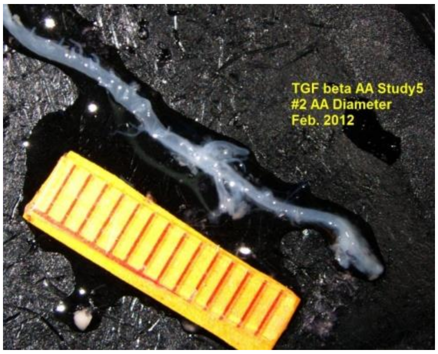


0.82 mm

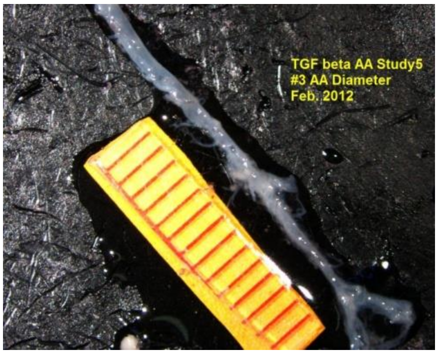


0.72 mm

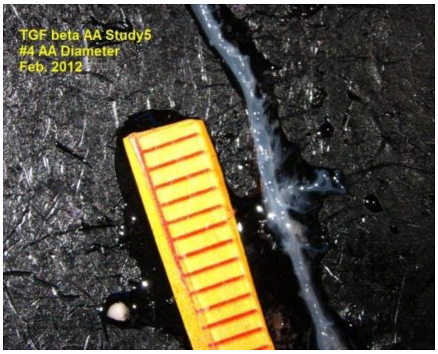


0.73 mm

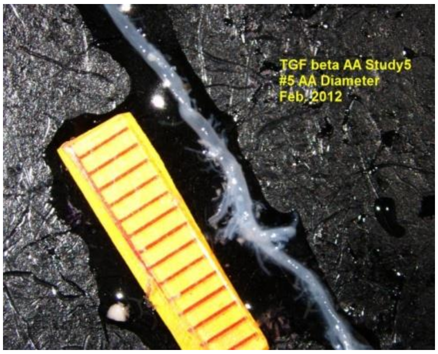


0.75 mm

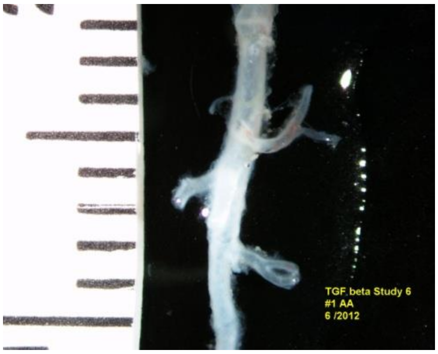


0.81 mm

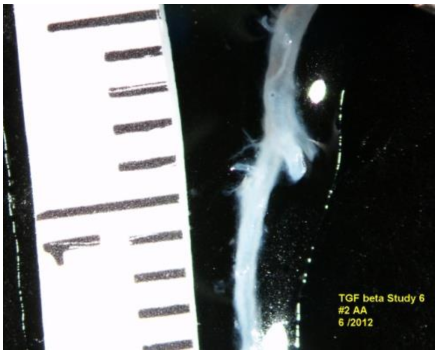


0.78 mm

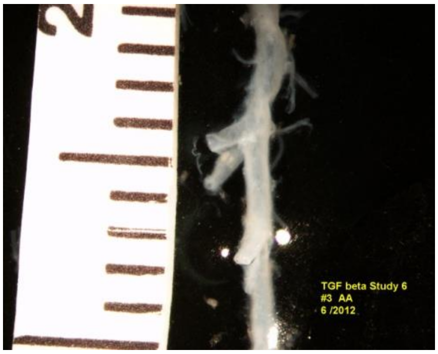


0.81 mm

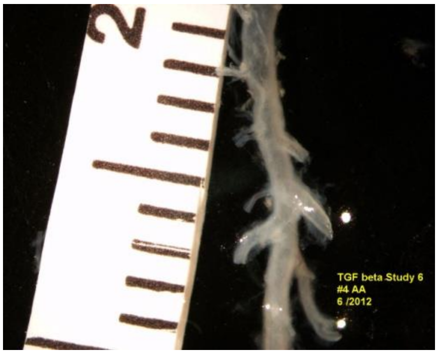


0.74 mm

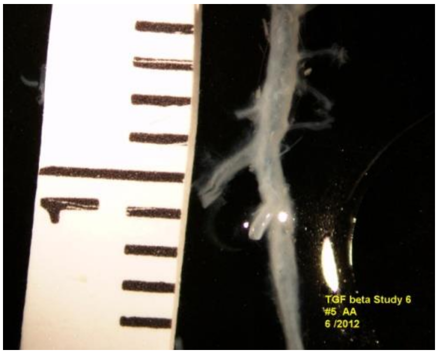


0.84 mm

Study #2: TGF-β mouse IgG
(5 mg/kg, 3 times/week)
Saline-infused

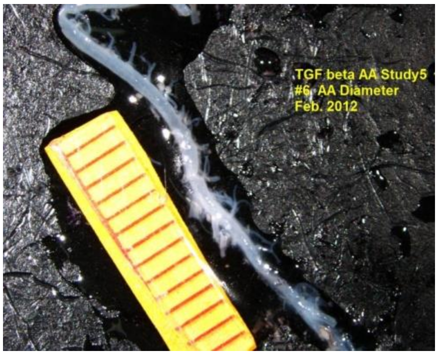


0.80 mm

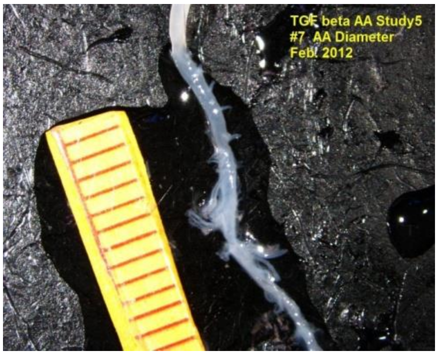


0.88 mm

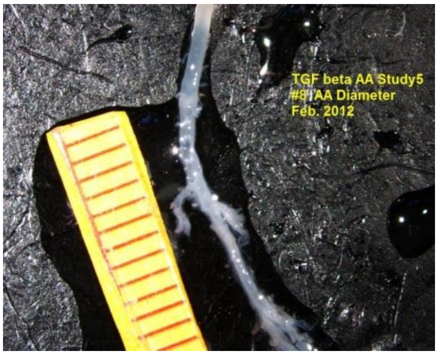


0.84 mm

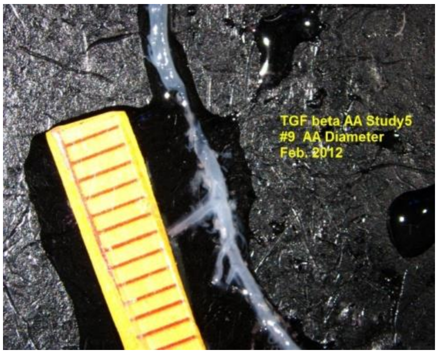


0.82 mm

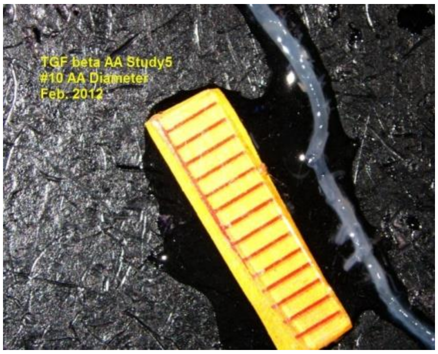


0.80 mm

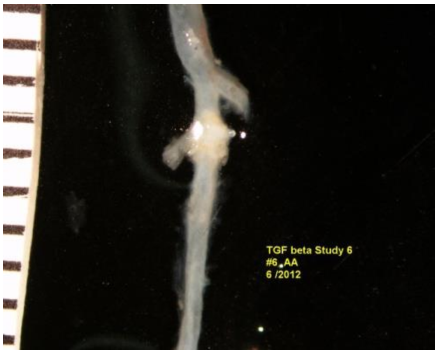


1.12 mm

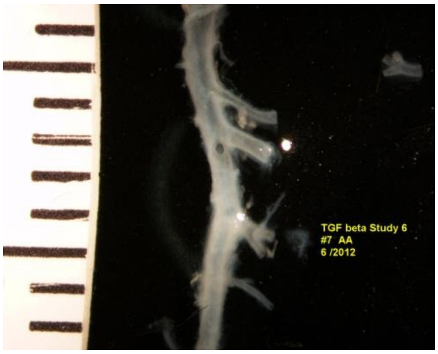


0.83 mm

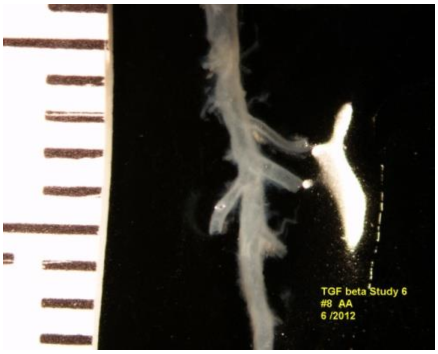


0.75 mm

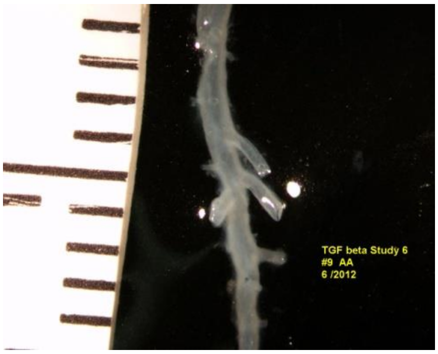


1.02 mm

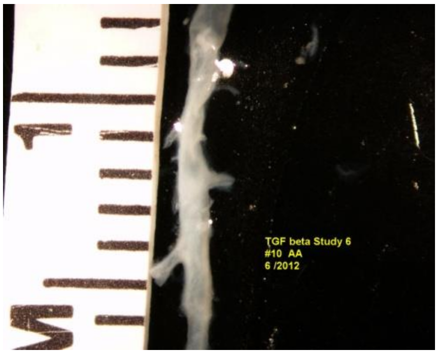


0.73 mm

Study #2: Control, isotype-matched mouse IgG
(5 mg/kg; 3 times/week)
AngII-infused (1,000 ng/kg/min

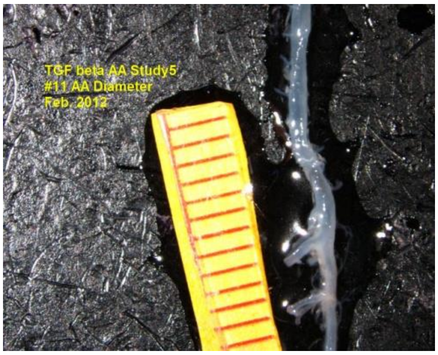

0.98 mm

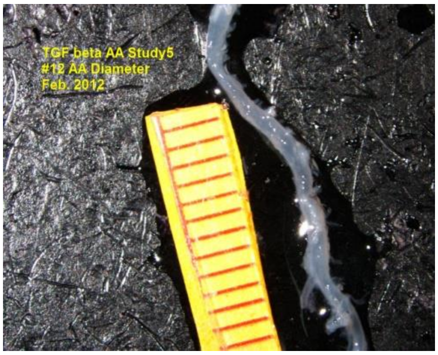

1.08 mm

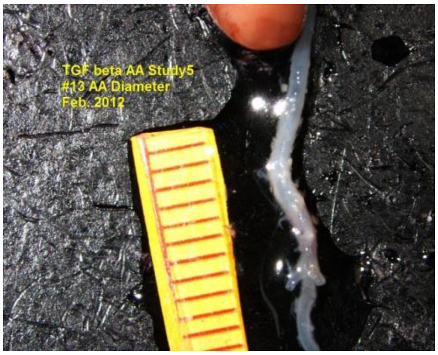

1.19 mm

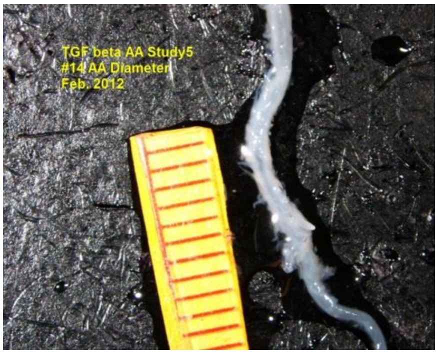

1.06 mm

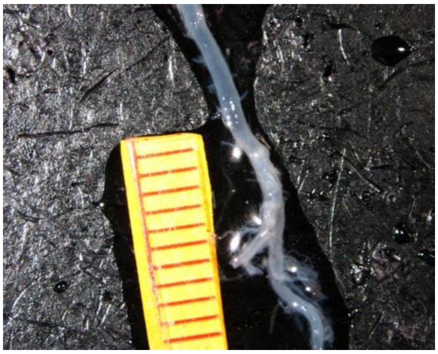
1.13 mm

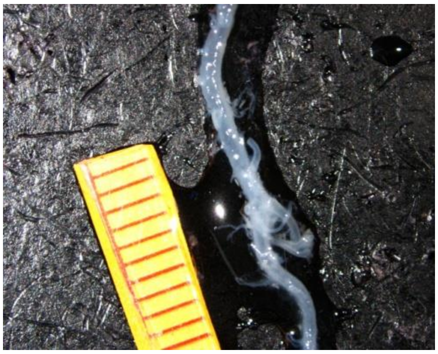
1.10 mm

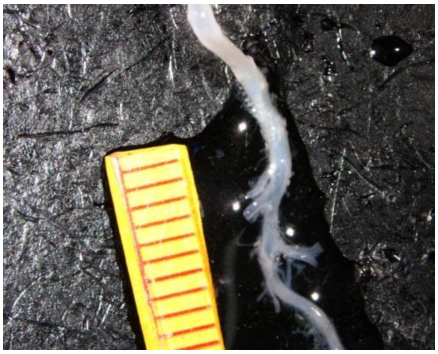
1.16 mm

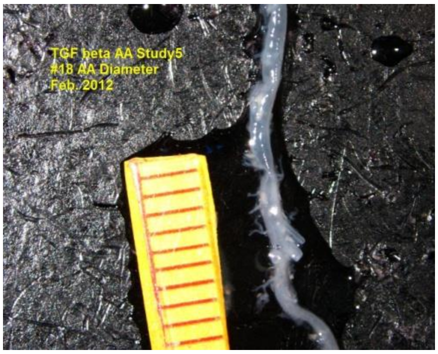

1.11 mm

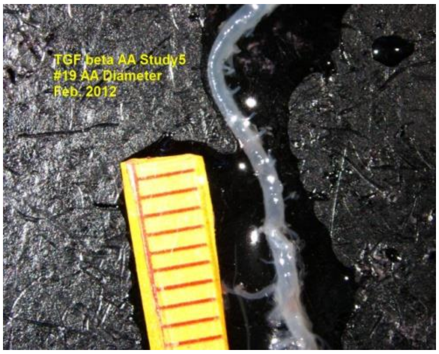

1.15 mm

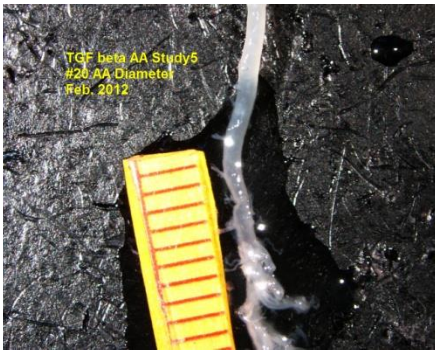

0.99 mm

Study #2: Control, isotype-matched IgG
(5 mg/kg, 3 times/week)
AngII (1,000 mg/kg/min)

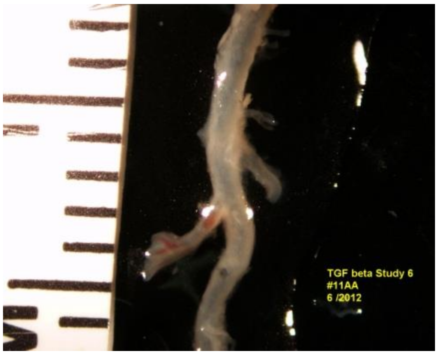


0.94 mm

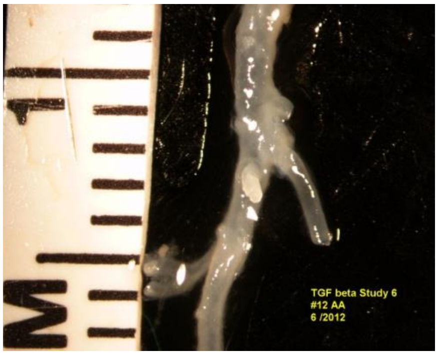


1.06 mm

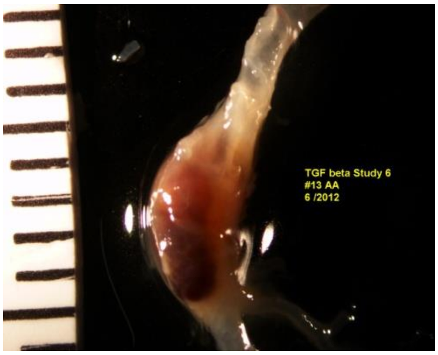


2.63 mm

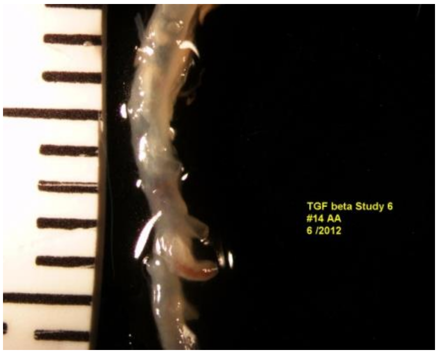


1.05 mm

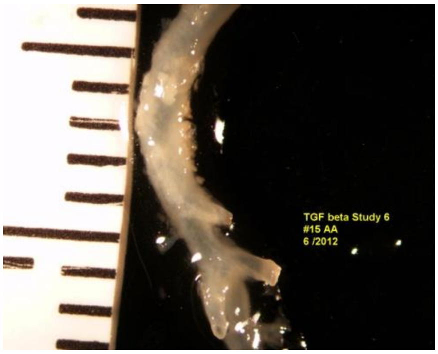


1.26 mm

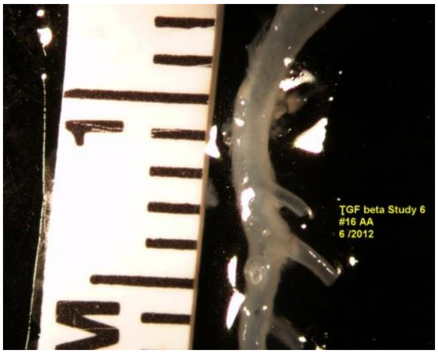


1.04 mm

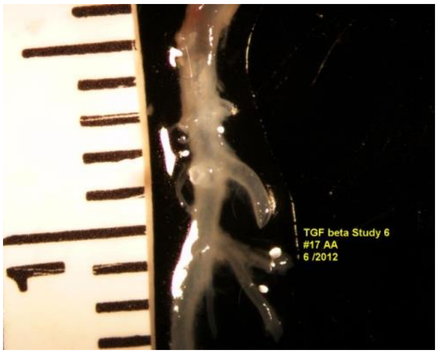


0.98 mm

#18: Died

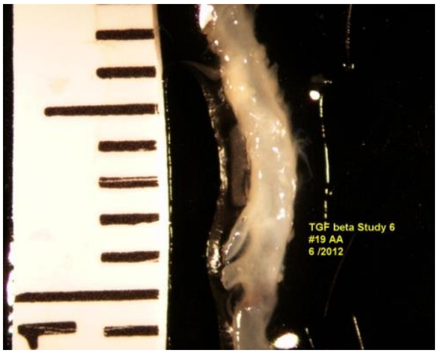


1.23 mm

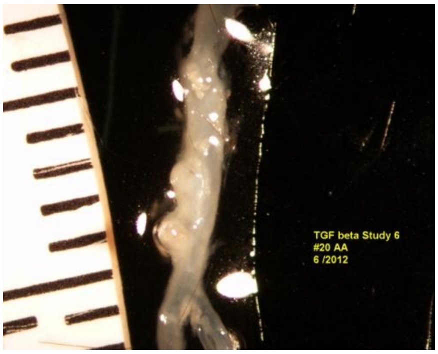


1.37 mm

Study #2: TGF-β mouse IgG
(5 mg/kg, 3 times/week)
AngII-infused (1,000 ng/kg/min)

#21a: Died

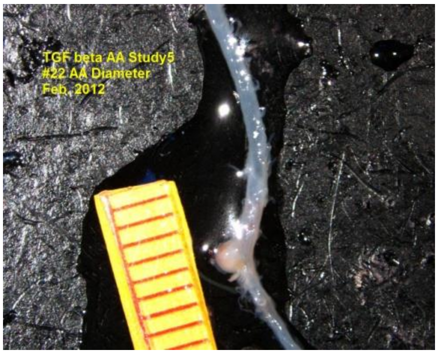


2.04 mm

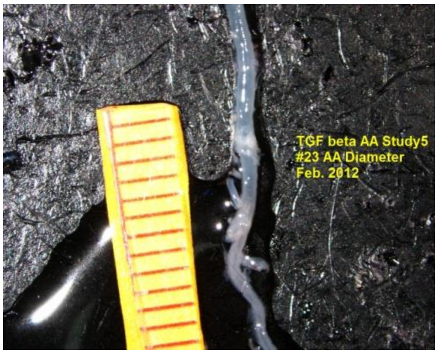


0.82 mm

#24a: Died

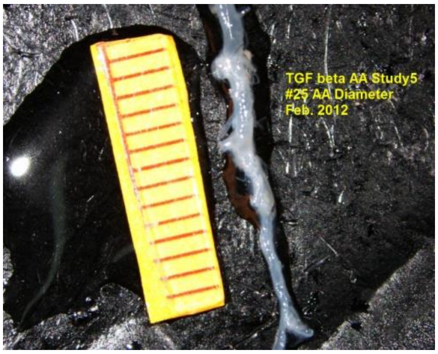


0.97 mm

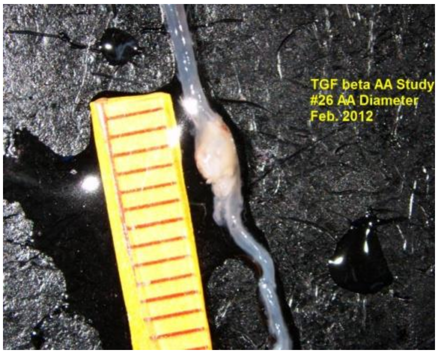


2.26 mm

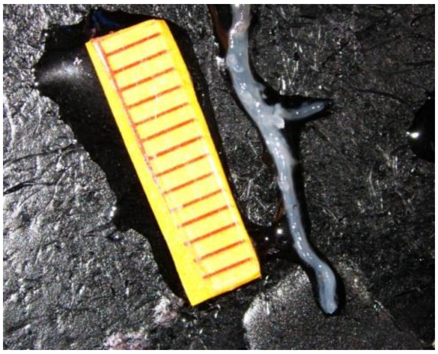

1.05 mm

#28a: Died

#29a: Died

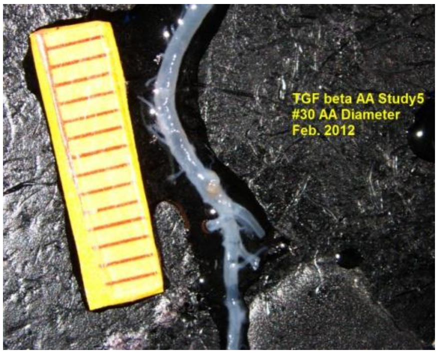


1.35 mm

Study #2: TGF-β mouse IgG
(5 mg/kg, 3 times/week)
AngII –infused (1,000 ng/kg/min)

#21b: Died

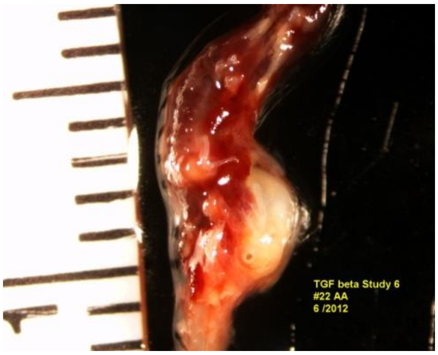


3.63 mm

#23b: Died

#24b: Died

#25b: Died

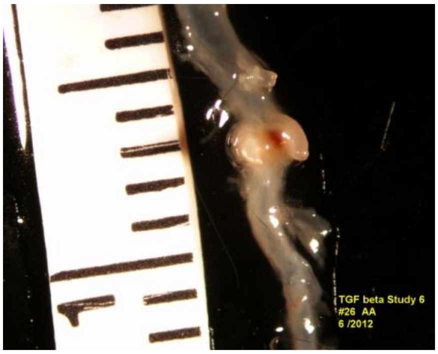


1.60 mm

#27b: Died

#28b: Died

#29b: Died

#30b: Died
