# Supplementary material for: TGF-β Neutralization Enhances AngII-Induced Aortic Rupture and Aneurysm in Both Thoracic and Abdominal Regions
Source: PLoS One. 2016 Apr 22;11(4):e0153811. doi: 10.1371/journal.pone.0153811 (PMC4841552; doi:10.1371/journal.pone.0153811)
Supplement: S12 Fig — Teal lines indicate the outer curvature and diameter measurements of ascending aortas. (PDF) [file pone.0153811.s012.pdf]

S12 Fig

Control, isotype-matched mouse IgG
(5 mg/kg, 3 times/week)
Saline-infused
Day 0

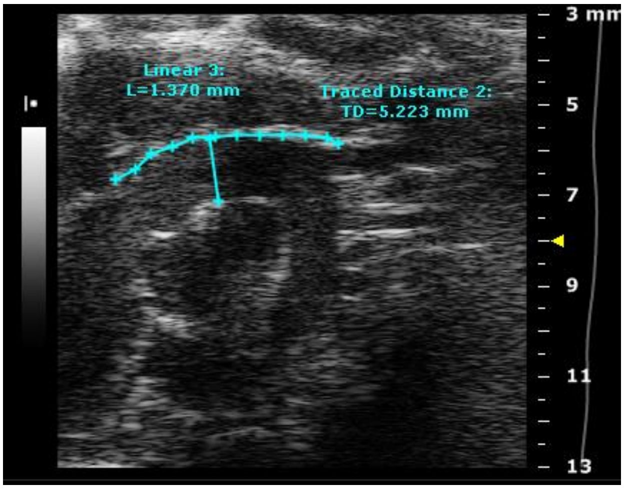

1.37 mm

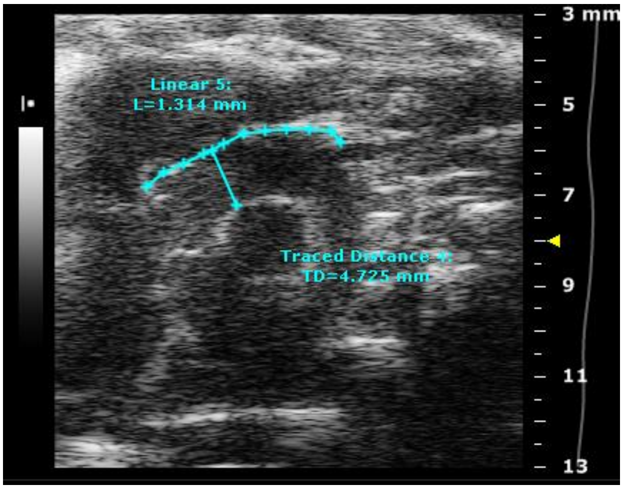

1.31 mm

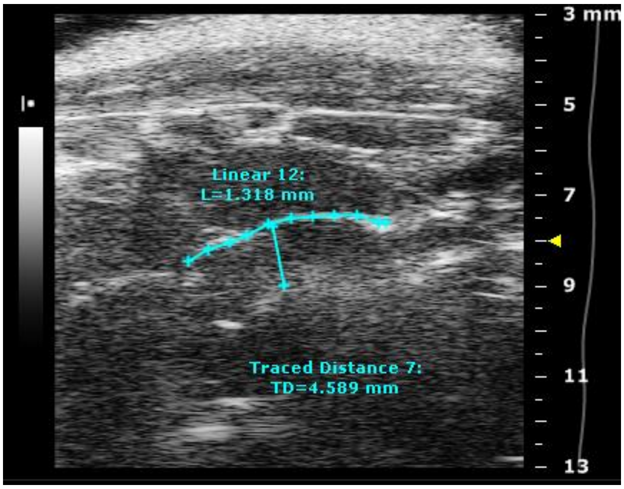

1.32 mm

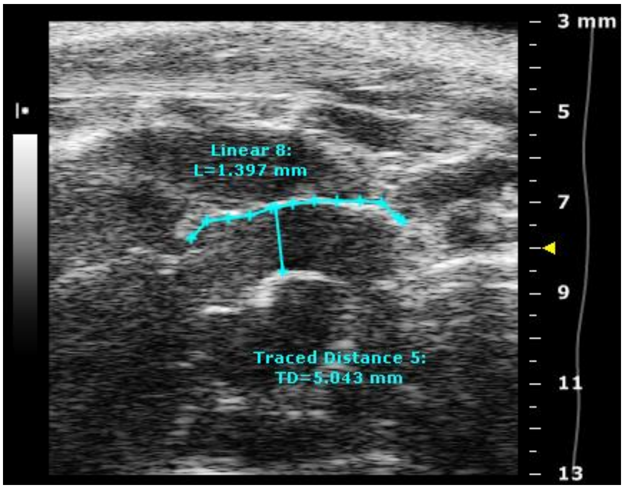

1.40 mm

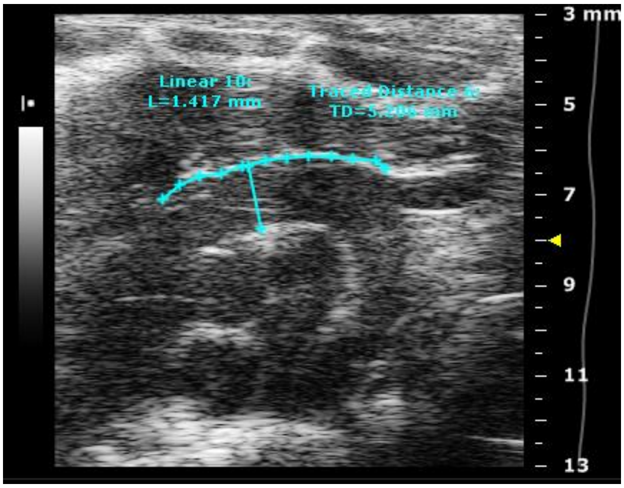

1.42 mm

TGF-β mouse IgG
(5 mg/kg, 3 times/week)
Saline-infused
Day 0

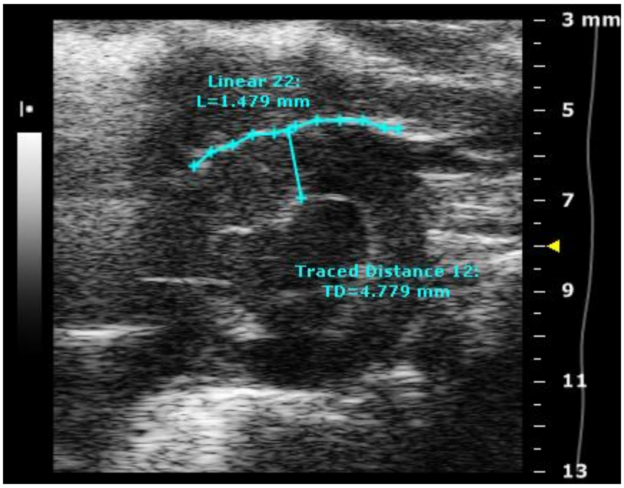

1.48 mm

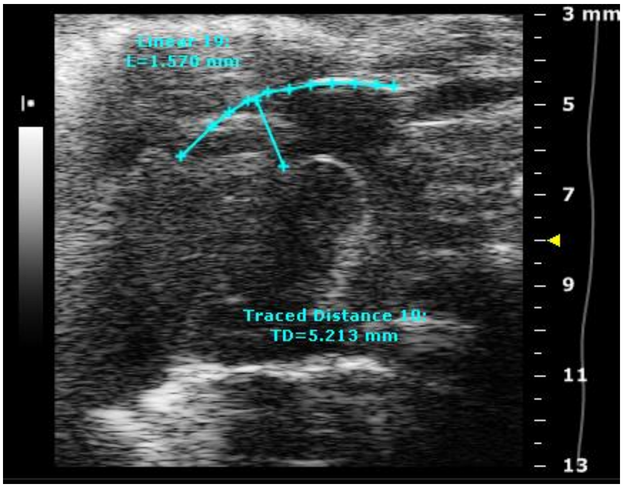

1.57 mm

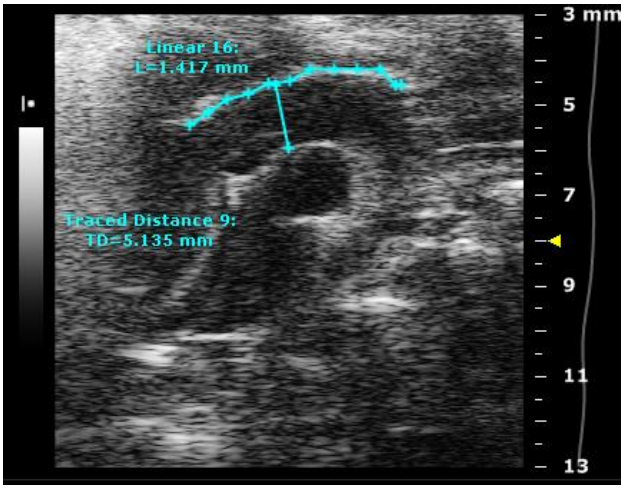

1.42 mm

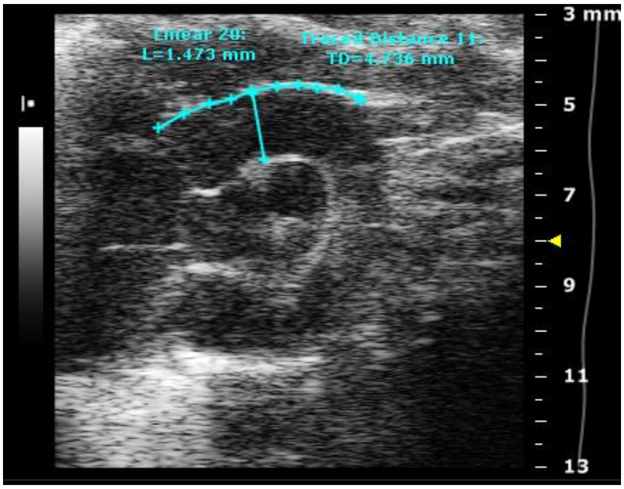

1.47 mm

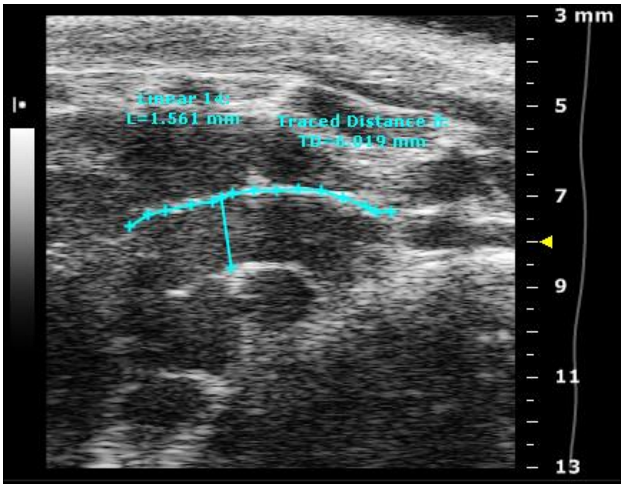

1.56 mm

Control, isotype-matched mouse IgG
(5 mg/kg, 3 times/week)
AngII-infused (1,000 ng/kg/min)
Day 0

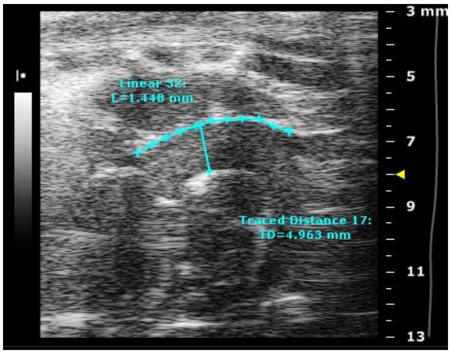

1.45 mm

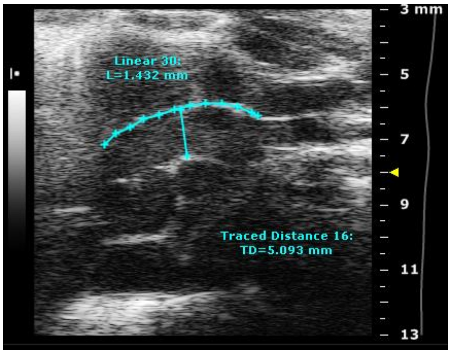

1.43 mm

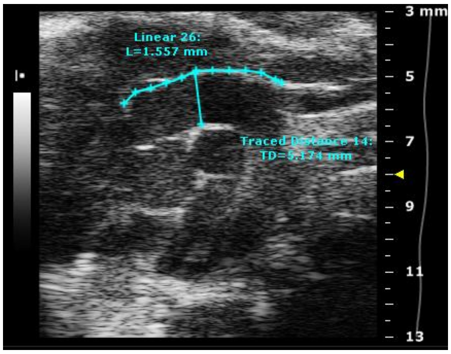

1.56 mm

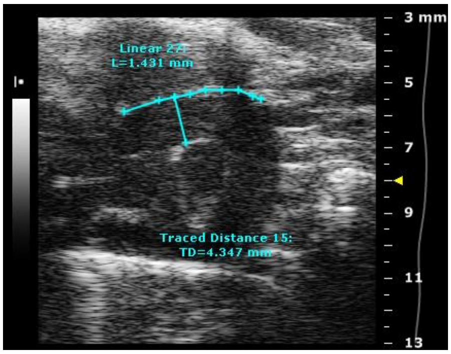

1.43 mm

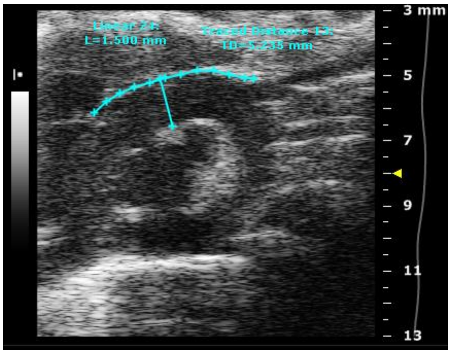

1.50 mm

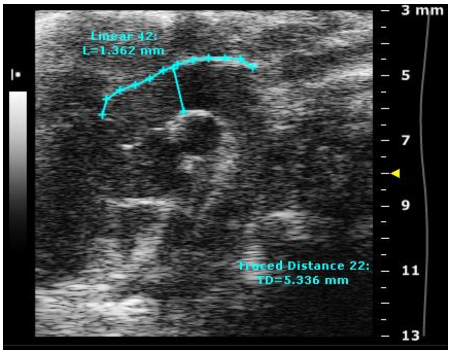

1.36 mm

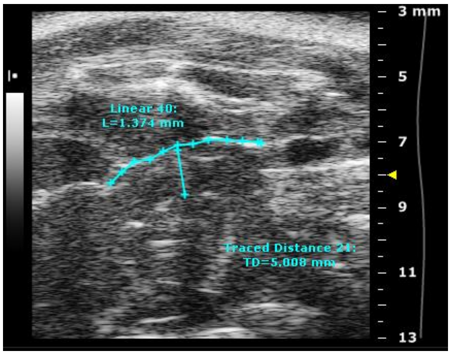

1.37 mm

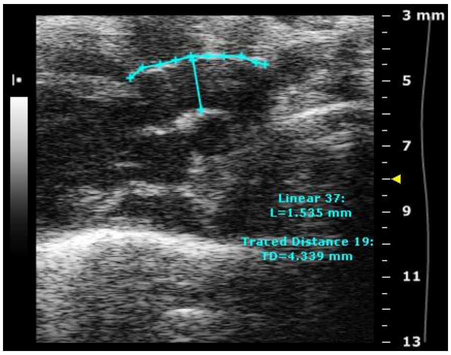

1.53 mm

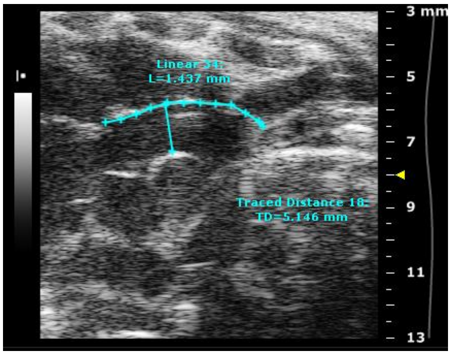

1.43 mm

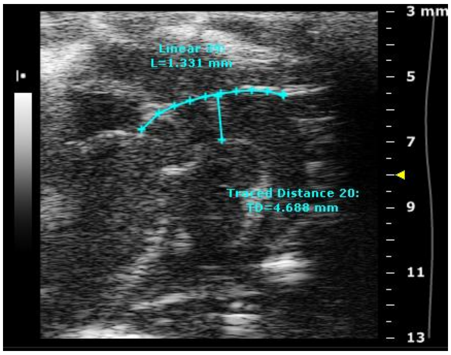

1.33 mm

TGF-β mouse IgG
(5 mg/kg, 3 times/week)
AngII-infused (1,000 ng/kg/min)
Day 0

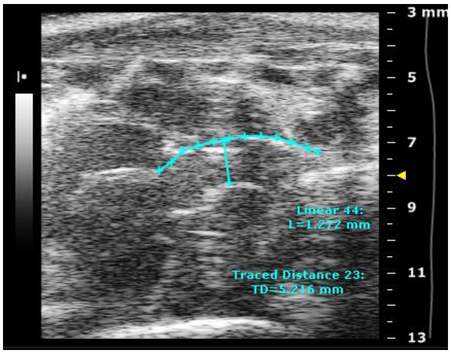

1.27 mm

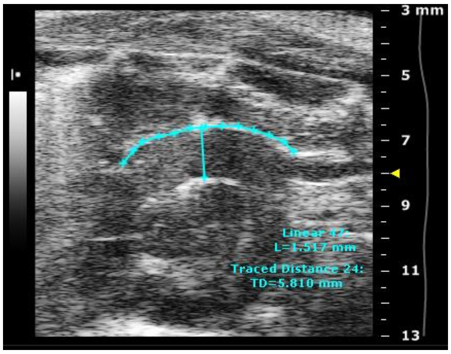

1.52 mm

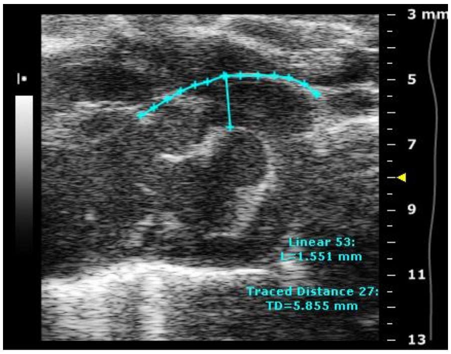

1.55 mm

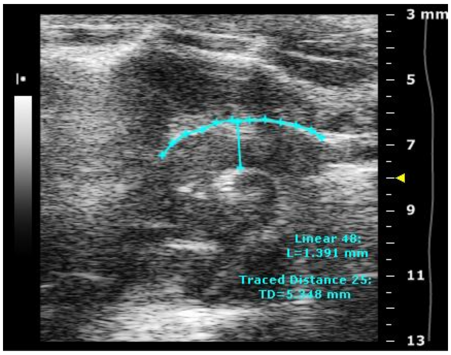

1.39 mm

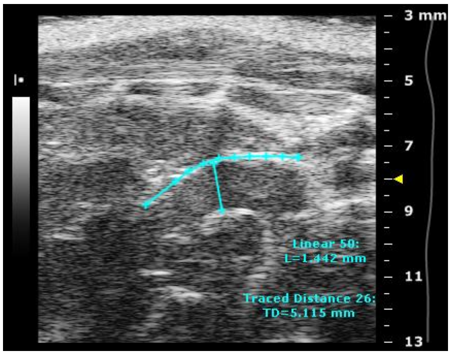

1.44 mm

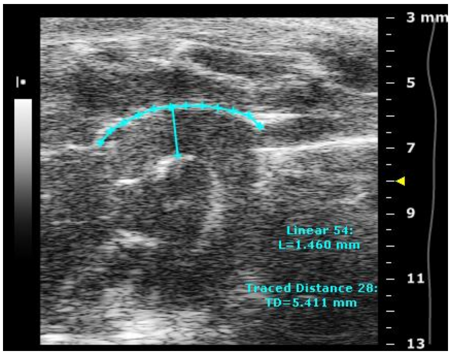

1.46 mm

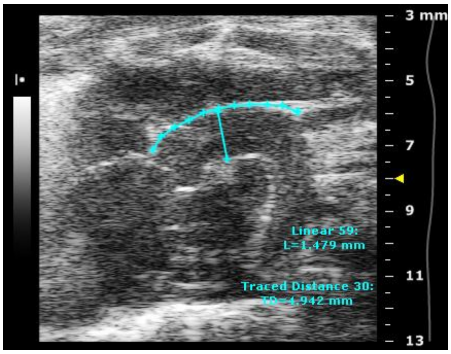

1.47 mm

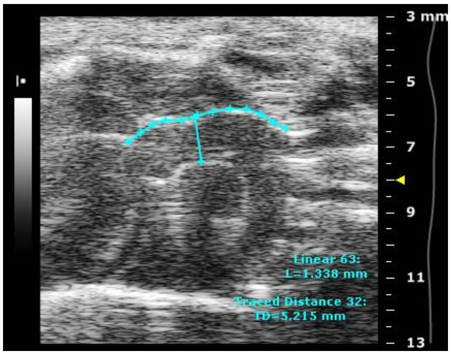

1.33 mm

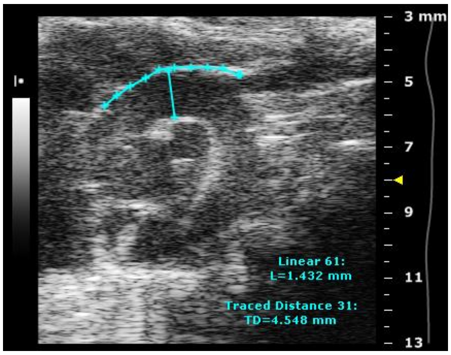

1.43 mm

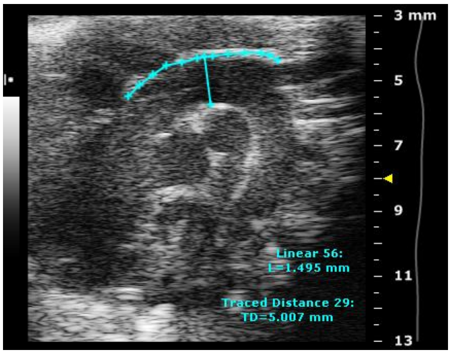

1.50 mm

Control, isotype-matched mouse IgG
(5 mg/kg, 3 times/week)
Saline-infused
Day 4

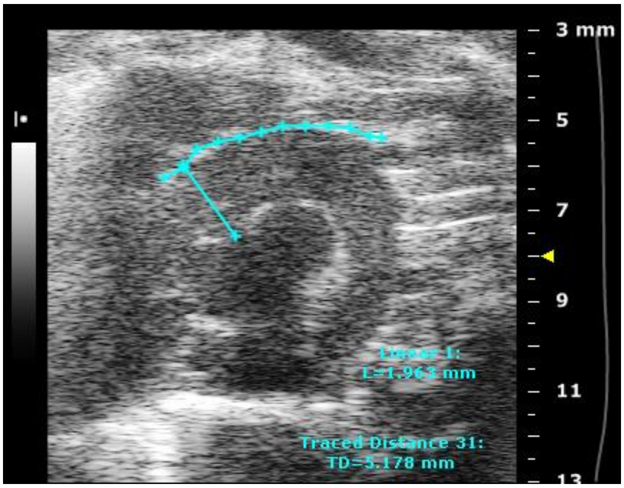

1.96 mm

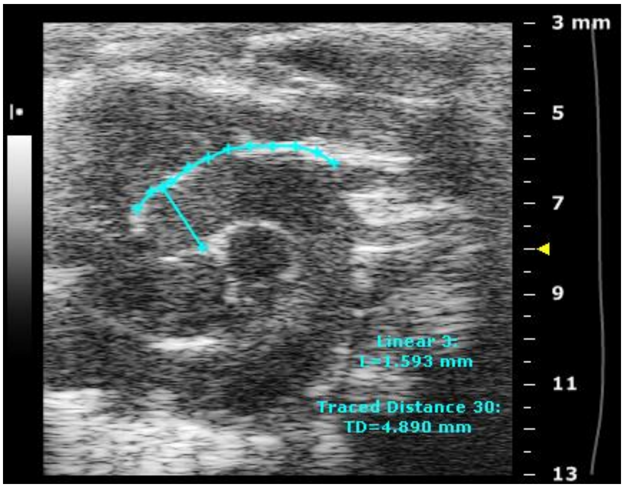

1.59 mm

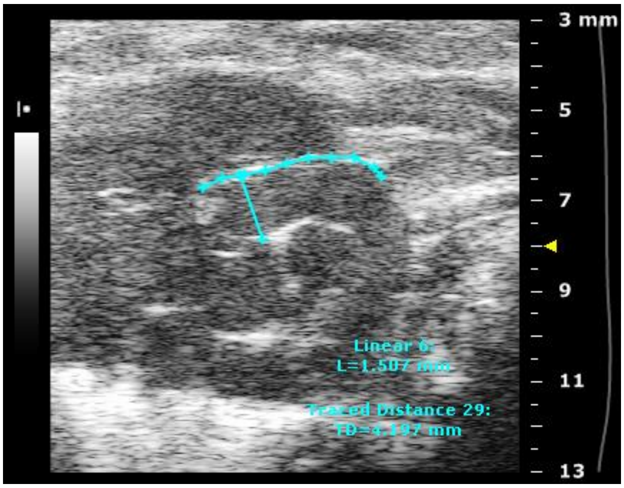

1.51 mm

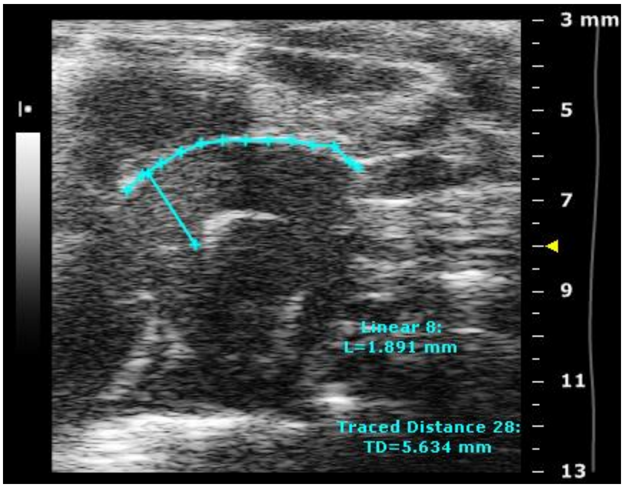

1.89 mm

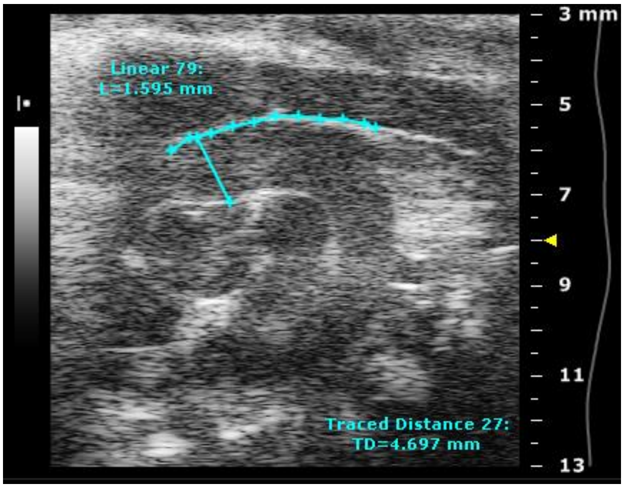

1.50 mm

TGF-β mouse IgG
(5 mg/kg, 3 times/week)
Saline-infused
Day 4

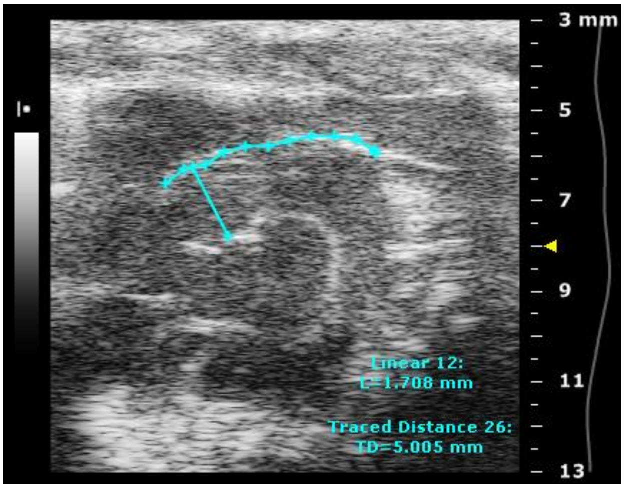

1.71 mm

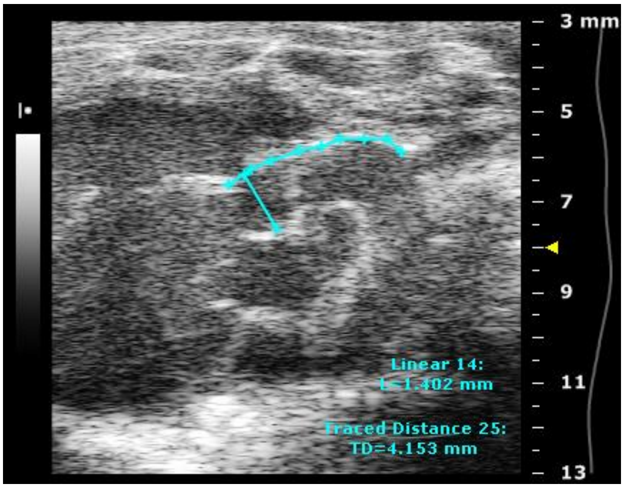

1.40 mm

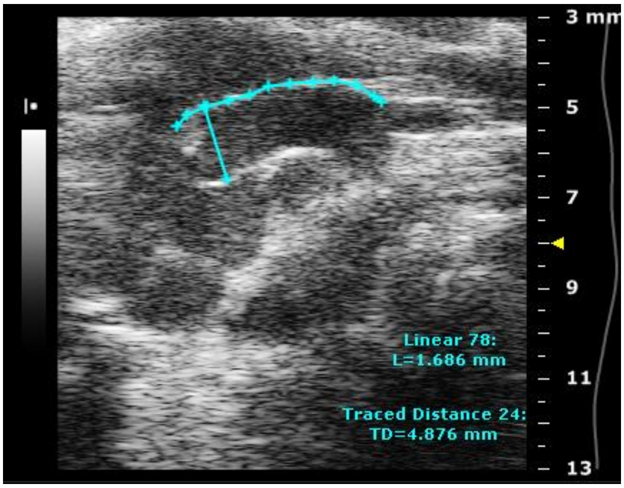

1.69 mm

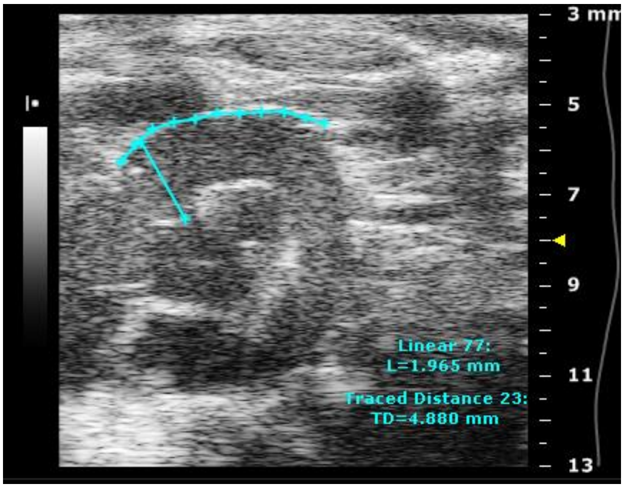

1.97 mm

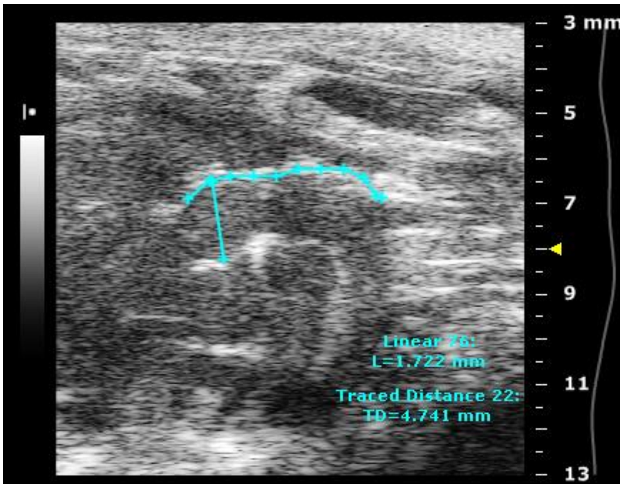

1.72 mm

Control, isotype-matched mouse IgG
(5 mg/kg, 3 times/week)
AngII-infused (1,000 ng/kg/min)
Day 4

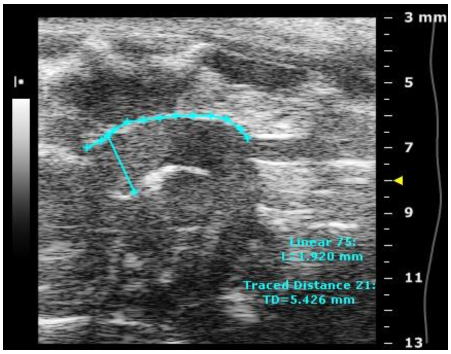

1.92 mm

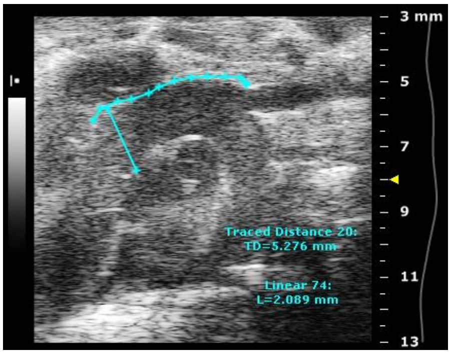

2.09 mm

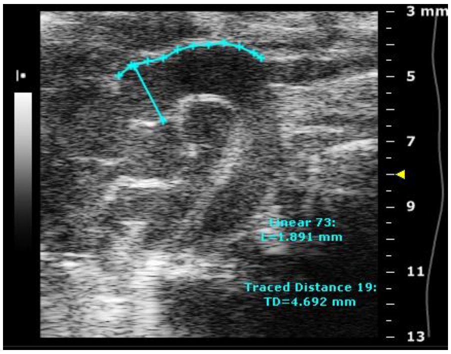

1.89 mm

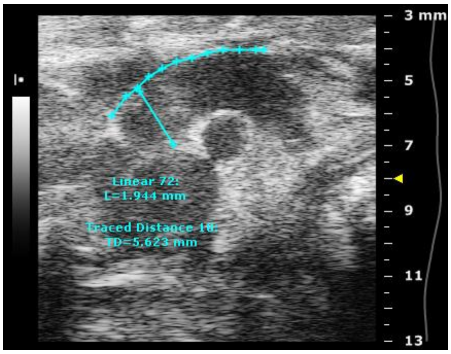

1.99 mm

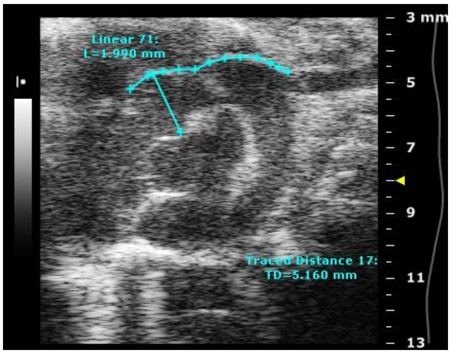

1.99 mm

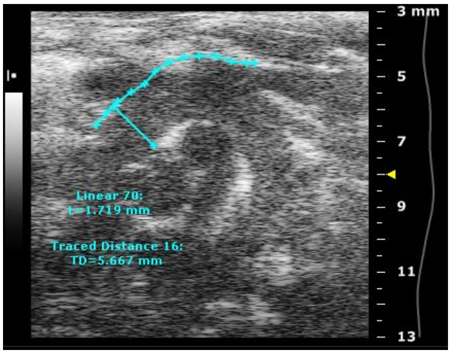

1.72 mm

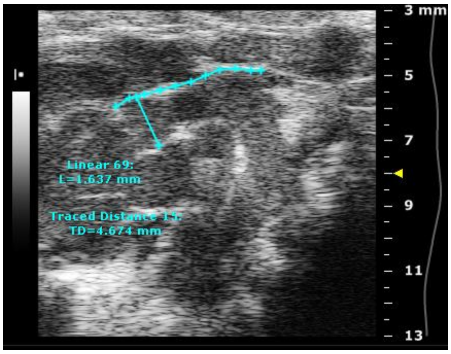

1.64 mm

#18b:Died

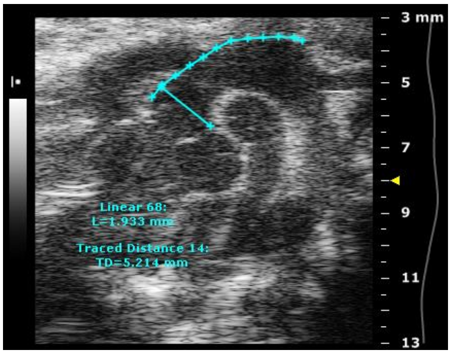

1.93 mm

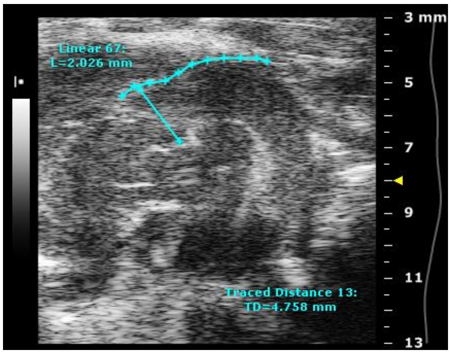

2.02 mm

TGF-β mouse IgG
(5 mg/kg, 3 times/week)
AngII-infused (1,000 ng/kg/min)
Day 4

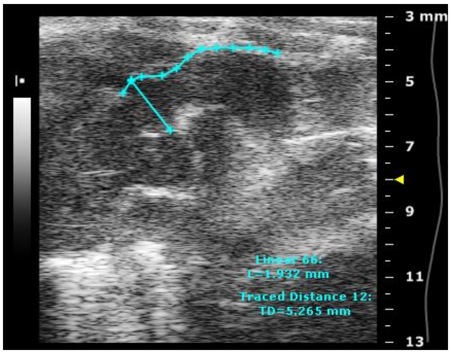

1.93 mm

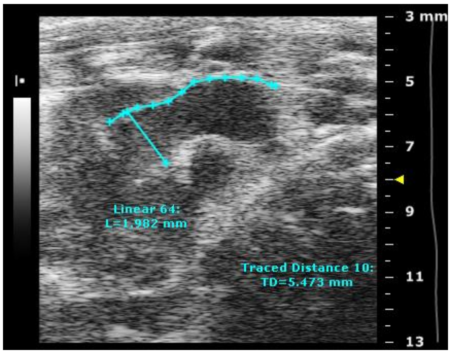

1.98 mm

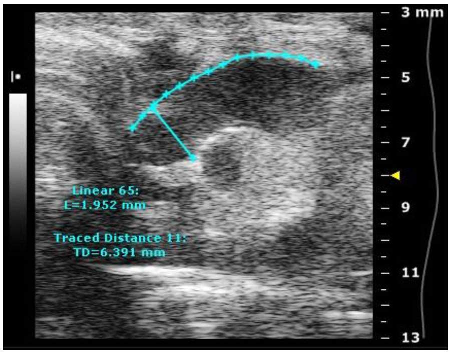

1.95 mm

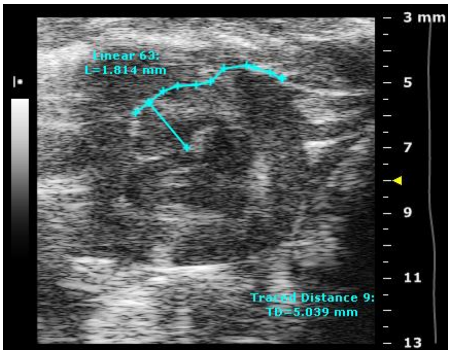

1.81 mm

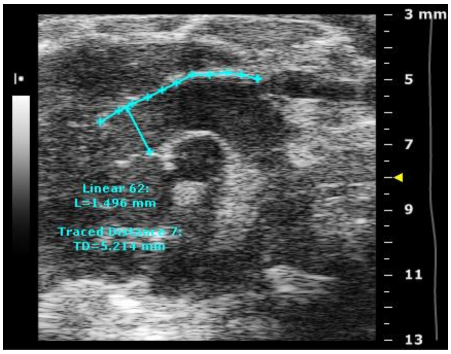

1.50 mm

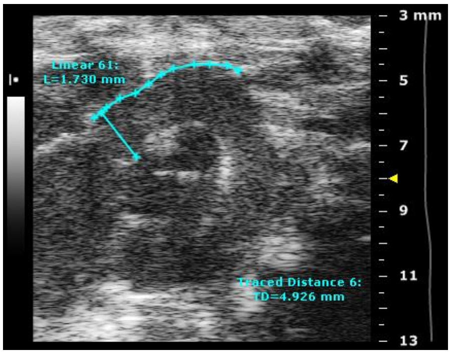

1.73 mm

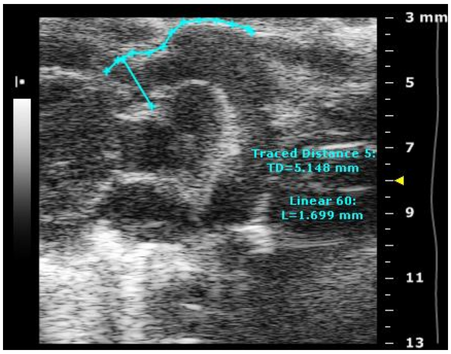

1.70 mm

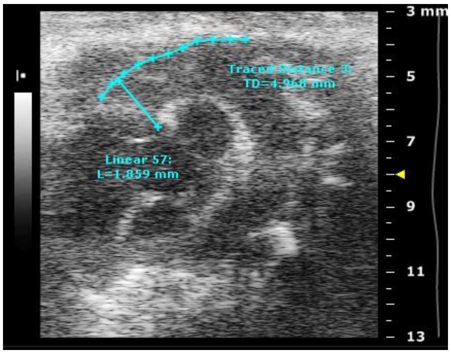

1.86 mm

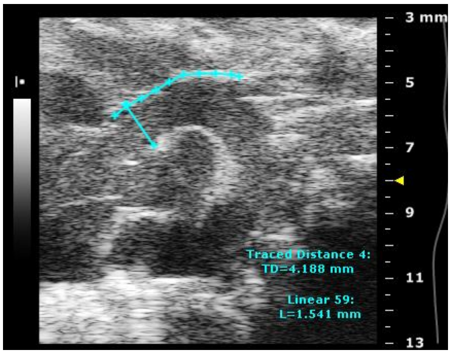

1.54 mm

#30:Died
