# Supplementary material for: TGF-β Neutralization Enhances AngII-Induced Aortic Rupture and Aneurysm in Both Thoracic and Abdominal Regions
Source: PLoS One. 2016 Apr 22;11(4):e0153811. doi: 10.1371/journal.pone.0153811 (PMC4841552; doi:10.1371/journal.pone.0153811)
Supplement: S13 Fig — Teal lines indicate the outer curvature and diameter measurements of ascending aortas. (PDF) [file pone.0153811.s013.pdf]

S13 Fig

Control, isotype-matched mouse IgG
(5 mg/kg, 3 times/week)
Saline-infused
Day 0

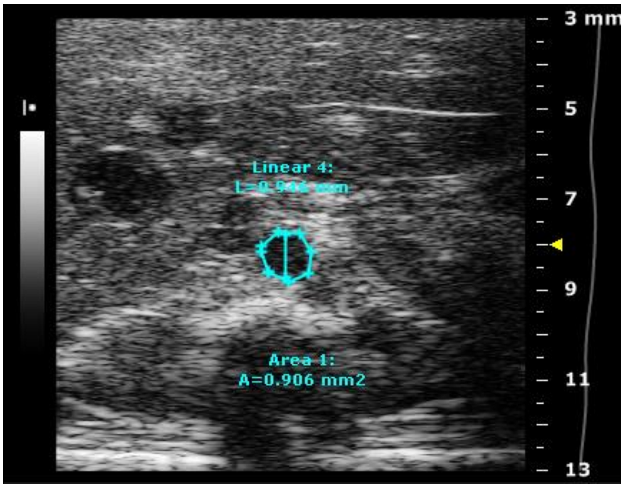


0.95 mm

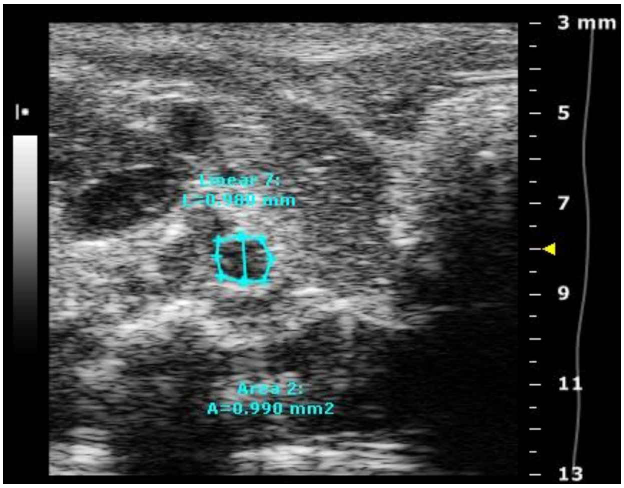


0.98 mm

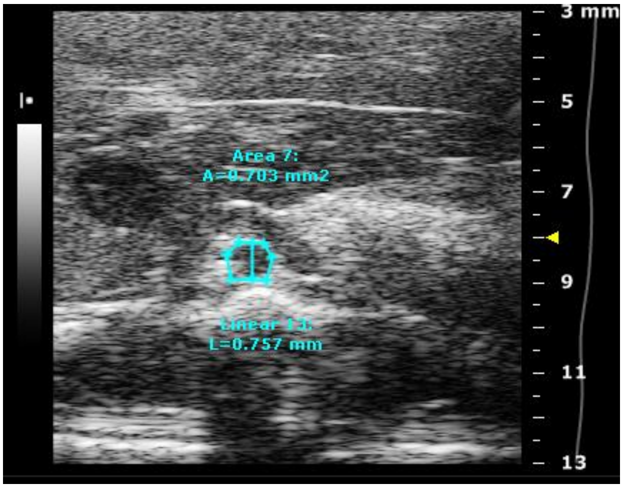


0.76 mm

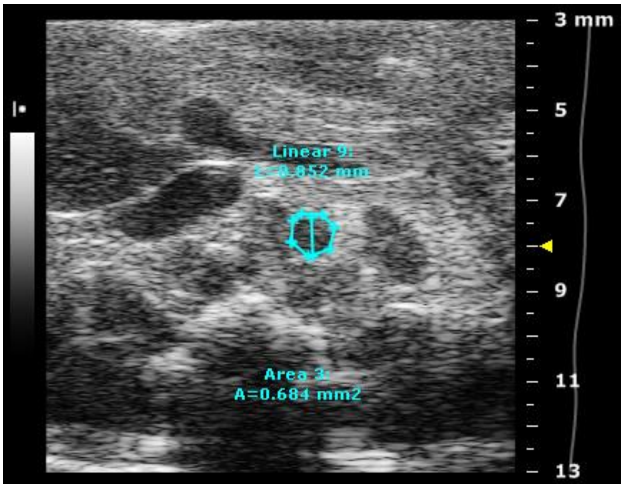


0.85 mm

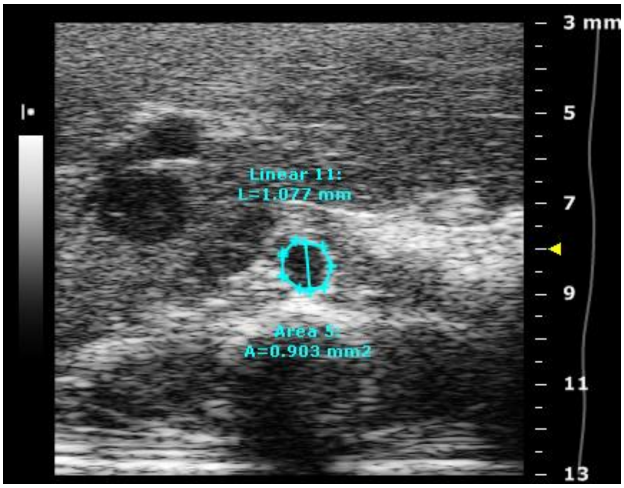


1.08 mm

TGF-β mouse IgG
(5 mg/kg, 3 times/week)
Saline-infused
Day 0

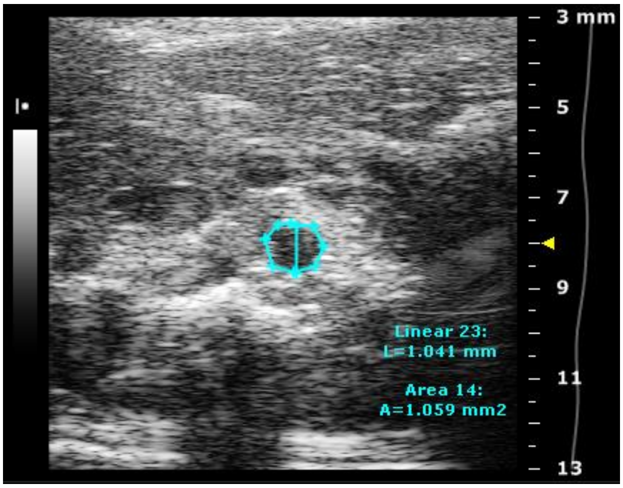


1.04 mm

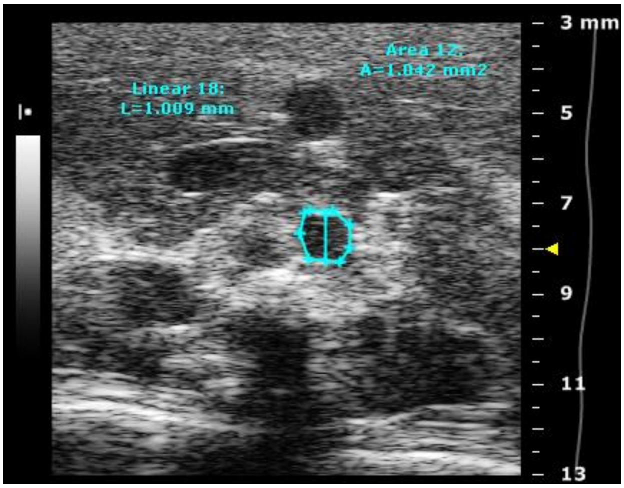


1.01 mm

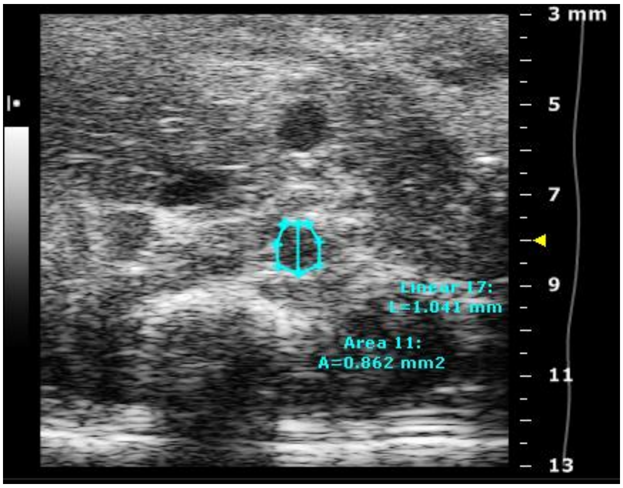


1.04 mm

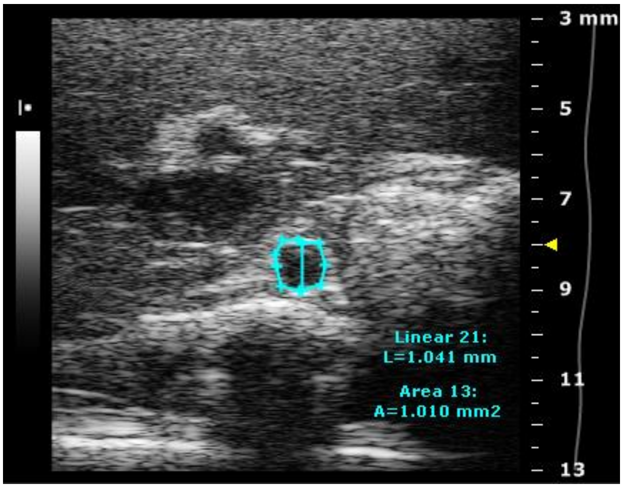


1.04 mm

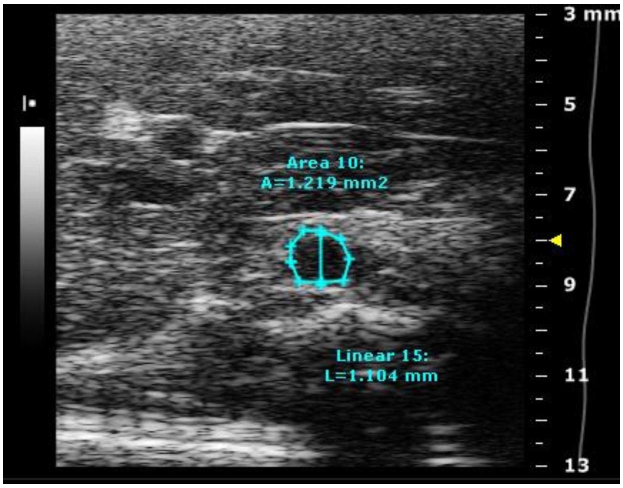


1.01 mm

Control, isotype-matched mouse IgG
(5 mg/kg, 3 times/week)
AngII-infused (1,000 ng/kg/min)
Day 0

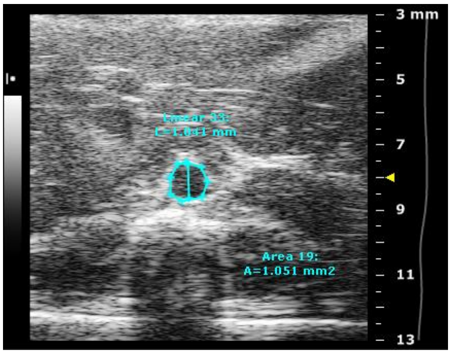


1.04 mm

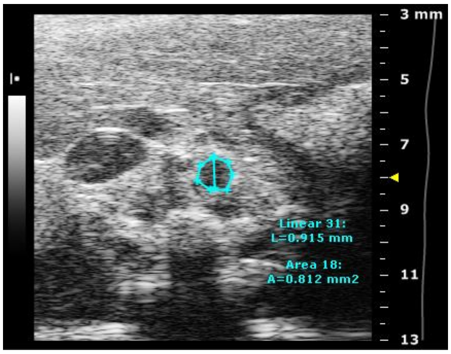


0.92 mm

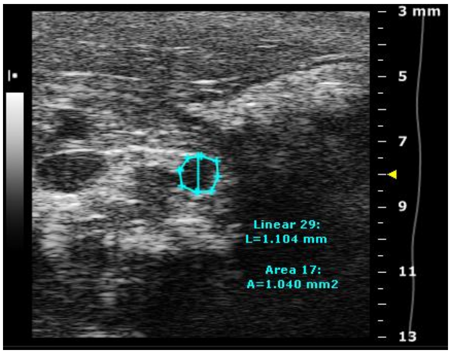


1.10 mm

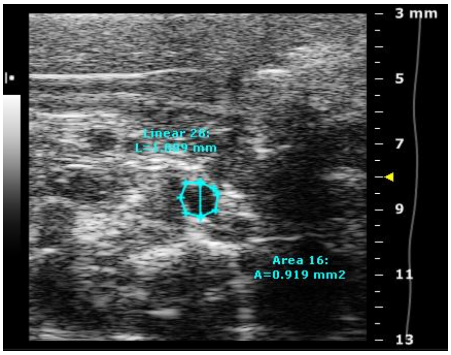


1.00 mm

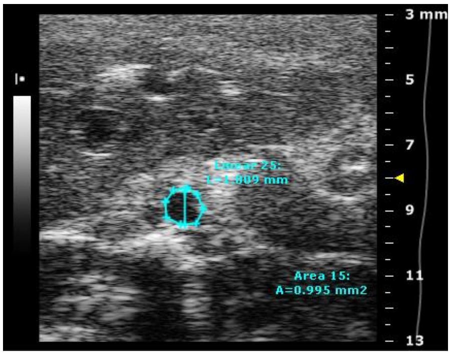


1.00 mm

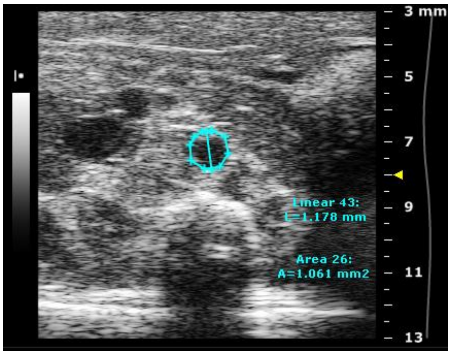


1.18 mm

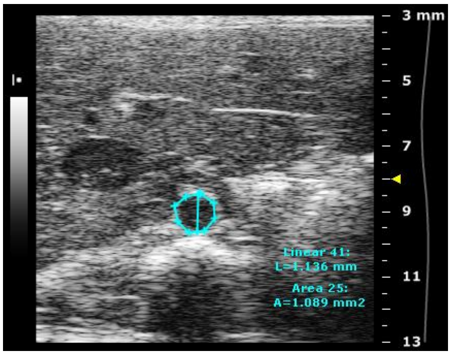


1.14 mm

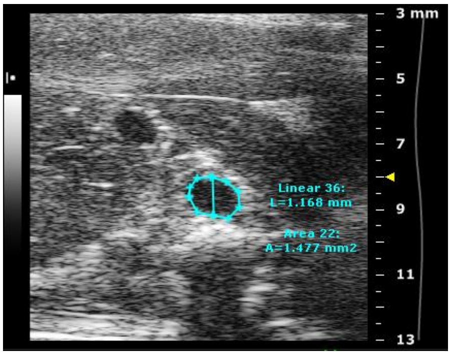


1.17 mm

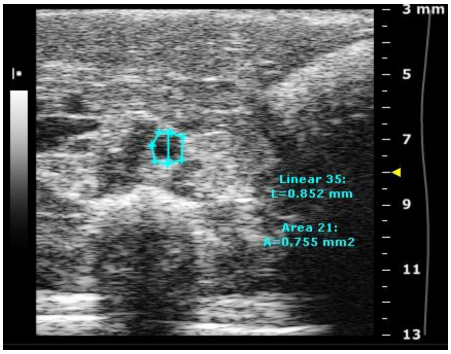


0.85 mm

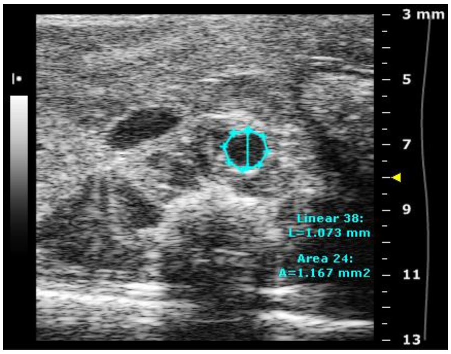


1.07 mm

TGF-β mouse IgG
(5 mg/kg, 3 times/week)
AngII-infused (1,000 ng/kg/min)
Day 0

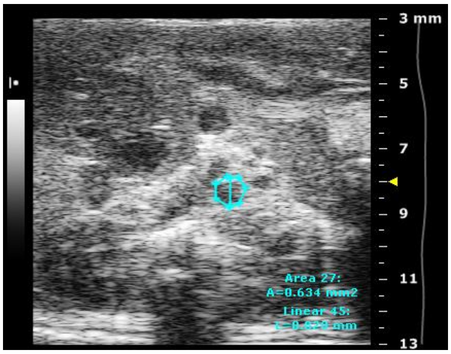


0.82 mm

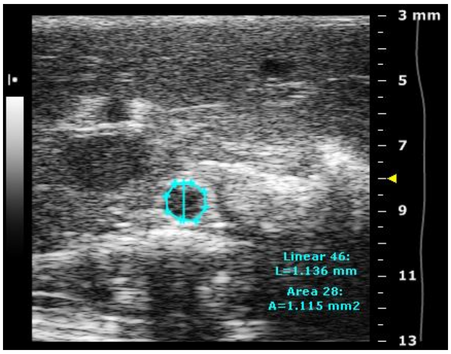


1.14 mm

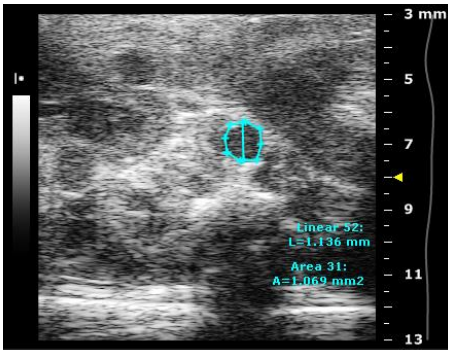


1.14 mm

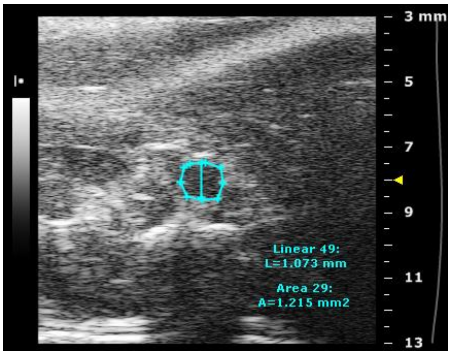


1.07 mm

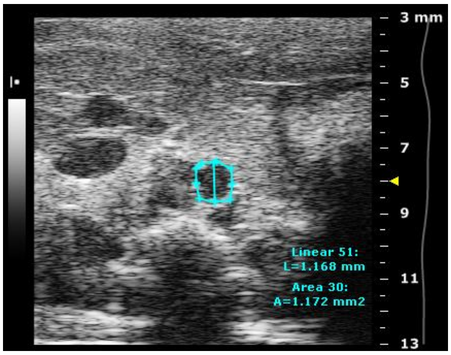


1.17 mm

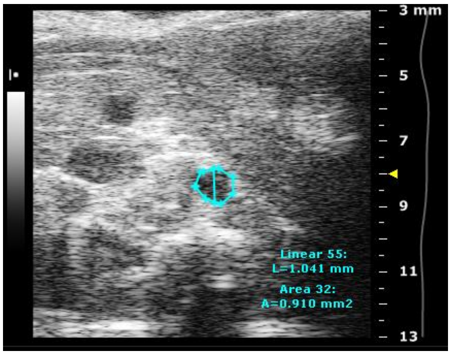


1.04 mm

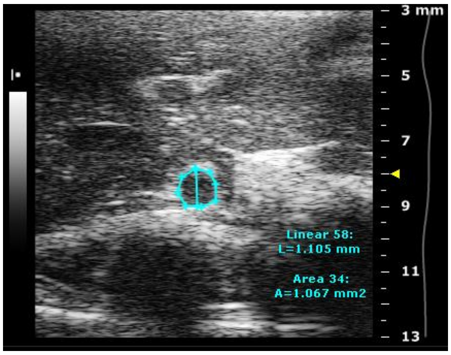


1.12 mm

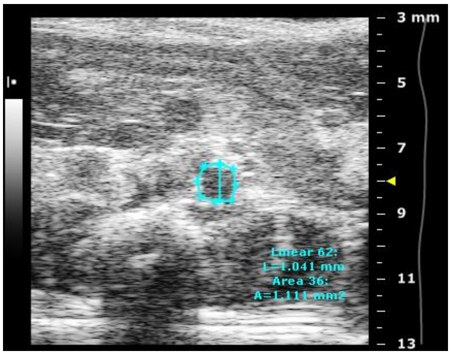


1.04 mm

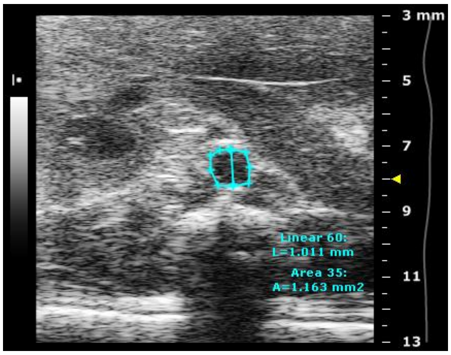


1.00 mm

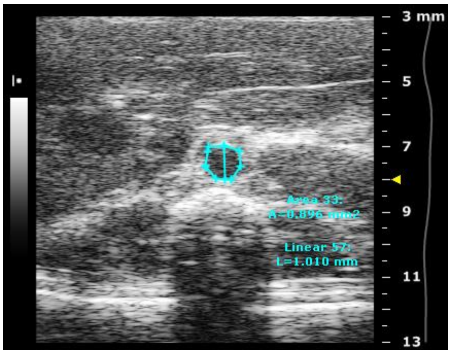


1.01 mm

Control, isotype-matched mouse IgG
(5 mg/kg, 3 times/week)
Saline-infused
Day 4

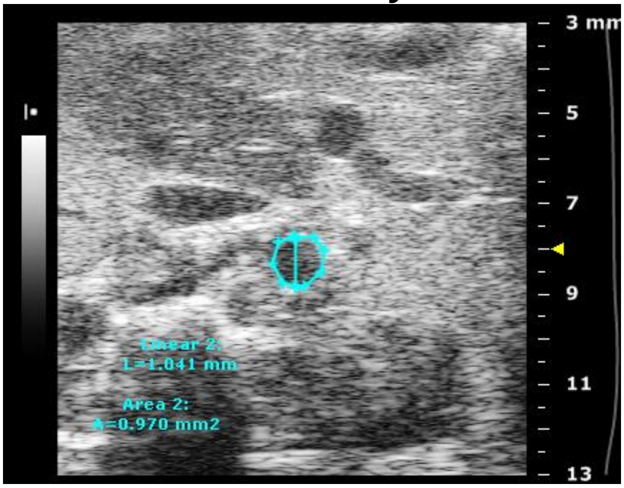


1.04 mm

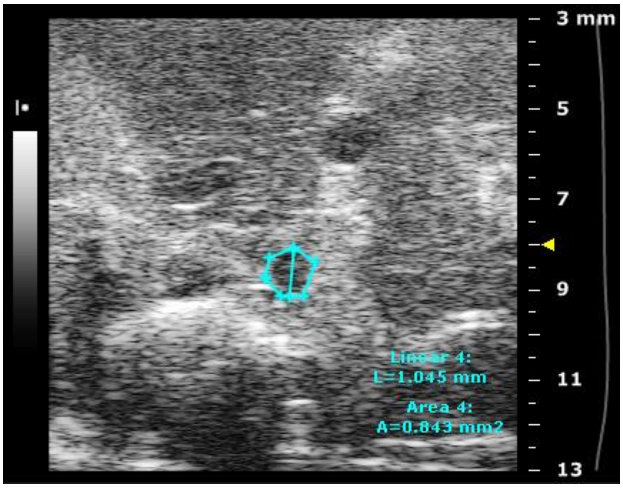


1.05 mm

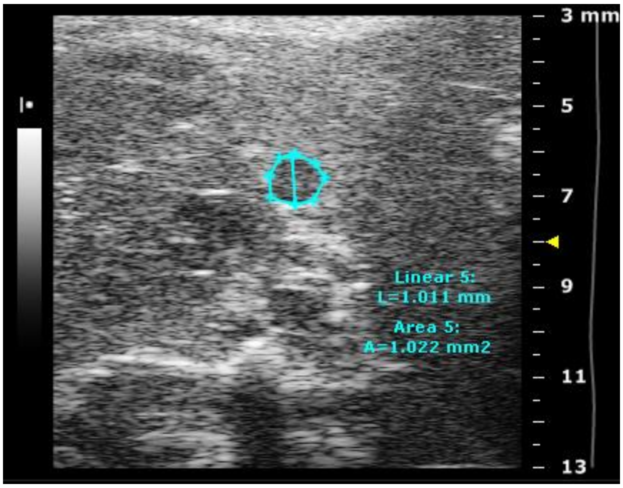


1.01 mm

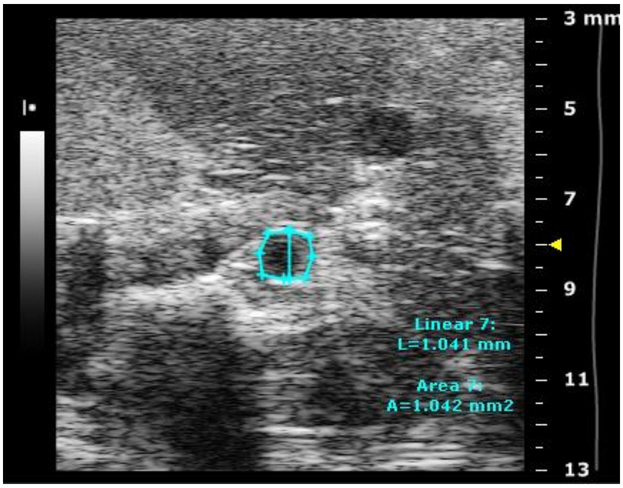


1.04 mm

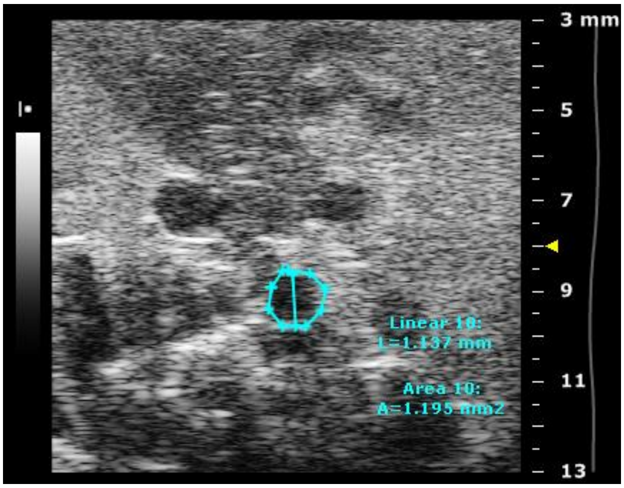


1.14 mm

TGF-β mouse IgG
(5 mg/kg, 3 times/week)
Saline-infused
Day 4

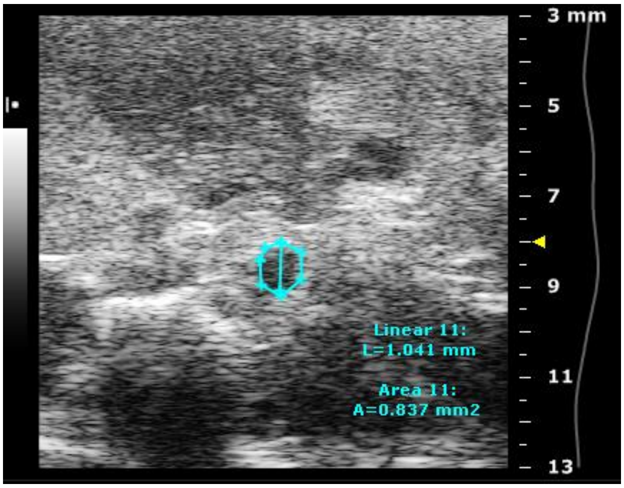


1.04 mm

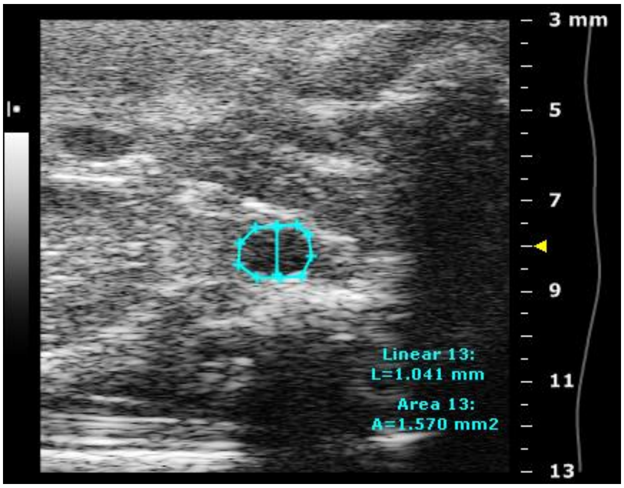


1.57 mm

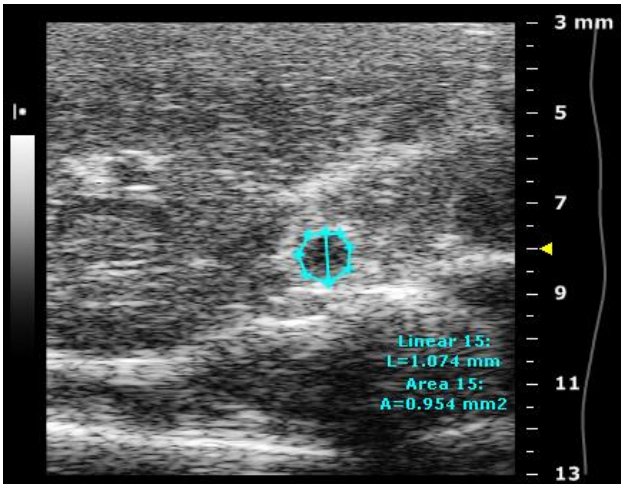


1.07 mm

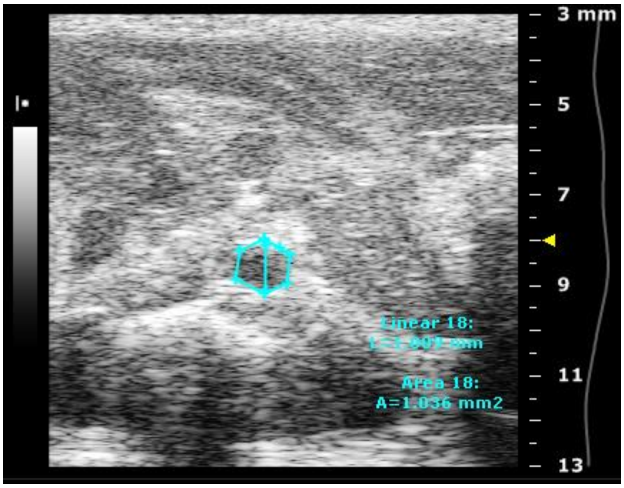


1.00 mm

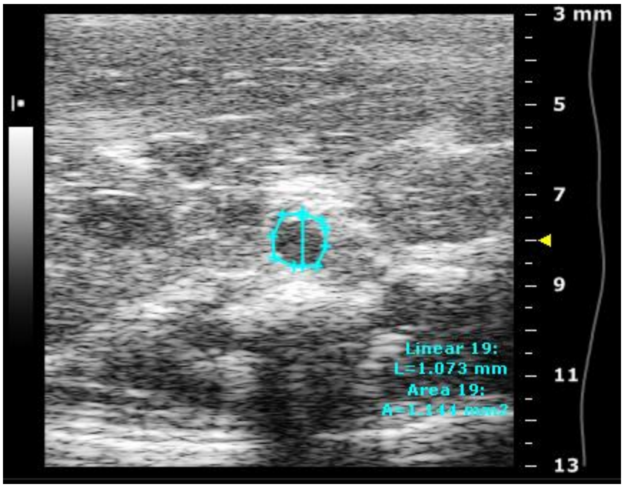


1.07 mm

Control, isotype-matched mouse IgG
(5 mg/kg, 3 times/week)
AngII-infused (1,000 ng/kg/min)
Day 4

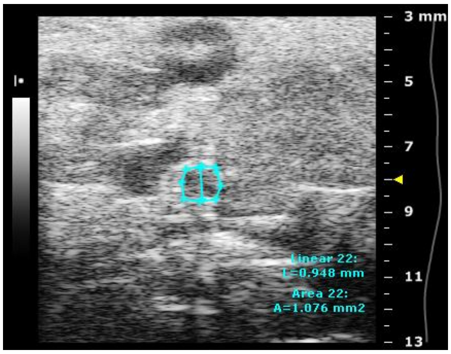


0.95 mm

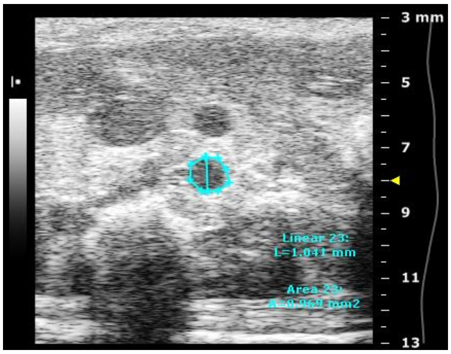


1.04 mm

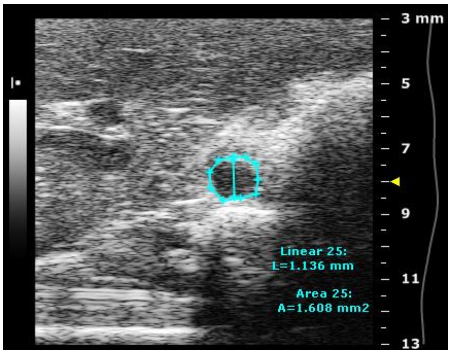


1.14 mm

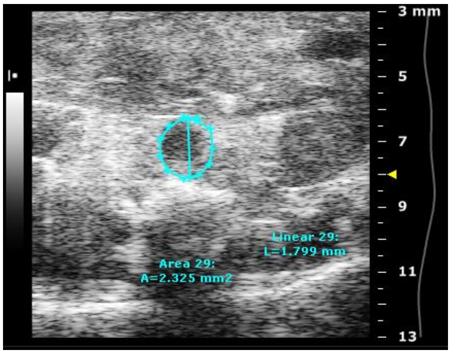


1.80 mm

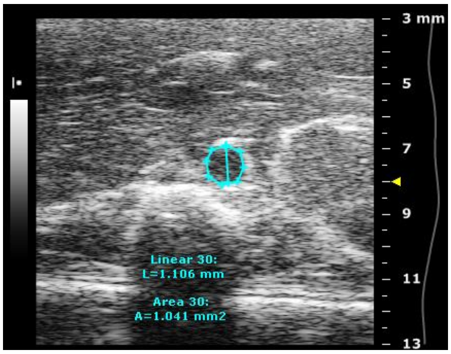


1.10 mm

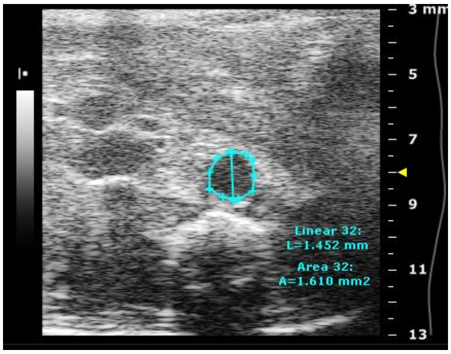


1.45 mm

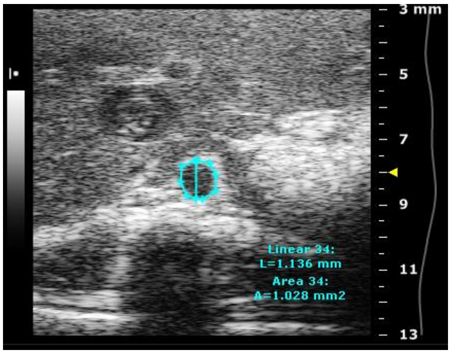


1.13 mm

#18: Died

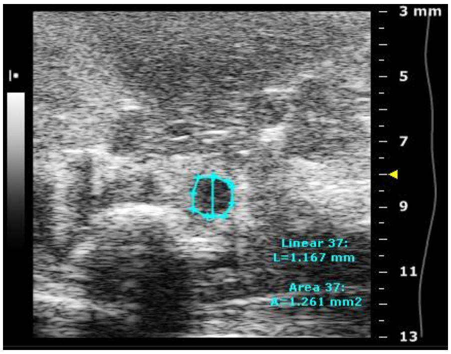


1.17 mm

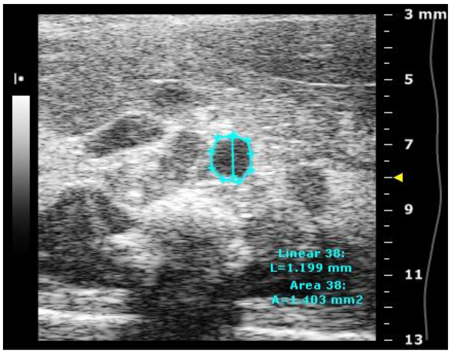


1.20 mm

TGF-β mouse IgG
(5 mg/kg, 3 times/week)
AngII-infused
Day 4

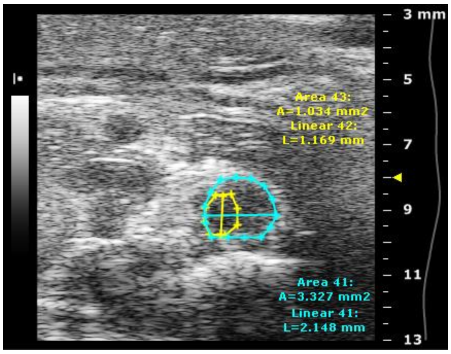


2.15 mm

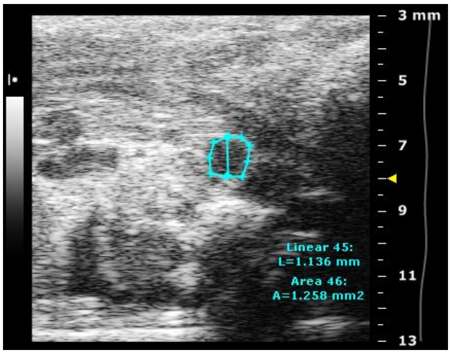


1.14 mm

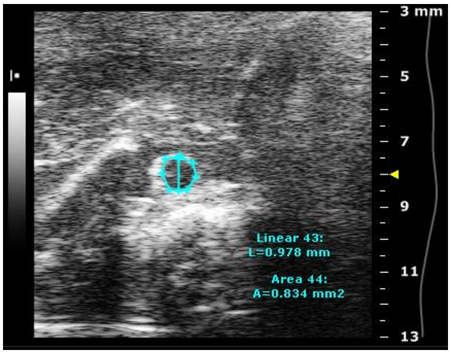


0.98 mm

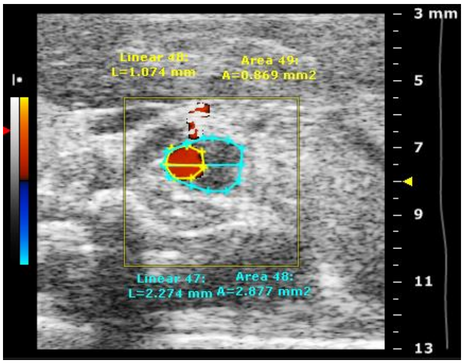


2.27 mm

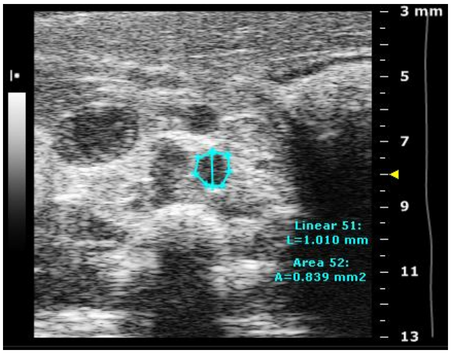


1.01 mm

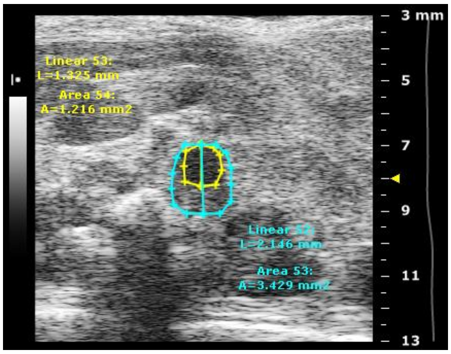


2.15 mm

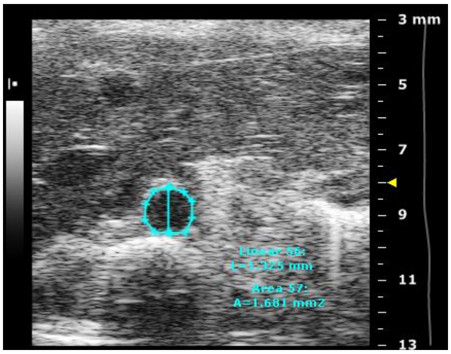


1.32 mm

#28: Died

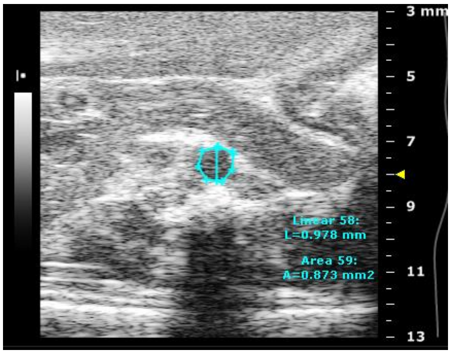


0.98 mm

#30: Died
