# Supplementary material for: TGF-β Neutralization Enhances AngII-Induced Aortic Rupture and Aneurysm in Both Thoracic and Abdominal Regions
Source: PLoS One. 2016 Apr 22;11(4):e0153811. doi: 10.1371/journal.pone.0153811 (PMC4841552; doi:10.1371/journal.pone.0153811)
Supplement: S14 Fig — Numbers below images are ascending aortic area measurements. (PDF) [file pone.0153811.s014.pdf]

S14 Fig

Study #3: Control, isotype-matched IgG (0.3 mg/kg, 3 times/week) AngII-infused (1,000 ng/kg/min)

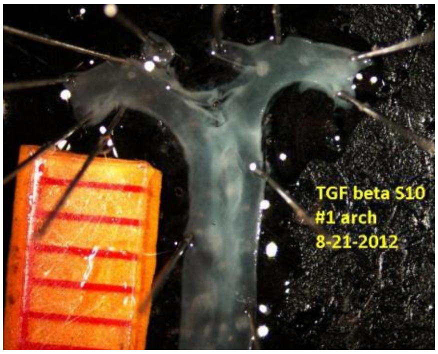

11.6 mm^2

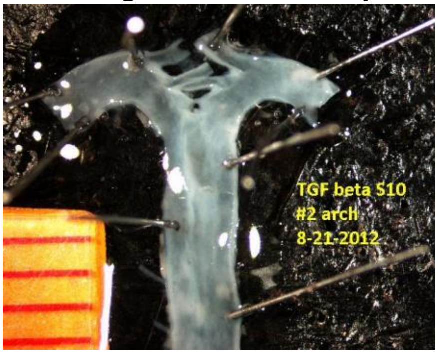

10.8 mm^2

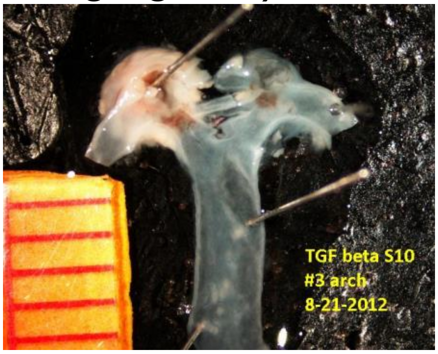

15.5 mm^2

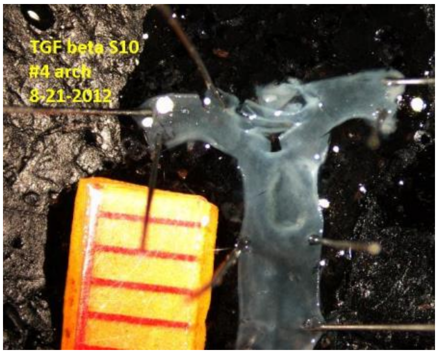

10.1 mm^2

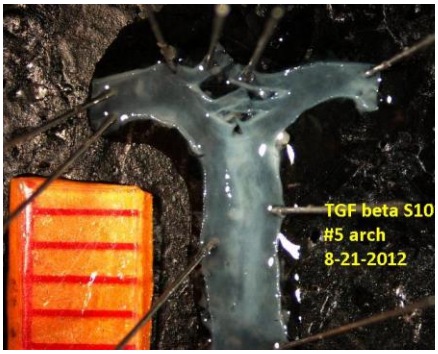

10.3 mm^2

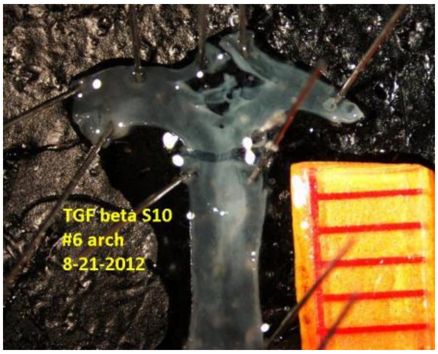

11.3 mm^2

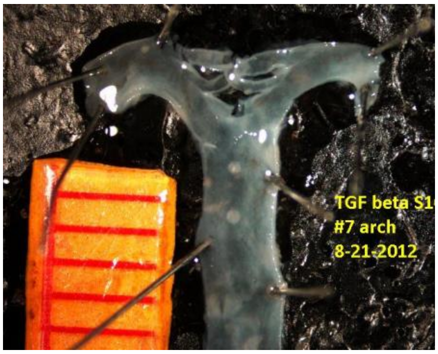

11.0 mm^2

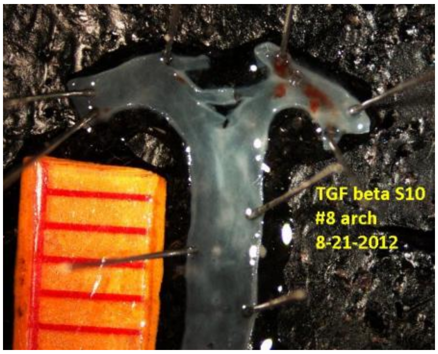

11.5 mm^2

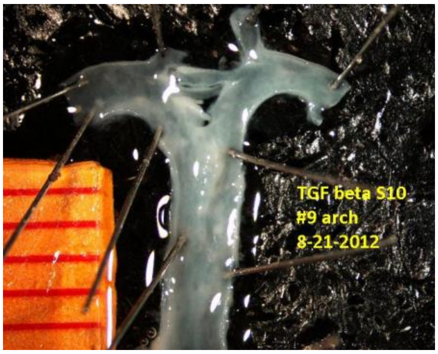

11.4 mm^2

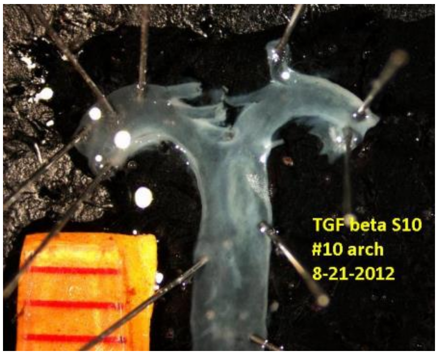

12.2 mm^2

Study #3: TGF-β mouse IgG
(0.3 mg/kg, 3 times/week)
AngII-infused (1,000 ng/kg/min)

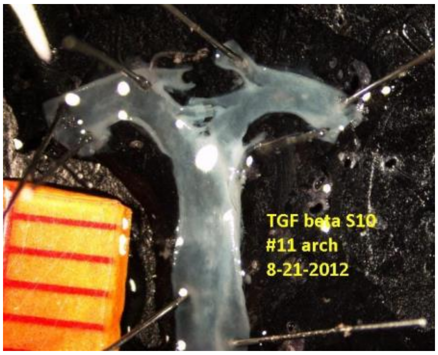

11.6 mm^2

#12: Died

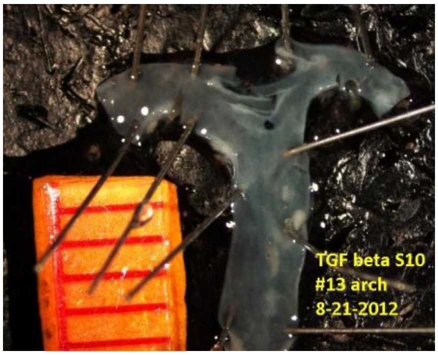

12.1 mm^2

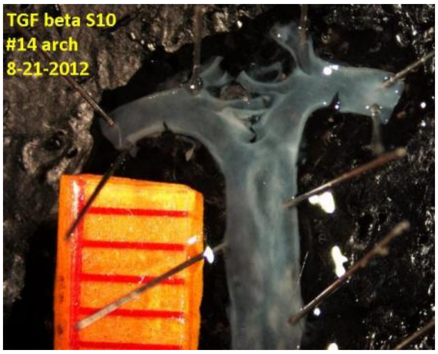

11.2 mm^2

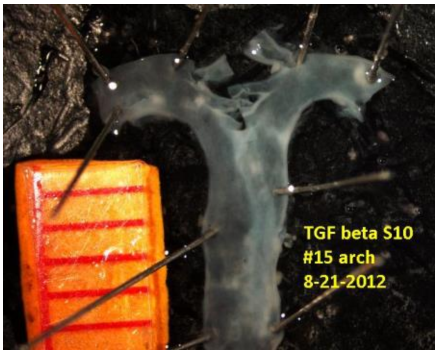

12.0 mm^2

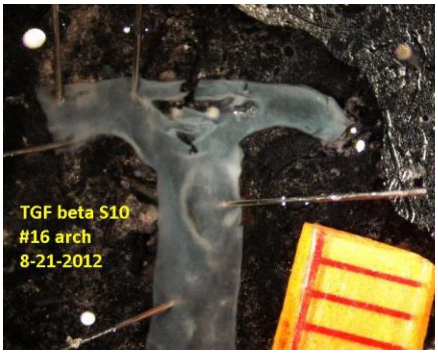

11.8 mm^2

#17: Died

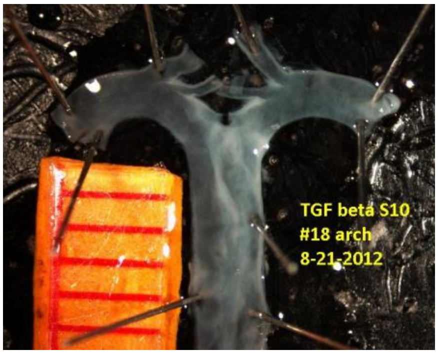

13.1 mm^2

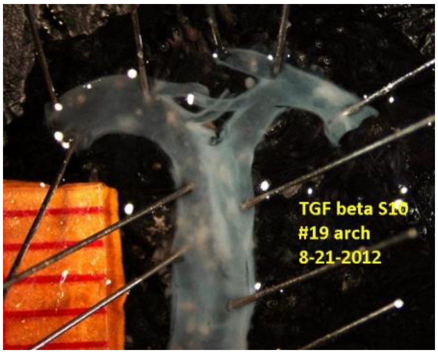

12.5 mm^2

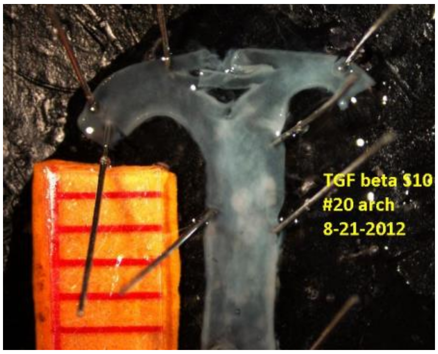

10.9 mm^2

Study #3: Control, isotype-matched IgG
(5 mg/kg, 3 times/week)
AngII-infused (1,000 ng/kg/min)

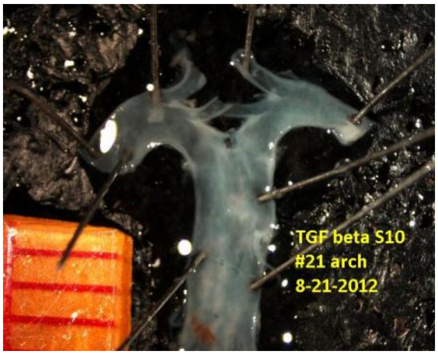

10.3 mm^2

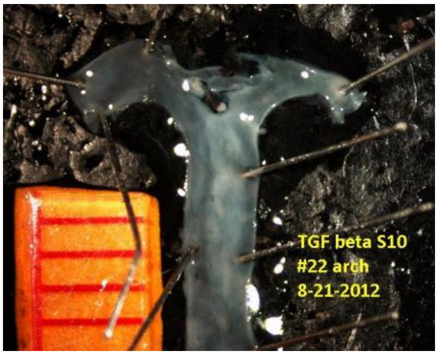

11.2 mm^2

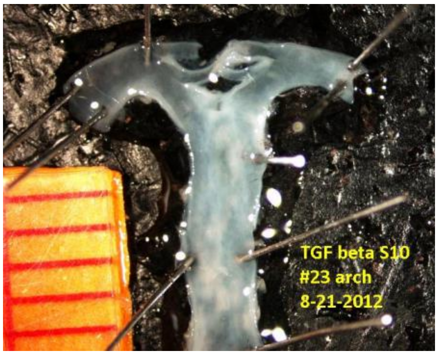

10.9 mm^2

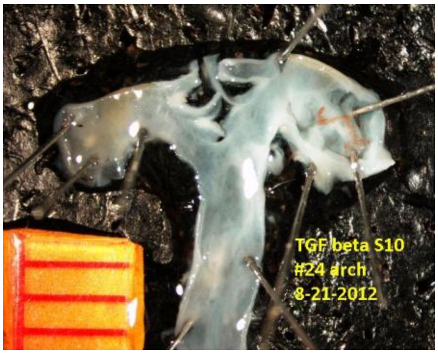

22.0 mm^2

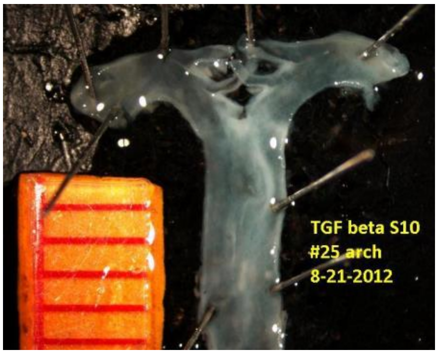

13.7 mm^2

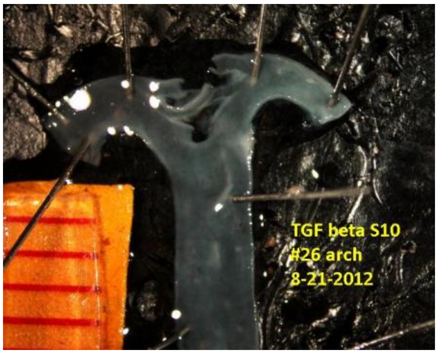

11.8 mm^2

#27: Died

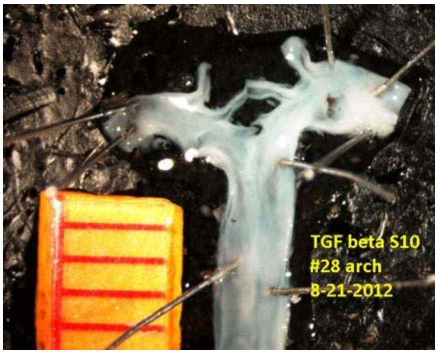

12.4 mm^2

#29: Died

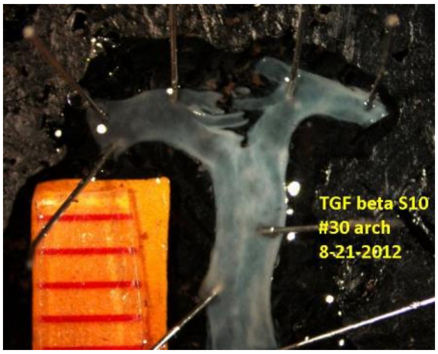

10.3 mm^2

Study #3: TGF-β mouse IgG
(3 mg/kg, 3 times/week)
AngII-infused (1,000 ng/kg/min)

#31: Died

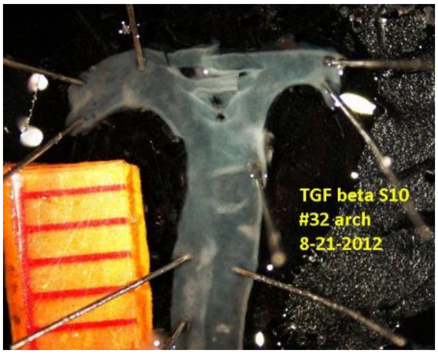

10.5 mm^2

#33: Died

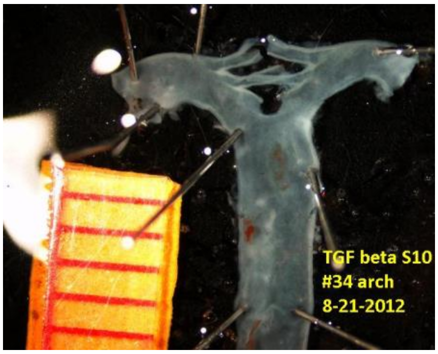

10.8 mm^2

#35:
Not
Available

10.9 mm^2

#36: Died

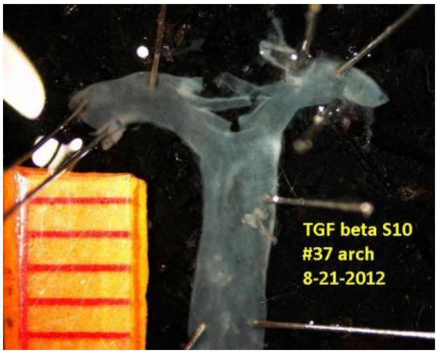

10.6 mm^2

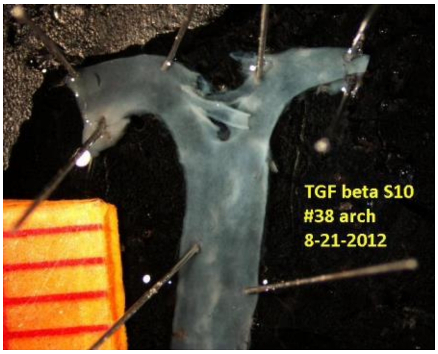

12.8 mm^2

#39: Died

#40: Died
