# Supplementary material for: TGF-β Neutralization Enhances AngII-Induced Aortic Rupture and Aneurysm in Both Thoracic and Abdominal Regions
Source: PLoS One. 2016 Apr 22;11(4):e0153811. doi: 10.1371/journal.pone.0153811 (PMC4841552; doi:10.1371/journal.pone.0153811)
Supplement: S15 Fig — Numbers below images are suprarenal aortic diameter measurements. (PDF) [file pone.0153811.s015.pdf]

S15 Fig

Study #3: Control, isotype-matched IgG (0.3 mg/kg, 3 times/week) AngII-infused (1,000 ng/kg/min)

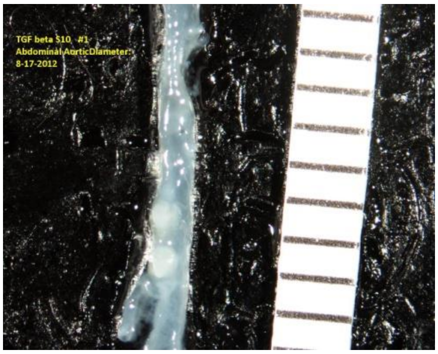

1.07 mm

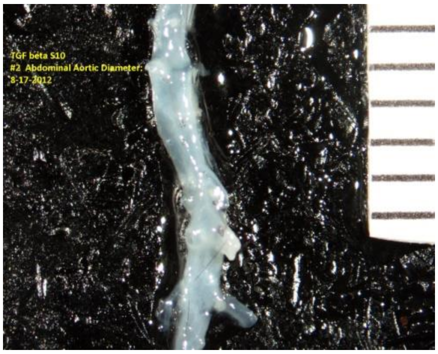

1.06 mm

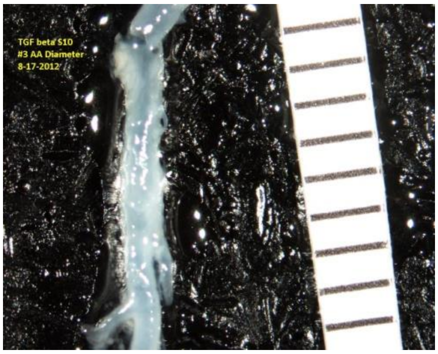

1.03 mm

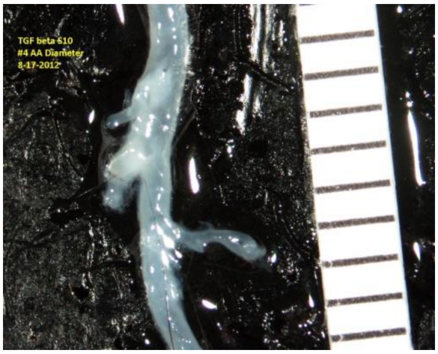

1.06 mm

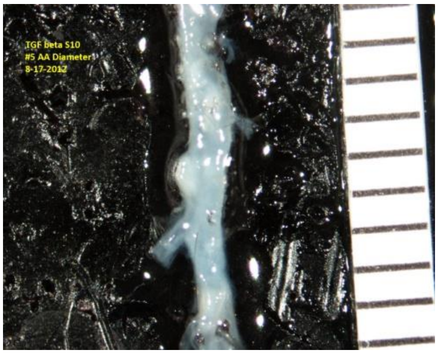

1.21 mm

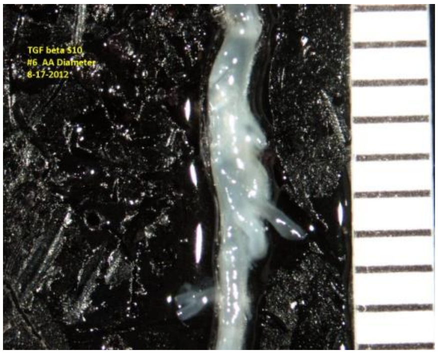

1.07 mm

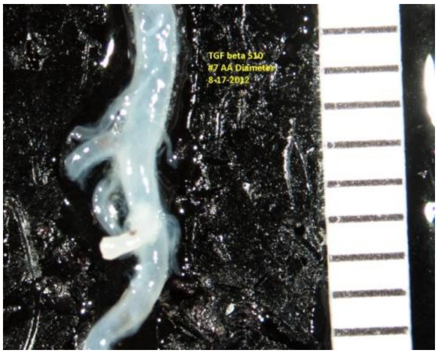

1.08 mm

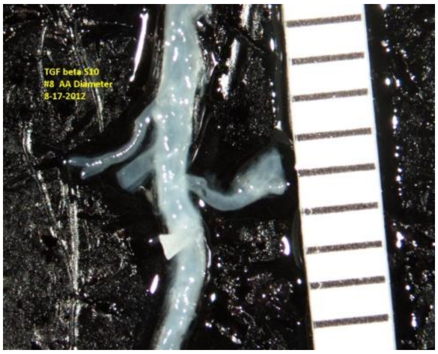

0.96 mm

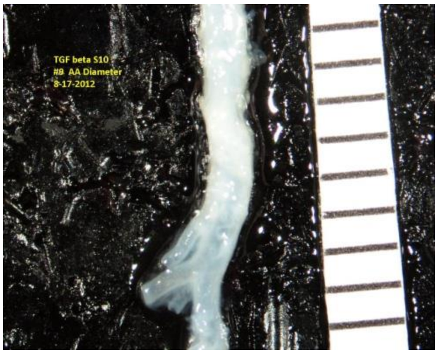

1.19 mm

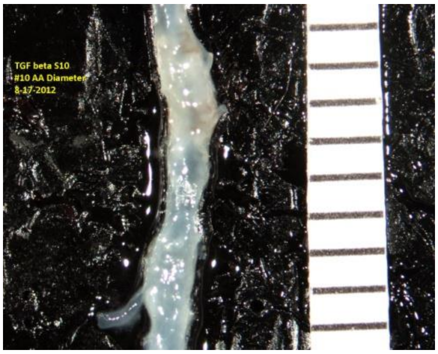

1.35 mm

Study #3: TGF-β mouse IgG
(0.3 mg/kg, 3 times/week)
AngII-infused (1,000 ng/kg/min)

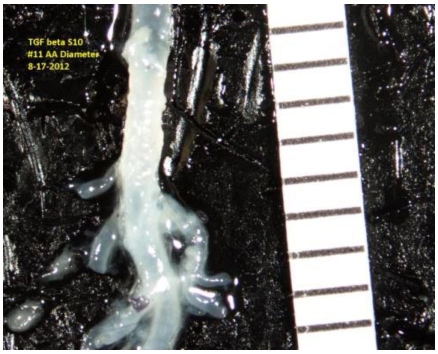

1.08 mm

#12: Died

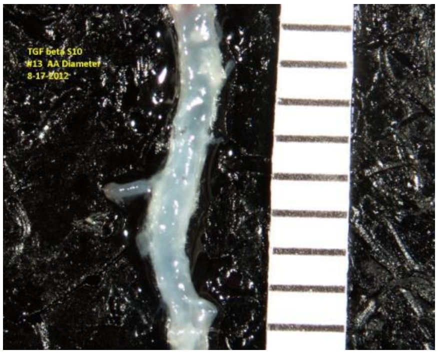

1.05 mm

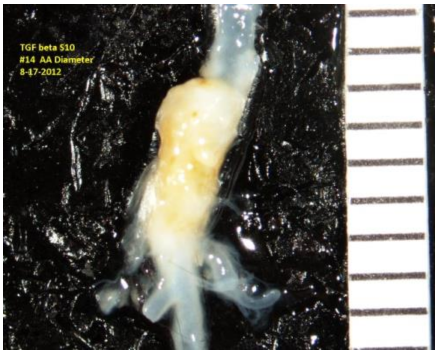

2.13 mm

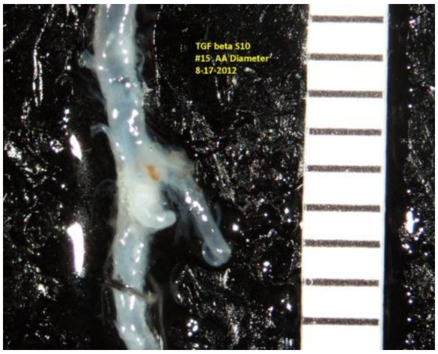

0.98 mm

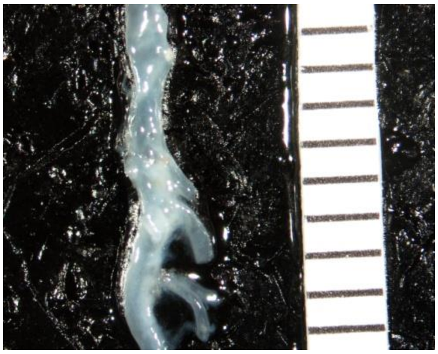

0.98 mm

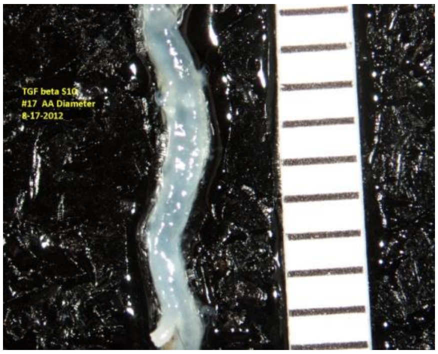

1.03 mm

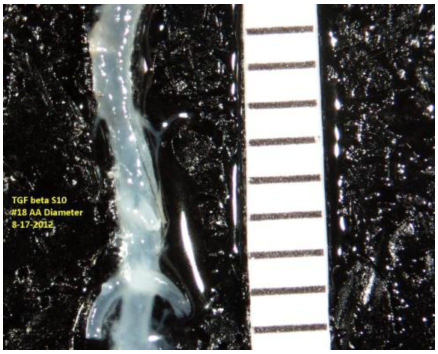

0.95 mm

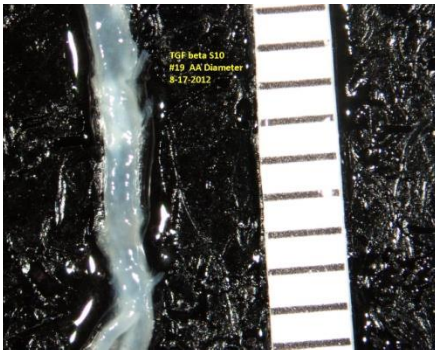

0.93 mm

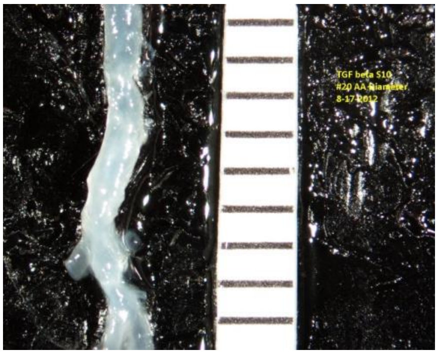

1.05 mm

Study #3: Control, isotype-matched IgG (5 mg/kg, 3 times/week) AngII-infused (1,000 ng/kg/min)

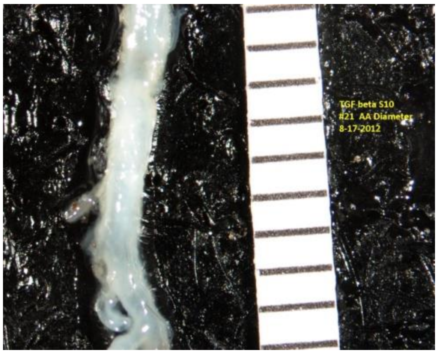

1.20 mm

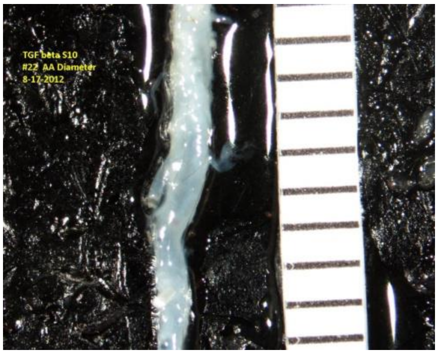

1.05 mm

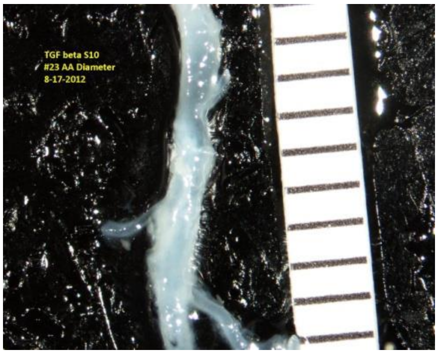

0.96 mm

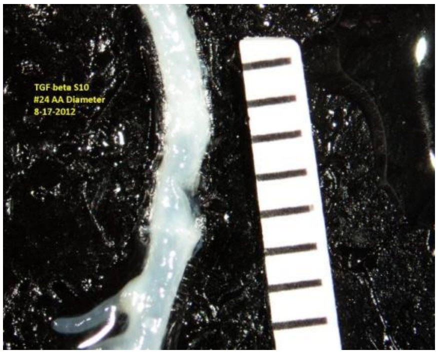

1.28 mm

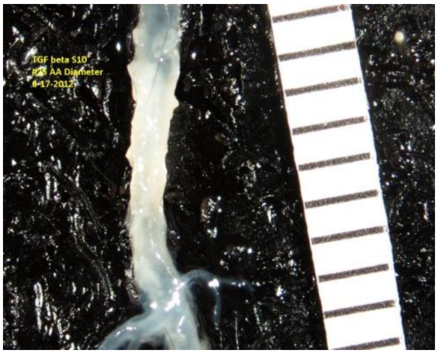

0.98 mm

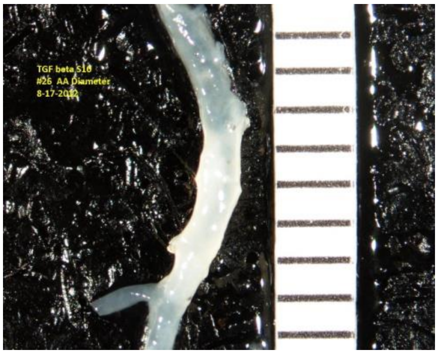

1.02 mm

#27: Died

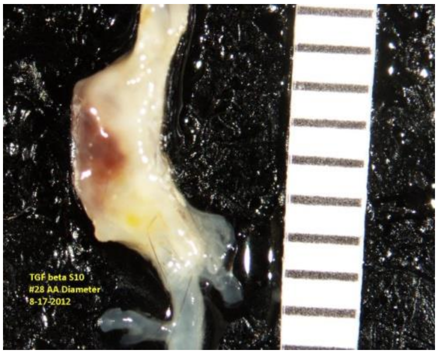

2.51 mm

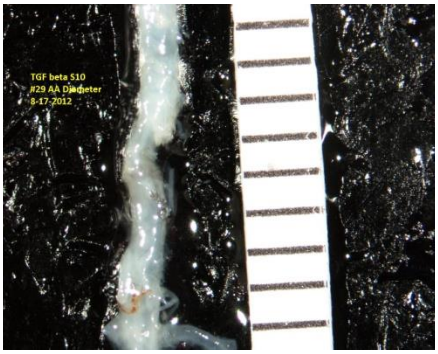

0.92 mm

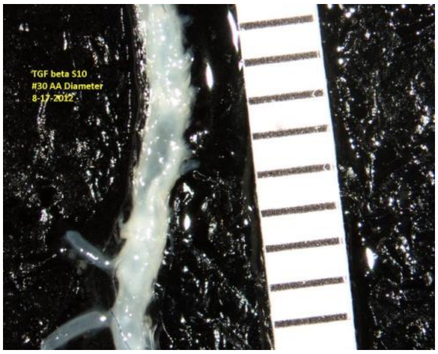

1.02 mm

Study #3: TGF-β mouse IgG
(3 mg/kg, 3 times/week)
AngII-infused (1,000 ng/kg/min)

#31: Died

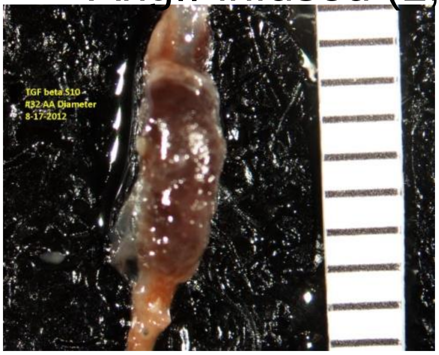

2.29 mm

#33: Died

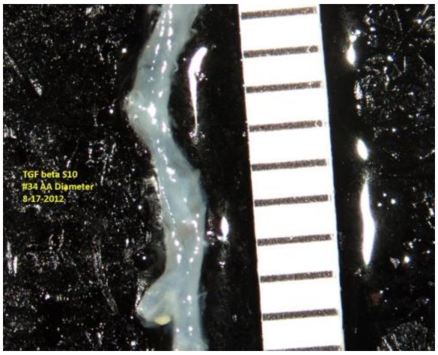

1.13 mm

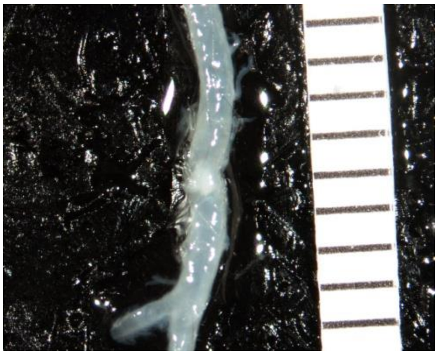

1.06 mm

#36: Died

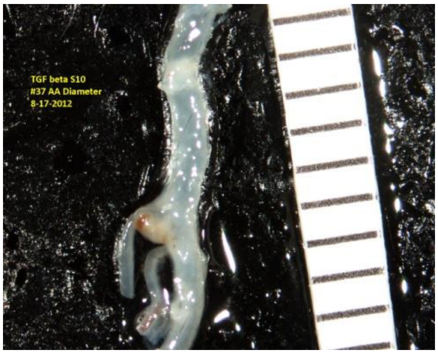

1.50 mm

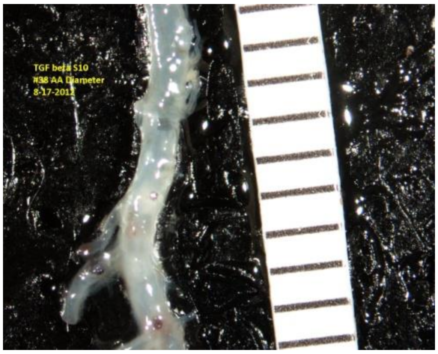

1.06 mm

#39: Died

#40: Died
