# Supplementary material for: TGF-β Neutralization Enhances AngII-Induced Aortic Rupture and Aneurysm in Both Thoracic and Abdominal Regions
Source: PLoS One. 2016 Apr 22;11(4):e0153811. doi: 10.1371/journal.pone.0153811 (PMC4841552; doi:10.1371/journal.pone.0153811)
Supplement: S16 Fig — Mice were infused with AngII for 28 days then injected with mouse TGF-β neutralizing IgG and infused with AngII for an additional 28 days. Numbers below images are ascending aortic area measurements. (PDF) [file pone.0153811.s016.pdf]

S16 Fig

Study #4: Control, isotyped-matched IgG
AngII-infusion (1,000 mg/kg/min)

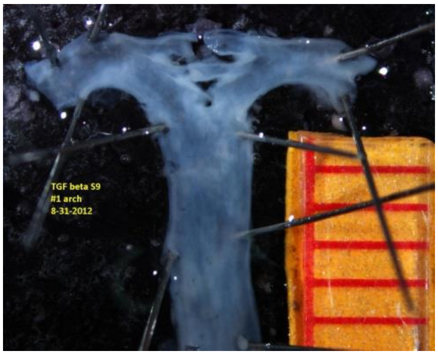

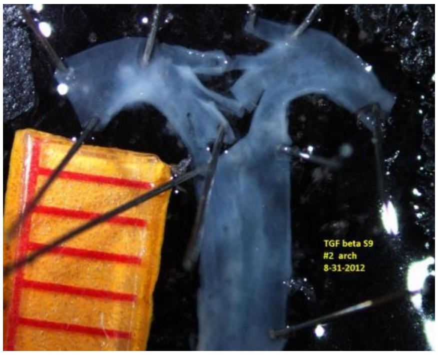

#3:
Not
Available

10.7 mm^2 13.0 mm^2 12.3 mm^2

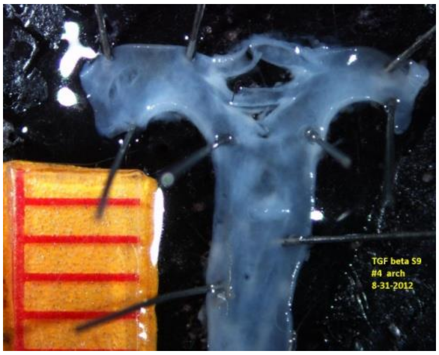

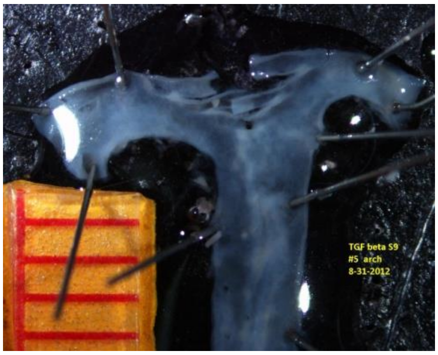

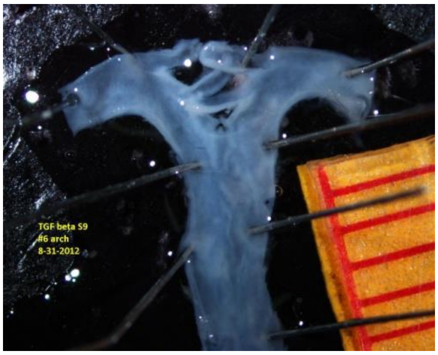

14.3 mm^2 13.9 mm^2 11.1 mm^2

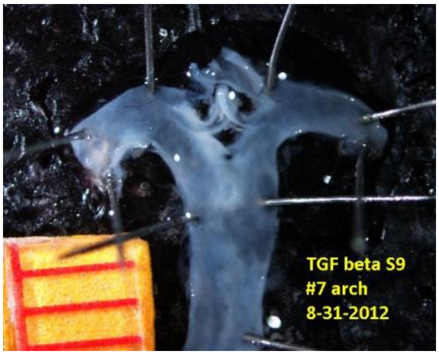

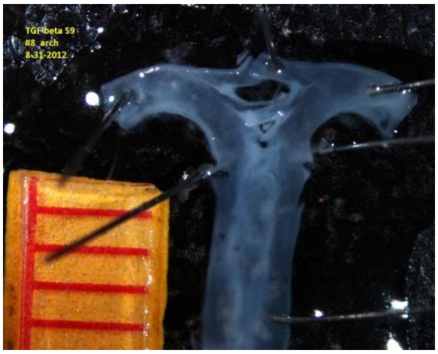

#9: Died

11.2 mm^2 10.1 mm^2

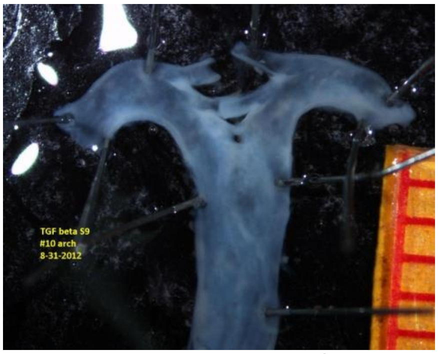

11.4 mm^2

Study #4: TGF-β mouse IgG
AngII-infusion (1,000 mg/kg/min)

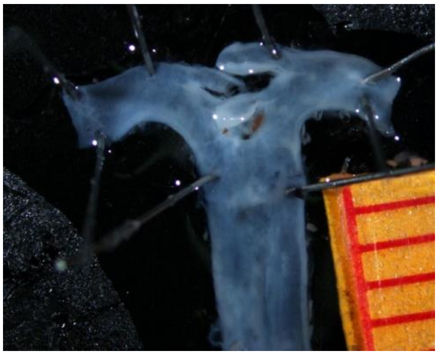

11.8 mm^2

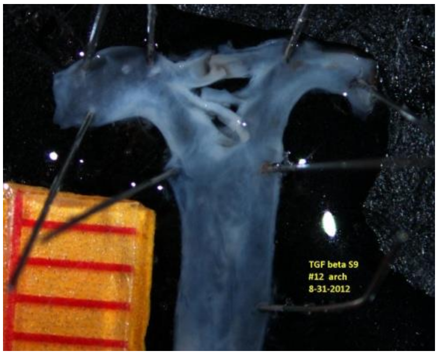

12.3 mm^2

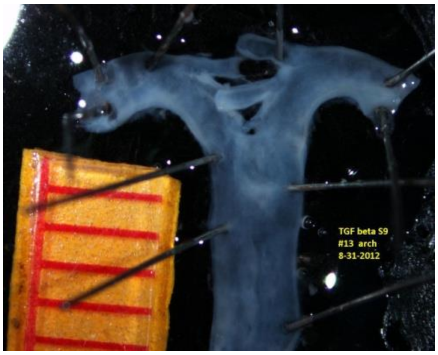

12.1 mm^2

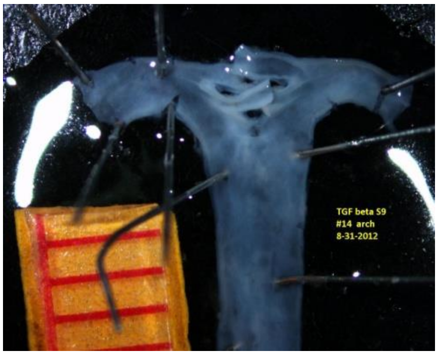

11.3 mm^2

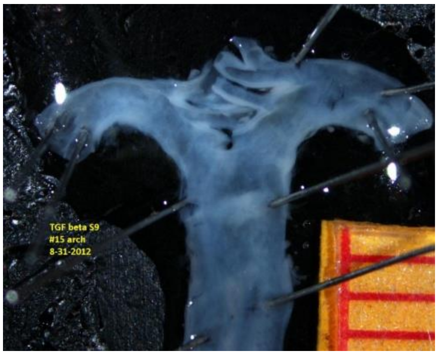

12.9 mm^2

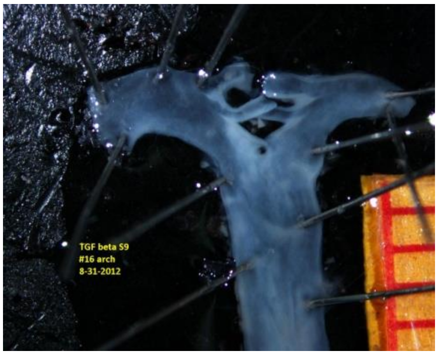

11.0 mm^2

#17: Died

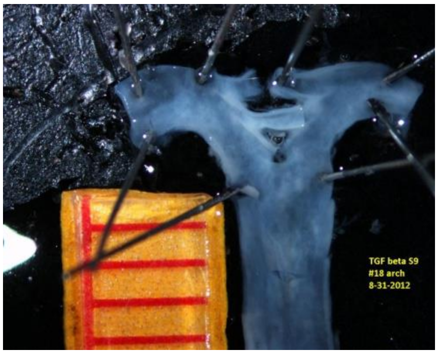

11.4 mm^2

#19: Died

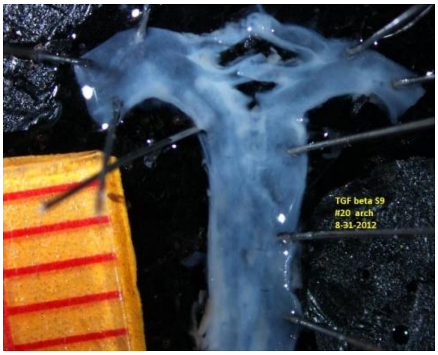

12.4 mm^2
