# Supplementary material for: TGF-β Neutralization Enhances AngII-Induced Aortic Rupture and Aneurysm in Both Thoracic and Abdominal Regions
Source: PLoS One. 2016 Apr 22;11(4):e0153811. doi: 10.1371/journal.pone.0153811 (PMC4841552; doi:10.1371/journal.pone.0153811)
Supplement: S17 Fig — Mice were infused with AngII for 28 days then injected with mouse TGF-β neutralizing IgG and infused with AngII for an additional 28 days. Numbers below images are suprarenal aortic diameter measurements. (PDF) [file pone.0153811.s017.pdf]

S17 Fig

Study #4: Control, isotyped-matched IgG
AngII-infusion (1,000 mg/kg/min)

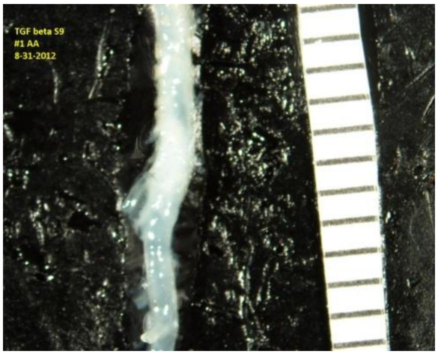

1.24 mm

#2:
Not
Available

1.12 mm

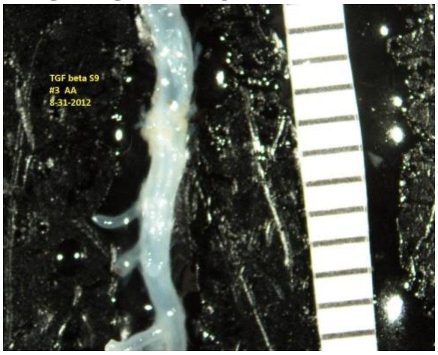

1.11 mm

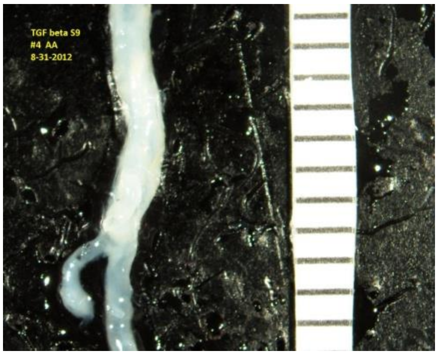

1.16 mm

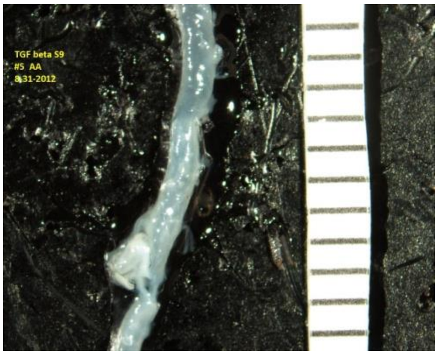

1.13 mm

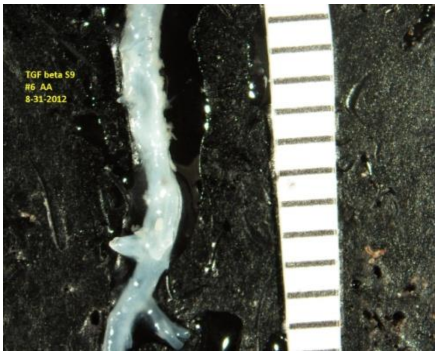

1.15 mm

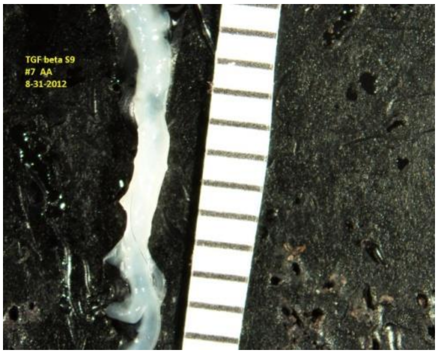

0.88 mm

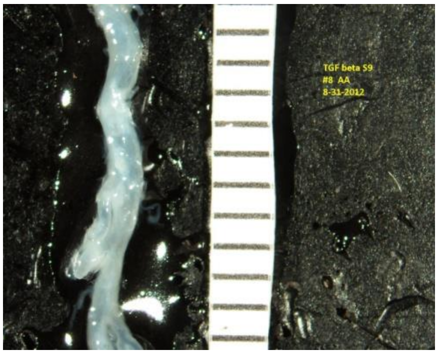

1.10 mm

#9: Died

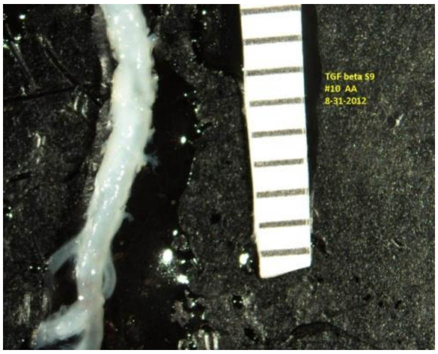

1.19 mm

Study #4: TGF-β mouse IgG
AngII-infusion (1,000 mg/kg/min)

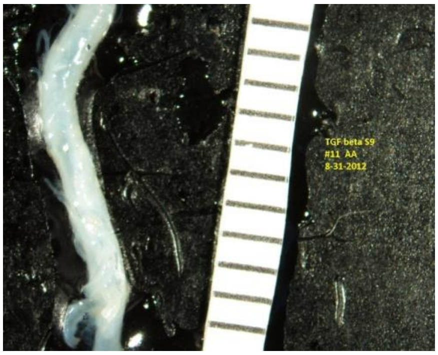

1.19 mm

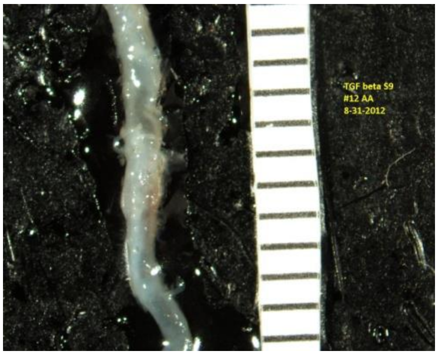

1.36 mm

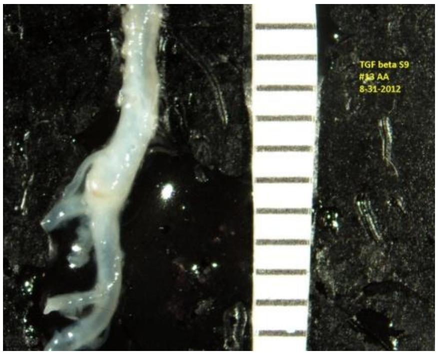

1.54 mm

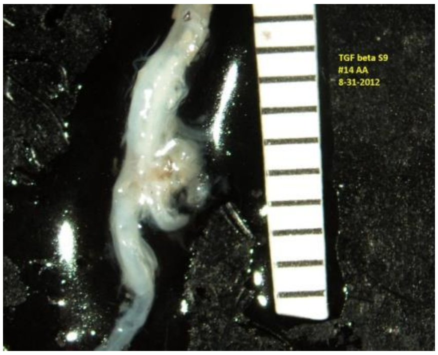

1.94 mm

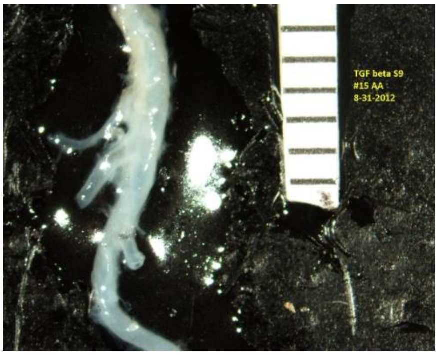

1.12 mm

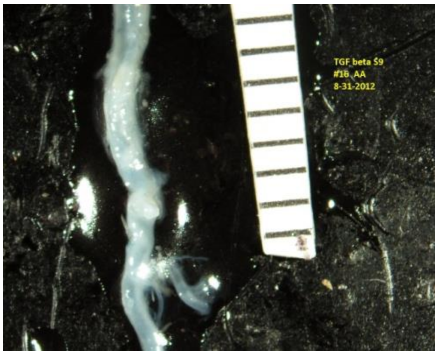

1.06 mm

#17: Died

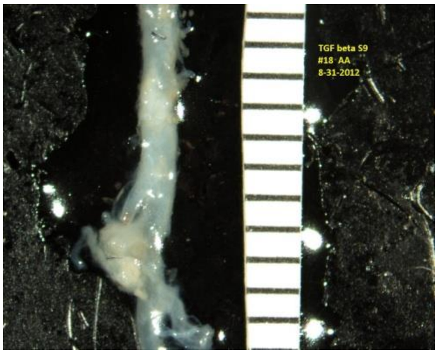

1.23 mm

#19: Died

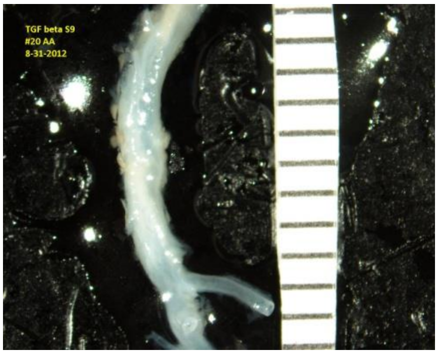

1.30 mm
